# Supplementary material for: Transcriptomic and spatial dissection of human ex vivo right atrial tissue reveals proinflammatory microvascular changes in ischemic heart disease
Source: Cell Rep Med. 2024 May 21;5(5):101556. doi: 10.1016/j.xcrm.2024.101556 (PMC11148807; doi:10.1016/j.xcrm.2024.101556)
Supplement: Document S1. Figures S1–S15 and Tables S1, S2A, and S2B [file mmc1.pdf]

Cell Reports Medicine, Volume *5*

Supplemental information

Transcriptomic and spatial dissection of human *ex vivo* right atrial tissue reveals proinflammatory microvascular changes in ischemic heart disease

Suvi Linna-Kuosmanen, Eloi Schmauch, Kyriakitsa Galani, Johannes Ojanen, Carles A. Boix, Tiit Örd, Anu Toropainen, Prosanta K. Singha, Pierre R. Moreau, Kristiina Harju, Adriana Blazeski, Åsa Segerstolpe, Veikko Lahtinen, Lei Hou, Kai Kang, Elamaran Meibalan, Leandro Z. Agudelo, Hannu Kokki, Jari Halonen, Juho Jalkanen, Jarmo Gunn, Calum A. MacRae, Maija Hollmén, Juha E.K. Hartikainen, Minna U. Kaikkonen, Guillermo García-Cardeña, Pasi Tavi, Tuomas Kiviniemi, and Manolis Kellis

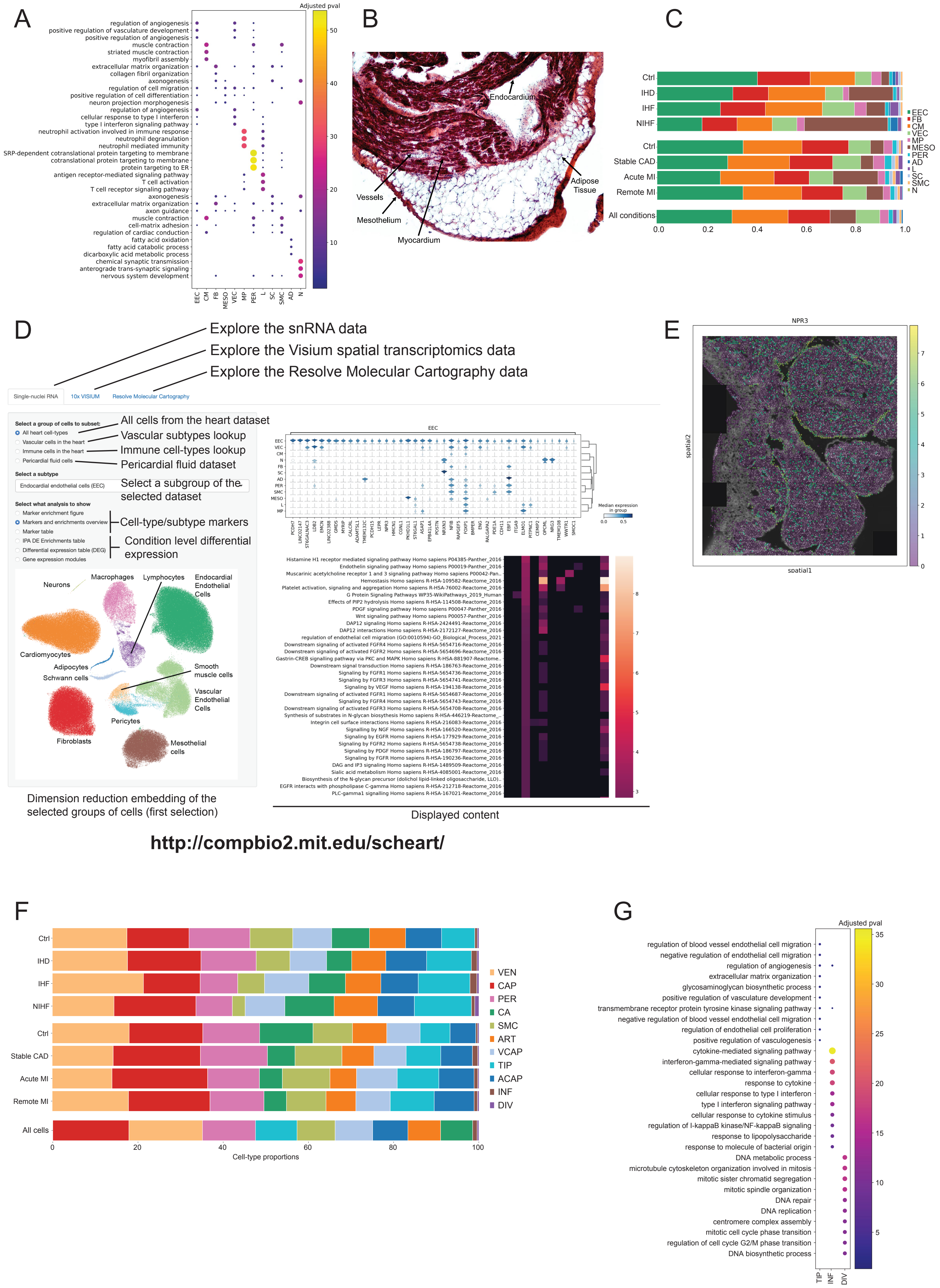
A
Adjusted pval
regulation of angiogenesis
positive regulation of vasculature development
positive regulation of angiogenesis
muscle contraction
striated muscle contraction
myofibril assembly
extracellular matrix organization
collagen fibril organization
axonogenesis
regulation of cell migration
positive regulation of cell differentiation
neuron projection morphogenesis
regulation of angiogenesis
cellular response to type I interferon
type I interferon signaling pathway
neutrophil activation involved in immune response
neutrophil degranulation
neutrophil mediated immunity
SRP-dependent cotranslational protein targeting to membrane
cotranslational protein targeting to membrane
protein targeting to ER
antigen receptor-mediated signaling pathway
T cell activation
T cell receptor signaling pathway
axonogenesis
extracellular matrix organization
axon guidance
muscle contraction
cell-matrix adhesion
regulation of cardiac conduction
fatty acid oxidation
fatty acid catabolic process
dicarboxylic acid metabolic process
chemical synaptic transmission
anterograde trans-synaptic signaling
nervous system development
EEC CM FB MESO VEC MP PER L SC SMC AD N
B
Endocardium
Vessels
Mesothelium
Myocardium
Adipose Tissue
C
Ctrl
IHD
IHF
NIHF
Ctrl
Stable CAD
Acute MI
Remote MI
All conditions
0.0 0.2 0.4 0.6 0.8 1.0
EEC FB CM VEC MP MESO PER AD L SC SMC N
D
Explore the snRNA data
Explore the Visium spatial transcriptomics data
Explore the Resolve Molecular Cartography data
Single-nuclei RNA
10x VISIUM
Resolve Molecular Cartography
Select a group of cells to subset:
All heart cell-types
Vascular cells in the heart
Immune cells in the heart
Pericardial fluid cells
All cells from the heart dataset
Vascular subtypes lookup
Immune cell-types lookup
Pericardial fluid dataset
Select a subtype
Endocardial endothelial cells (EEC)
Select a subgroup of the selected dataset
Select what analysis to show
Marker enrichment figure
Markers and enrichments overview
Marker table
IPA DE Enrichments table
Differential expression table (DEG)
Gene expression modules
Cell-type/subtype markers
Condition level differential expression
Neurons
Macrophages
Lymphocytes
Endocardial Endothelial Cells
Cardiomyocytes
Adipocytes
Schwann cells
Smooth muscle cells
Vascular Endothelial Cells
Pericytes
Fibroblasts
Mesothelial cells
Dimension reduction embedding of the selected groups of cells (first selection)
EEC
Median expression in group
Displayed content
http://compbio2.mit.edu/scheart/
E
NPR3
spatial2
spatial1
F
Ctrl
IHD
IHF
NIHF
Ctrl
Stable CAD
Acute MI
Remote MI
All cells
0 20 40 60 80 100
Cell-type proportions
VEN CAP PER CA SMC ART VCAP TIP ACAP INF DIV
G
Adjusted pval
regulation of blood vessel endothelial cell migration
negative regulation of endothelial cell migration
regulation of angiogenesis
extracellular matrix organization
glycosaminoglycan biosynthetic process
positive regulation of vasculature development
transmembrane receptor protein tyrosine kinase signaling pathway
negative regulation of blood vessel endothelial cell migration
regulation of endothelial cell proliferation
positive regulation of vasculogenesis
cytokine-mediated signaling pathway
interferon-gamma-mediated signaling pathway
cellular response to interferon-gamma
response to cytokine
cellular response to type I interferon
type I interferon signaling pathway
cellular response to cytokine stimulus
regulation of I-kappaB kinase/NF-kappaB signaling
response to lipopolysaccharide
response to molecule of bacterial origin
DNA metabolic process
microtubule cytoskeleton organization involved in mitosis
mitotic sister chromatid segregation
mitotic spindle organization
DNA repair
DNA replication
centromere complex assembly
mitotic cell cycle phase transition
regulation of cell cycle G2/M phase transition
DNA biosynthetic process
TIP INF DIV

Fig. S1. Heart cell-types and subtypes distribution and enrichments. Related to Figures 1 and 2.

A. Top 3 enriched gene sets (GO Biological Process) for top 1000 genes with FDR < 0.05 for each cell type. Dot color and size is -log10(adjusted p-value). Only significant (adjusted p-value < 0.05) enrichments are shown. **B.** Hematoxylin-eosin-stained histology slide of a right atrial appendage sample from a patient with IHD (included in the dataset). **C.** Proportion of the main cell types, per condition. **D.** Interactive website overview. An interactive website is accessible at http://compbio.mit.edu/scheart. It provides access to the analyzed snRNA-seq and spatial transcriptomics data (both Visium and Resolve Molecular Cartography) presented in this study. The user can select the ensemble of cells to focus on (all heart cells, vascular heart cells, immune heart cells, pericardial fluid data) and explore different information types (1. Gene set enrichment of subtype/cell-type marker genes 2. Subtype/cell-type marker gene overview. 3. Subtype/cell-type marker genes table. 4. IPA enrichment table from differential expression result of a selected condition in the subtype/cell-type. 5. Differential expression result table of a selected condition in the subtype/cell-type. 6. Gene expression module analysis in the selected subtype/cell-type). **E.** Spatial expression of *NPR3* on a Resolve molecular cartography section of the right atrium in IHD sample. **F.** Proportion of vascular cells, per condition. **G**. Top 10 enriched gene sets (GO Biological Process) for top 200 genes with FDR < 0.05 in each subtype, for tip cells (TIP), inflammatory endothelial cells (INF) and dividing endothelial cells (DIV). Dot color and size is -log10(adjusted enrichment p-value). Only significant (adjusted p-value < 0.05) enrichments are shown.

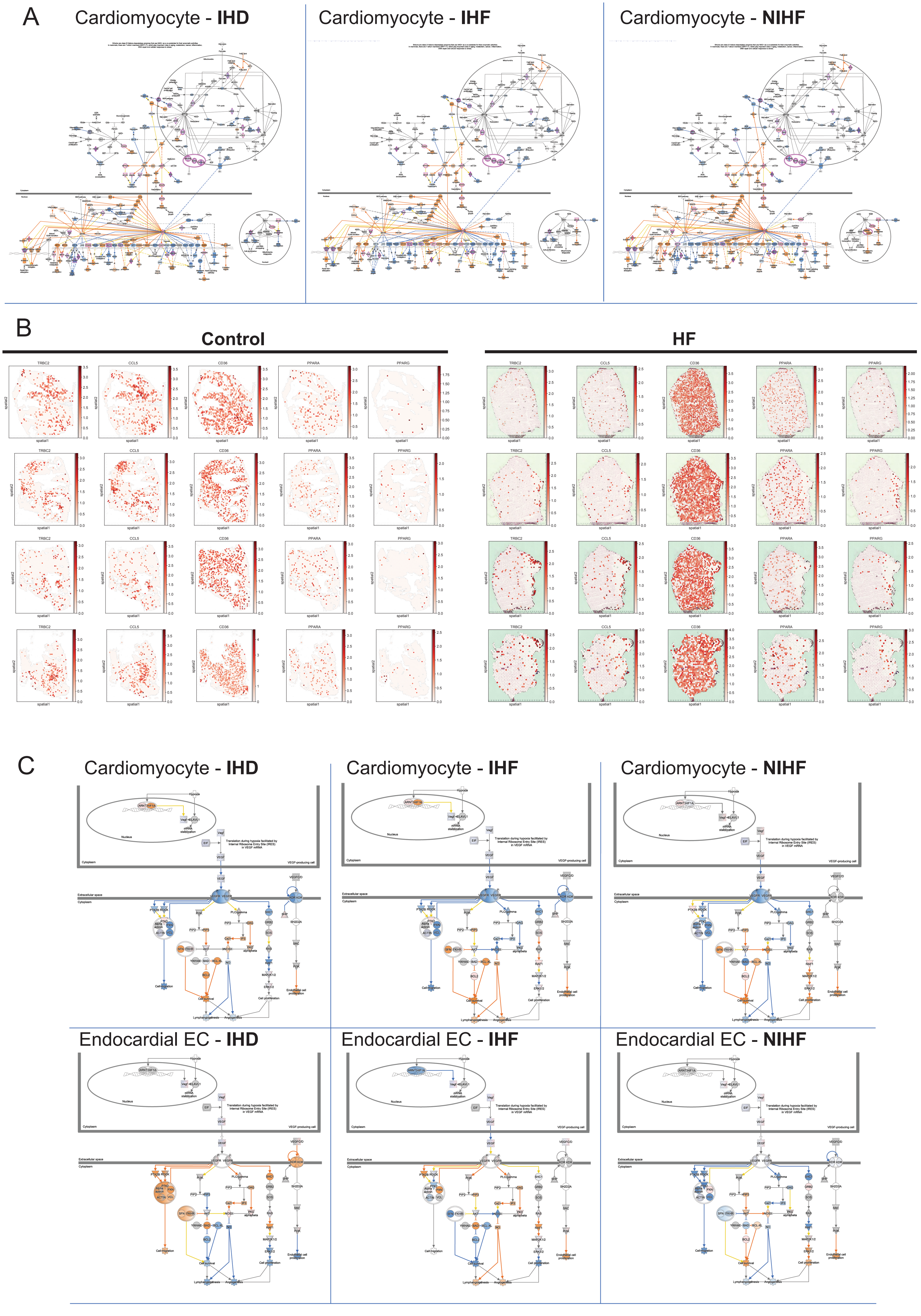
A
Cardiomyocyte - IHD
Cardiomyocyte - IHF
Cardiomyocyte - NIHF
B
Control
HF
TRBC2
CCL5
CD36
PPARA
PPARG
spatial1
spatial2
C
Cardiomyocyte - IHD
Cardiomyocyte - IHF
Cardiomyocyte - NIHF
Endocardial EC - IHD
Endocardial EC - IHF
Endocardial EC - NIHF

Fig. S2. Signaling pathway activity enrichment across condition and spatial transcriptomics gene expression changes. Related to Figure 3.

A. Sirtuin signaling pathway from IPA in cardiomyocytes in IHD, IHF, and NIHF. Colors indicate measured and inferred expression/activity changes. Blue stands for downregulation or inhibition, orange/red for upregulation. Yellow line indicates inconsistent changes and purple highlights measured significant changes in the dataset. **B.** Expression of *TRBC2*, *CCL5*, *CD36*, *PPARA*, *PPARG* on Visium slides, from a patient with heart failure and a control sample (n=4). **C.** VEGF signaling pathway from IPA in cardiomyocytes and endocardial endothelial cells in IHD, IHF, and NIHF. Blue stands for downregulation or inhibition of the molecule, orange/red for upregulation. Yellow line indicates inconsistent changes.

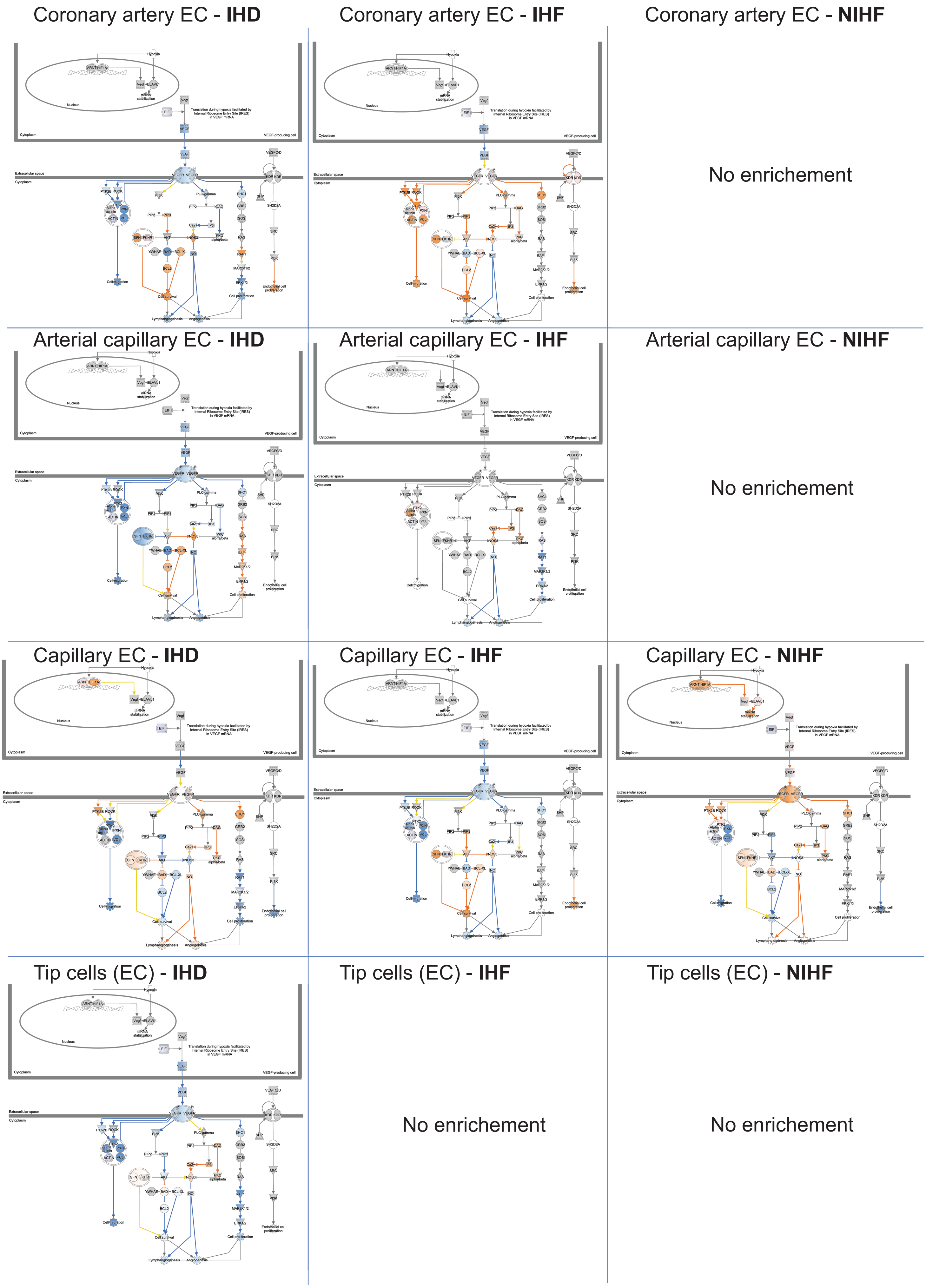

Coronary artery EC - **IHD**
Coronary artery EC - **IHF**
Coronary artery EC - **NIHF**
No enrichement
Arterial capillary EC - **IHD**
Arterial capillary EC - **IHF**
Arterial capillary EC - **NIHF**
No enrichement
Capillary EC - **IHD**
Capillary EC - **IHF**
Capillary EC - **NIHF**
Tip cells (EC) - **IHD**
Tip cells (EC) - **IHF**
No enrichement
Tip cells (EC) - **NIHF**
No enrichement

Fig. S3. VEGF signaling pathway from IPA in endothelial subtypes. Related to Figure 3.

Blue stands for downregulation or inhibition, orange/red for upregulation. Yellow line indicates inconsistent changes.

Coronary artery EC - **IHD**

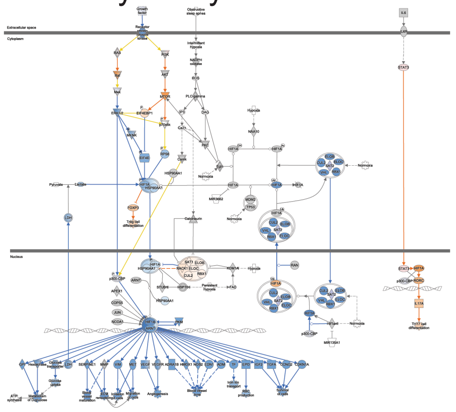

Coronary artery EC - **IHF**

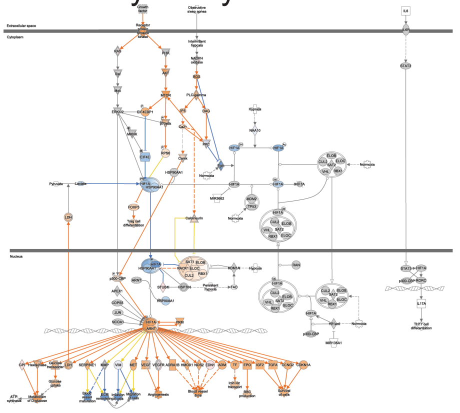

Coronary artery EC - **NIHF**

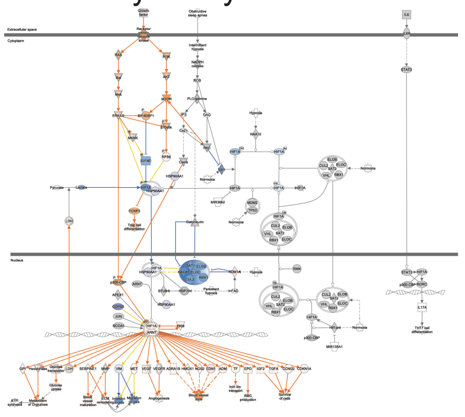

Arterial capillary EC - **IHD**

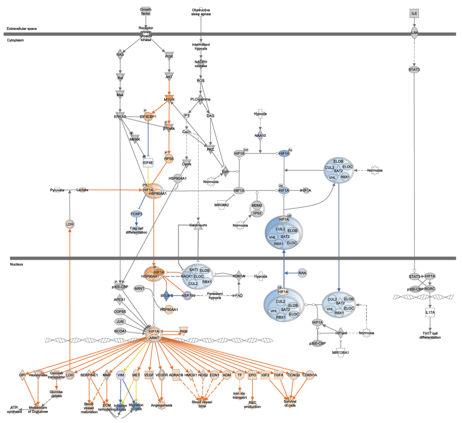

Arterial capillary EC - **IHF**

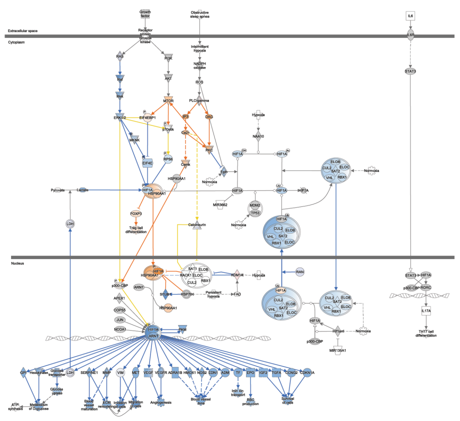

Arterial capillary EC - **NIHF**

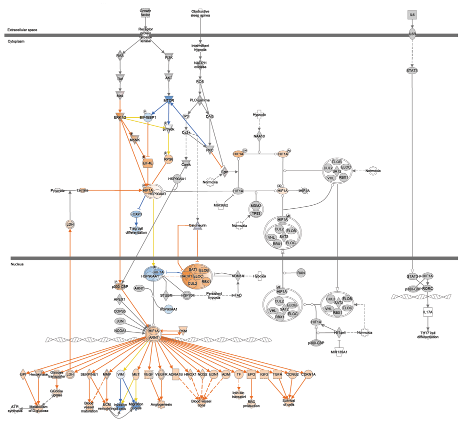

Capillary EC - **IHD**

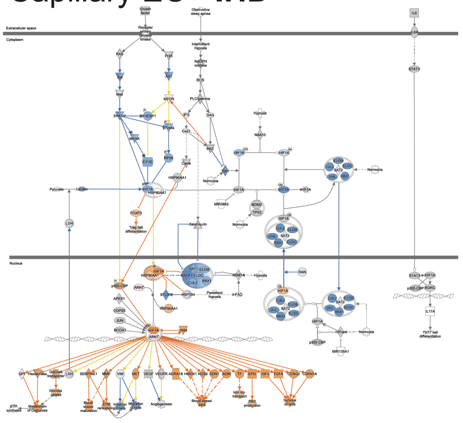

Capillary EC - **IHF**

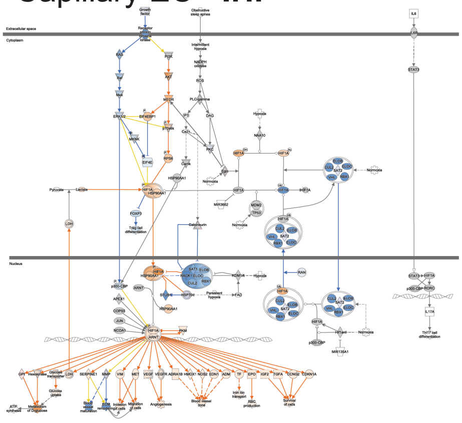

Capillary EC - **NIHF**

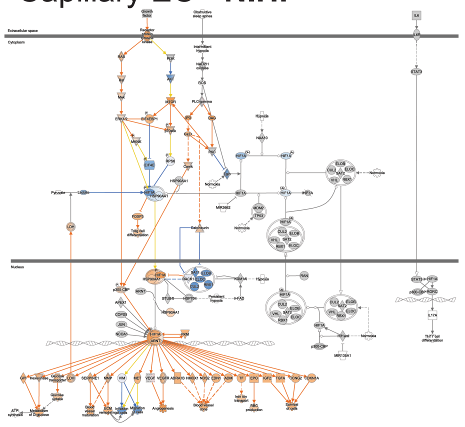

Tip cells (EC) - **IHD**

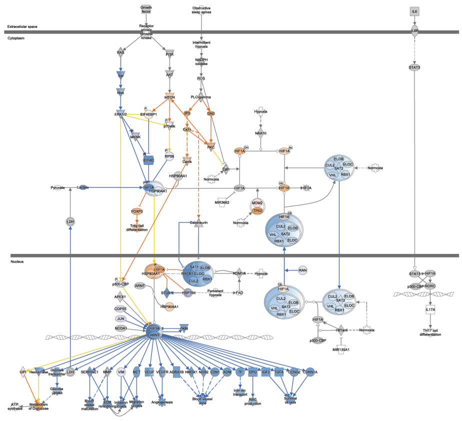

Tip cells (EC) - **IHF**

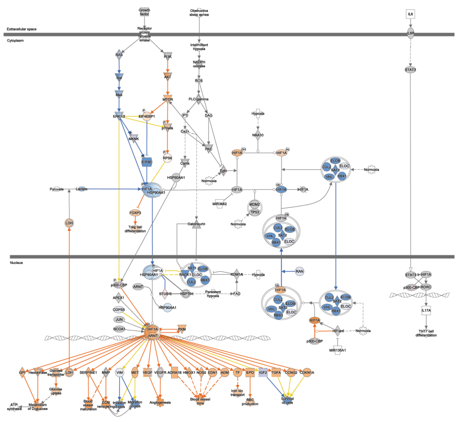

Tip cells (EC) - **NIHF**

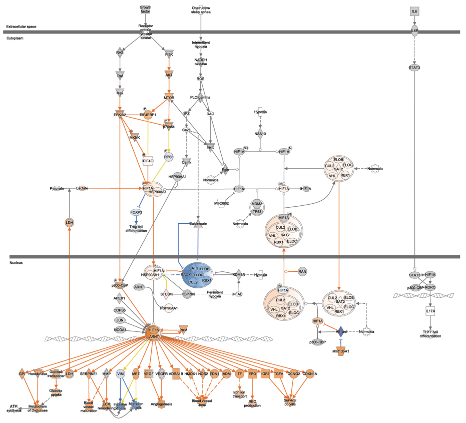

Fig. S4. HIF signaling pathway from IPA in endothelial subtypes in IHD, IHF, and NIHF. Related to Figure 3.

Blue stands for downregulation or inhibition, orange/red for upregulation. Yellow line indicates inconsistent changes.

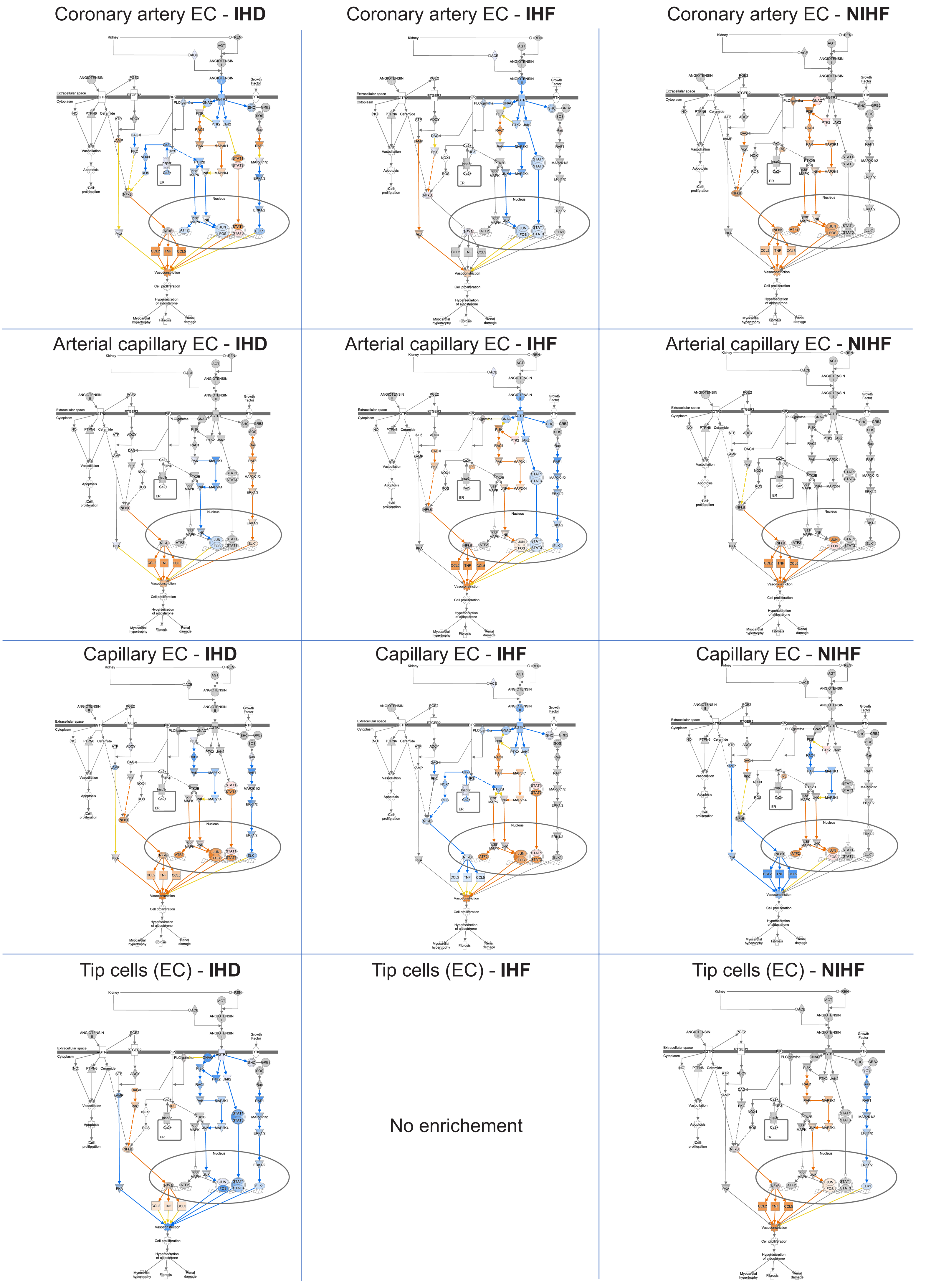

Coronary artery EC - IHD
Coronary artery EC - IHF
Coronary artery EC - NIHF
Arterial capillary EC - IHD
Arterial capillary EC - IHF
Arterial capillary EC - NIHF
Capillary EC - IHD
Capillary EC - IHF
Capillary EC - NIHF
Tip cells (EC) - IHD
Tip cells (EC) - IHF
No enrichement
Tip cells (EC) - NIHF

Fig. S5. Renin signaling pathway from IPA in endothelial subtypes in IHD, IHF, and NIHF. Related to Figure 3.

Blue stands for downregulation or inhibition, orange/red for upregulation. Yellow line indicates inconsistent changes.

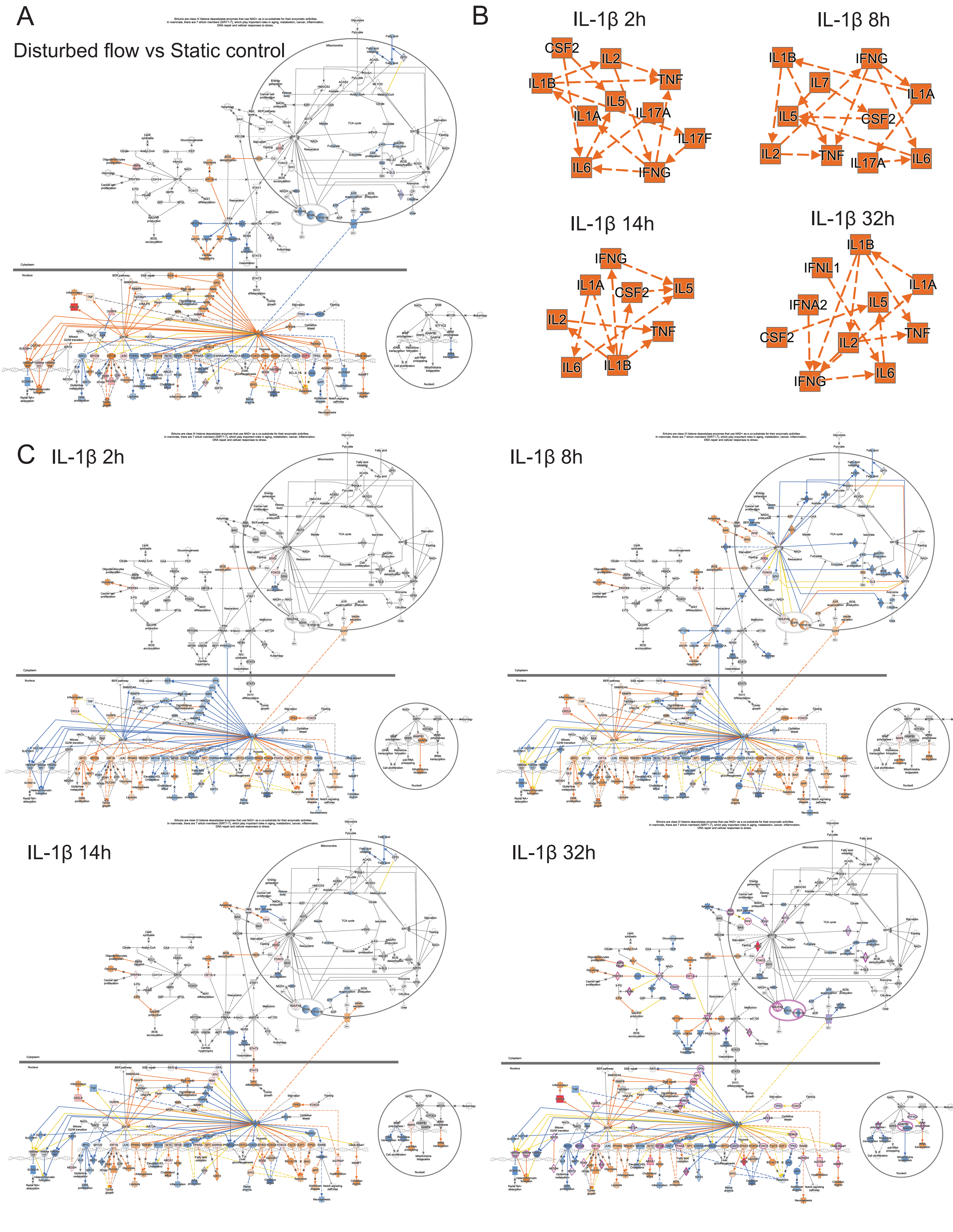
A
Disturbed flow vs Static control
B
IL-1β 2h
CSF2
IL2
TNF
IL1B
IL5
IL1A
IL17A
IL17F
IL6
IFNG
IL-1β 8h
IL1B
IFNG
IL7
IL1A
IL5
CSF2
IL2
TNF
IL17A
IL6
IL-1β 14h
IFNG
IL1A
CSF2
IL5
IL2
TNF
IL6
IL1B
IL-1β 32h
IL1B
IFNL1
IL1A
IFNA2
IL5
CSF2
TNF
IL2
IFNG
IL6
C
IL-1β 2h
IL-1β 8h
IL-1β 14h
IL-1β 32h

Fig. S6. Endothelial cell response to disturbed flow and IL-1b stimulation. Related to Figure 3.

A. Sirtuin signaling pathway from IPA in HUVEC cells under atheroprone (disturbed) flow versus static cells (*N*=3). Blue stands for downregulation or inhibition, orange/red for upregulation. Yellow line indicates inconsistent changes. **B.** IL-1b network from IPA in stimulated HAEC cells. The cells were treated for 2, 8, 14, or 32h with IL-1b. The resulting RNA-seq data were analyzed with IPA (Qiagen) to construct the upstream regulator networks from the graphical summaries. **C.** Sirtuin signaling pathway from IPA in IL-1b-treated HAECs after 2, 8, 14, and 32h of treatment. Blue stands for downregulation or inhibition, orange/red for upregulation. Yellow line indicates inconsistent changes and purple highlights measured significant changes in the dataset. All signaling pathways were constructed with IPA (Qiagen) from either core or comparison analysis.

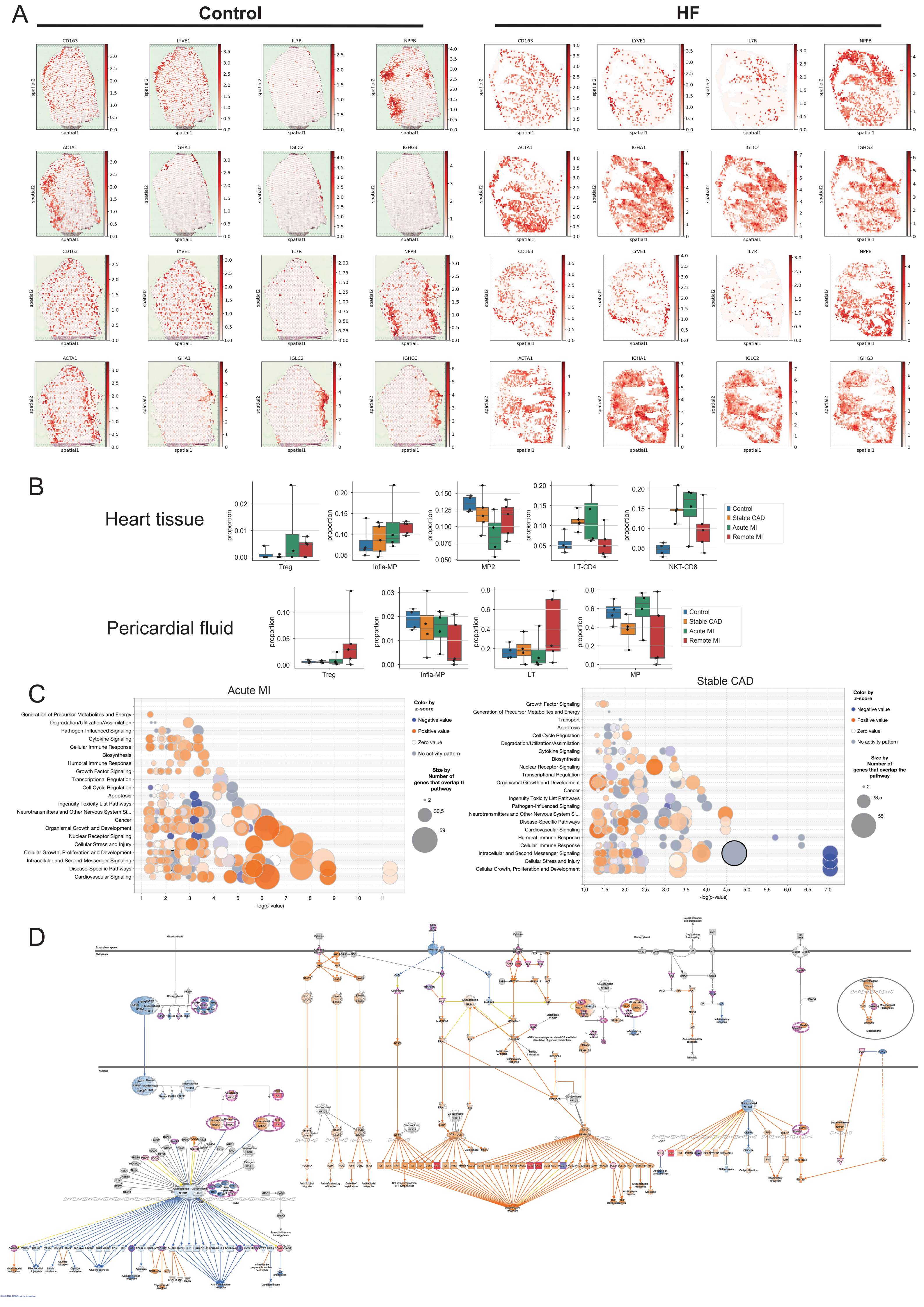
A
Control
HF
CD163
LYVE1
IL7R
NPPB
ACTA1
IGHA1
IGLC2
IGHG3
spatial1
spatial2
B
Heart tissue
Pericardial fluid
proportion
Treg
Infla-MP
MP2
LT-CD4
NKT-CD8
LT
MP
Control
Stable CAD
Acute MI
Remote MI
C
Acute MI
Stable CAD
Generation of Precursor Metabolites and Energy
Degradation/Utilization/Assimilation
Pathogen-Influenced Signaling
Cytokine Signaling
Cellular Immune Response
Biosynthesis
Humoral Immune Response
Growth Factor Signaling
Transcriptional Regulation
Cell Cycle Regulation
Apoptosis
Ingenuity Toxicity List Pathways
Neurotransmitters and Other Nervous System Si...
Cancer
Organismal Growth and Development
Nuclear Receptor Signaling
Cellular Stress and Injury
Cellular Growth, Proliferation and Development
Intracellular and Second Messenger Signaling
Disease-Specific Pathways
Cardiovascular Signaling
Transport
Color by z-score
Negative value
Positive value
Zero value
No activity pattern
Size by Number of genes that overlap th pathway
Size by Number of genes that overlap the pathway
-log(p-value)
D
Extracellular space
Cytoplasm
Nucleus
Inflammatory response
Mitochondria

Fig. S7. Immune cell proportion changes and transcriptional response. Related to Figures 4 and 5.

A. Expression of *CD163*, *LYVE1*, *IL7R* on Visium slides, from a patient with heart failure and a control sample (*n*=4). **B.** Proportion of selected immune cells across conditions in heart tissue and pericardial fluid. Whiskers show the maximum and minimum values, except for outliers (more than 1.5 times the interquartile). **C.** Enrichment of general terms and pathway sharing between terms in macrophages (MP) in stable CAD and acute MI from IPA. A pathway shared between the terms is shown as a circle of the same size and color on the same vertical line between the terms. **D.** Glucocorticoid signaling pathway from IPA in MPs in stable CAD compared to control. Blue stands for downregulation or inhibition, orange/red for upregulation. Yellow line indicates inconsistent changes and purple highlights measured significant changes in the dataset.

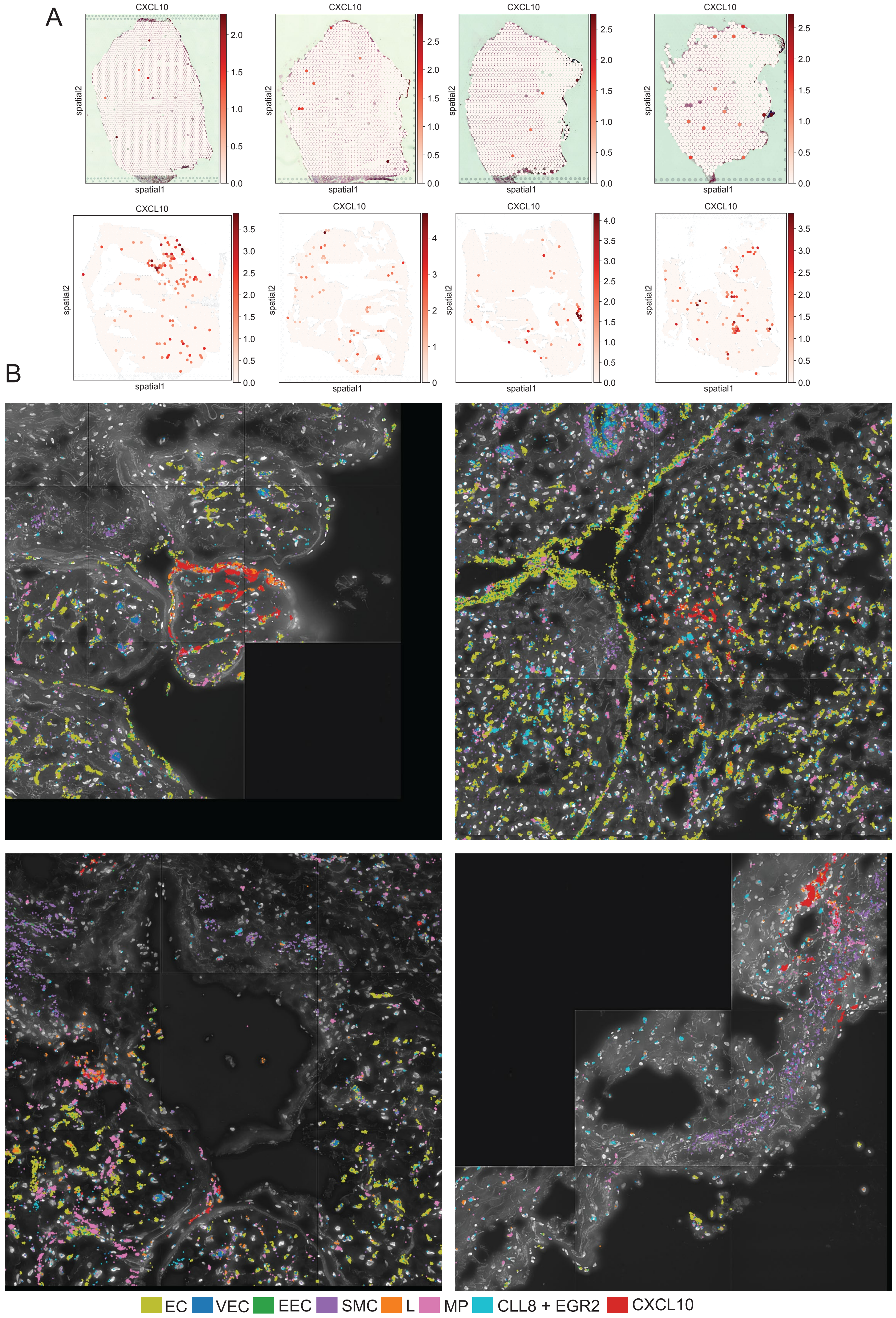
A
CXCL10
spatial2
spatial1
B
EC
VEC
EEC
SMC
L
MP
CLL8 + EGR2
CXCL10

Fig. S8. Spatial distribution of CXCL10 expression. Related to Figure 6.

A. Expression of *CXCL10* on Visium slides, from a patient with heart failure and a control sample (*n*=4). **B.** Spatial images (Resolve Molecular Cartography) for EC (*EMCN, ERG, PECAM1, CDH5, VWF*), VEC (*DKK2, ENPP2, PCSK5, CYYR1*), EEC (*PCDH7*), SMC (*NTRK3, MRVII*), L (*BCL11B, CD247, SKAP1, THEMIS*), MP (*CD163, MRC1, F13A1, MS4A6A*), inflammatory (*CCL8* and *EGR2*), and *CXCL10* gene expression in IHD (upper panels and down left) and control (down right) samples.

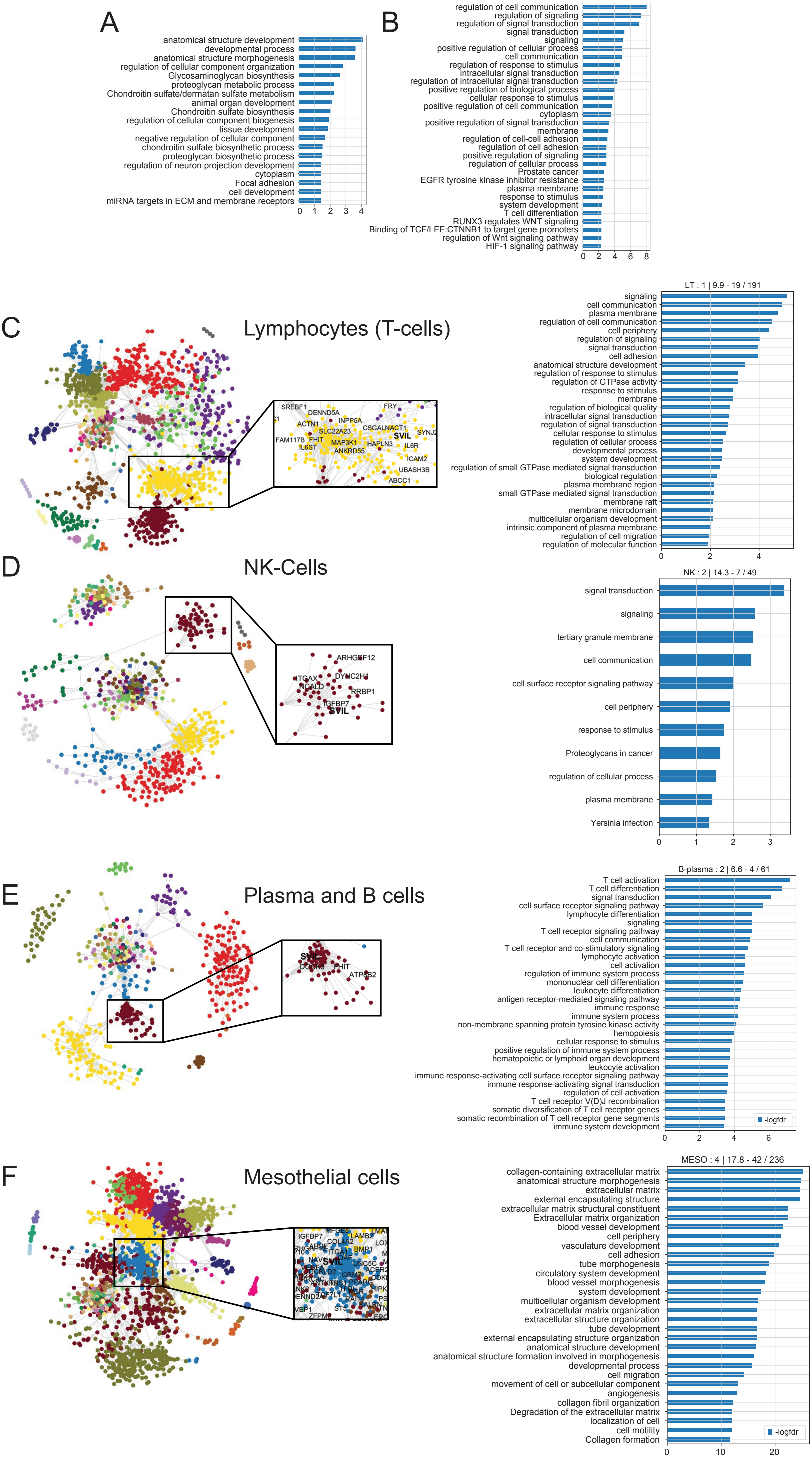

A
anatomical structure development
developmental process
anatomical structure morphogenesis
regulation of cellular component organization
Glycosaminoglycan biosynthesis
proteoglycan metabolic process
Chondroitin sulfate/dermatan sulfate metabolism
animal organ development
Chondroitin sulfate biosynthesis
regulation of cellular component biogenesis
tissue development
negative regulation of cellular component
chondroitin sulfate biosynthetic process
proteoglycan biosynthetic process
regulation of neuron projection development
cytoplasm
Focal adhesion
cell development
miRNA targets in ECM and membrane receptors
0 1 2 3 4
B
regulation of cell communication
regulation of signaling
regulation of signal transduction
signal transduction
signaling
positive regulation of cellular process
cell communication
regulation of response to stimulus
intracellular signal transduction
regulation of intracellular signal transduction
positive regulation of biological process
cellular response to stimulus
positive regulation of cell communication
cytoplasm
positive regulation of signal transduction
membrane
regulation of cell-cell adhesion
regulation of cell adhesion
positive regulation of signaling
regulation of cellular process
Prostate cancer
EGFR tyrosine kinase inhibitor resistance
plasma membrane
response to stimulus
system development
T cell differentiation
RUNX3 regulates WNT signaling
Binding of TCF/LEF:CTNNB1 to target gene promoters
regulation of Wnt signaling pathway
HIF-1 signaling pathway
0 2 4 6 8
C
Lymphocytes (T-cells)
SREBF1
DENND5A
FRY
INPP5A
ACTN1
SLC22A23
CSGALNACT1
SYNJ2
SVIL
FAM117B
FHIT
IL6ST
MAP3K1
HAPLN3
IL6R
ANKRD55
ICAM2
UBASH3B
ABCC1
LT : 1 | 9.9 - 19 / 191
signaling
cell communication
plasma membrane
regulation of cell communication
cell periphery
regulation of signaling
signal transduction
cell adhesion
anatomical structure development
regulation of response to stimulus
regulation of GTPase activity
response to stimulus
membrane
regulation of biological quality
intracellular signal transduction
regulation of signal transduction
cellular response to stimulus
regulation of cellular process
developmental process
system development
regulation of small GTPase mediated signal transduction
biological regulation
plasma membrane region
small GTPase mediated signal transduction
membrane raft
membrane microdomain
multicellular organism development
intrinsic component of plasma membrane
regulation of cell migration
regulation of molecular function
0 2 4
D
NK-Cells
ARHGEF12
ITGAX
DYNC2H1
RRBP1
IGFBP7
SVIL
NK : 2 | 14.3 - 7 / 49
signal transduction
signaling
tertiary granule membrane
cell communication
cell surface receptor signaling pathway
cell periphery
response to stimulus
Proteoglycans in cancer
regulation of cellular process
plasma membrane
Yersinia infection
0 1 2 3
E
Plasma and B cells
SVIL
FHIT
ATP8B2
B-plasma : 2 | 6.6 - 4 / 61
T cell activation
T cell differentiation
signal transduction
cell surface receptor signaling pathway
lymphocyte differentiation
signaling
T cell receptor signaling pathway
cell communication
T cell receptor and co-stimulatory signaling
lymphocyte activation
cell activation
regulation of immune system process
mononuclear cell differentiation
leukocyte differentiation
antigen receptor-mediated signaling pathway
immune response
immune system process
non-membrane spanning protein tyrosine kinase activity
hemopoiesis
cellular response to stimulus
positive regulation of immune system process
hematopoietic or lymphoid organ development
leukocyte activation
immune response-activating cell surface receptor signaling pathway
immune response-activating signal transduction
regulation of cell activation
T cell receptor V(D)J recombination
somatic diversification of T cell receptor genes
somatic recombination of T cell receptor gene segments
immune system development
-logfdr
0 2 4 6
F
Mesothelial cells
IGFBP7
LAMB2
SVIL
BMP1
UNC5C
MESO : 4 | 17.8 - 42 / 236
collagen-containing extracellular matrix
anatomical structure morphogenesis
extracellular matrix
external encapsulating structure
extracellular matrix structural constituent
Extracellular matrix organization
blood vessel development
cell periphery
vasculature development
cell adhesion
tube morphogenesis
circulatory system development
blood vessel morphogenesis
system development
multicellular organism development
extracellular matrix organization
extracellular structure organization
tube development
external encapsulating structure organization
anatomical structure development
anatomical structure formation involved in morphogenesis
developmental process
cell migration
movement of cell or subcellular component
angiogenesis
collagen fibril organization
Degradation of the extracellular matrix
localization of cell
cell motility
Collagen formation
-logfdr
0 10 20

Fig. S9. Co-expression modules of interest and associated gene-set enrichments. Related to Figure 7.

A. Gene set enrichment with Enrichr for the gene-gene co-expression module containing *SVIL* in SMC. **B.** Gene set enrichment with Enrichr of the gene-gene co-expression module containing *SVIL* in pericardial fluid cells. **C-F.** Gene-gene co-expression modules within Pericardial fluid T lymphocytes, NK cells, B and plasma cells and Mesothelial cells, in addition to a zoom in view of *SVIL*-containing modules, with their associated gene set enrichments from Enrichr.

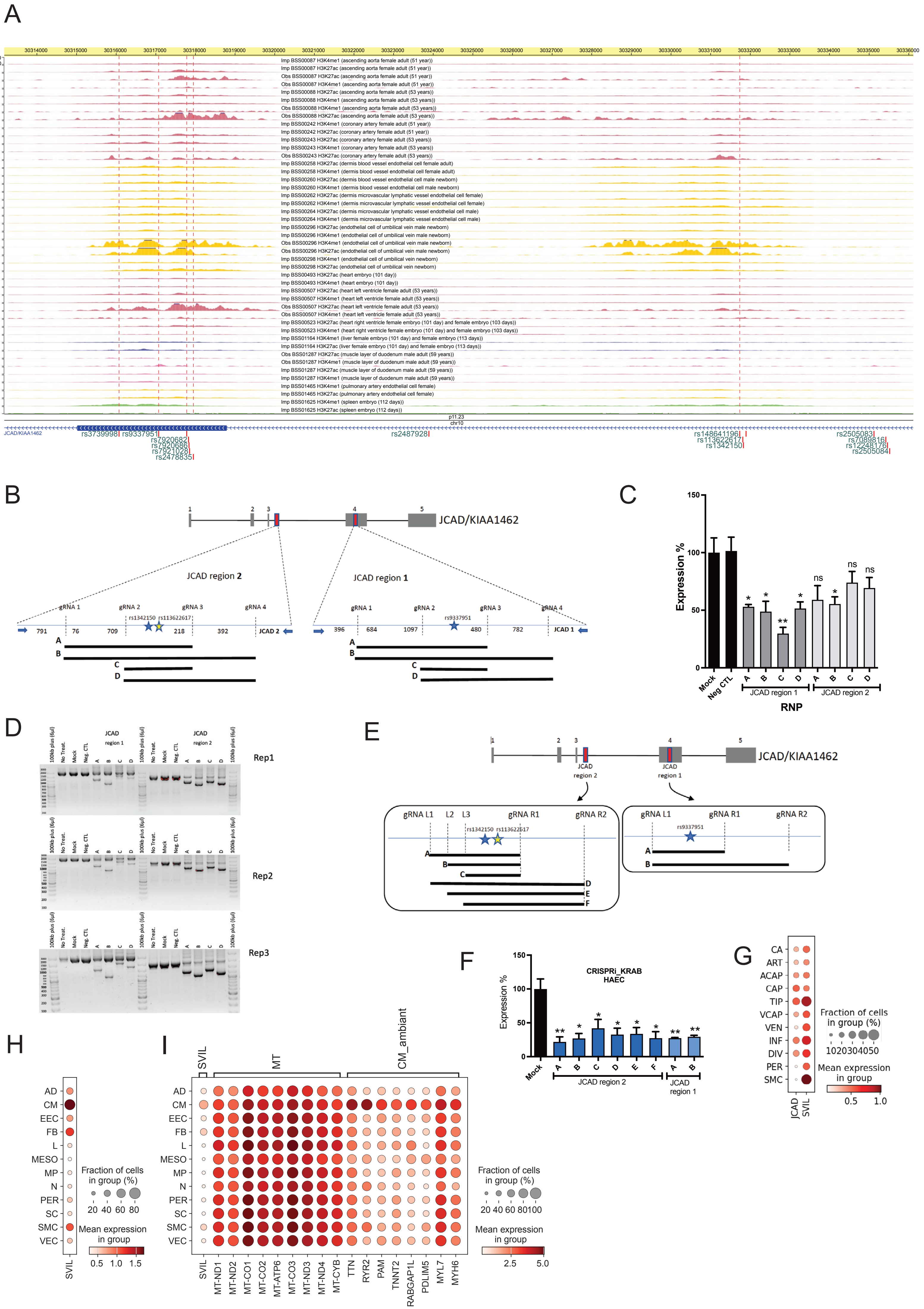
A
Imp BSS00087 H3K4me1 (ascending aorta female adult (51 year))
Imp BSS00087 H3K27ac (ascending aorta female adult (51 year))
Obs BSS00087 H3K27ac (ascending aorta female adult (51 year))
Obs BSS00087 H3K4me1 (ascending aorta female adult (51 year))
Imp BSS00088 H3K27ac (ascending aorta female adult (53 years))
Imp BSS00088 H3K4me1 (ascending aorta female adult (53 years))
Obs BSS00088 H3K4me1 (ascending aorta female adult (53 years))
Obs BSS00088 H3K27ac (ascending aorta female adult (53 years))
Imp BSS00242 H3K4me1 (coronary artery female adult (51 year))
Imp BSS00242 H3K27ac (coronary artery female adult (51 year))
Imp BSS00243 H3K27ac (coronary artery female adult (53 years))
Imp BSS00243 H3K4me1 (coronary artery female adult (53 years))
Obs BSS00243 H3K27ac (coronary artery female adult (53 years))
Imp BSS00258 H3K27ac (dermis blood vessel endothelial cell female adult)
Imp BSS00258 H3K4me1 (dermis blood vessel endothelial cell female adult)
Imp BSS00260 H3K27ac (dermis blood vessel endothelial cell male newborn)
Imp BSS00260 H3K4me1 (dermis blood vessel endothelial cell male newborn)
Imp BSS00262 H3K27ac (dermis microvascular lymphatic vessel endothelial cell female)
Imp BSS00262 H3K4me1 (dermis microvascular lymphatic vessel endothelial cell female)
Imp BSS00264 H3K27ac (dermis microvascular lymphatic vessel endothelial cell male)
Imp BSS00264 H3K4me1 (dermis microvascular lymphatic vessel endothelial cell male)
Imp BSS00296 H3K27ac (endothelial cell of umbilical vein male newborn)
Imp BSS00296 H3K4me1 (endothelial cell of umbilical vein male newborn)
Obs BSS00296 H3K4me1 (endothelial cell of umbilical vein male newborn)
Obs BSS00296 H3K27ac (endothelial cell of umbilical vein male newborn)
Imp BSS00298 H3K4me1 (endothelial cell of umbilical vein newborn)
Imp BSS00298 H3K27ac (endothelial cell of umbilical vein newborn)
Imp BSS00493 H3K27ac (heart embryo (101 day))
Imp BSS00493 H3K4me1 (heart embryo (101 day))
Imp BSS00507 H3K27ac (heart left ventricle female adult (53 years))
Imp BSS00507 H3K4me1 (heart left ventricle female adult (53 years))
Obs BSS00507 H3K27ac (heart left ventricle female adult (53 years))
Obs BSS00507 H3K4me1 (heart left ventricle female adult (53 years))
Imp BSS00523 H3K27ac (heart right ventricle female embryo (101 day) and female embryo (103 days))
Imp BSS00523 H3K4me1 (heart right ventricle female embryo (101 day) and female embryo (103 days))
Imp BSS01164 H3K4me1 (liver female embryo (101 day) and female embryo (113 days))
Imp BSS01164 H3K27ac (liver female embryo (101 day) and female embryo (113 days))
Obs BSS01287 H3K27ac (muscle layer of duodenum male adult (59 years))
Obs BSS01287 H3K4me1 (muscle layer of duodenum male adult (59 years))
Imp BSS01287 H3K27ac (muscle layer of duodenum male adult (59 years))
Imp BSS01287 H3K4me1 (muscle layer of duodenum male adult (59 years))
Imp BSS01465 H3K4me1 (pulmonary artery endothelial cell female)
Imp BSS01465 H3K27ac (pulmonary artery endothelial cell female)
Imp BSS01625 H3K4me1 (spleen embryo (112 days))
Imp BSS01625 H3K27ac (spleen embryo (112 days))
p11.23
chr10
JCAD/KIAA1462
rs3739998 rs9337951
rs7920682 rs7920686 rs7921028 rs2478835
rs2487928
rs148641196 rs113622617 rs1342150
rs2505083 rs7089816 rs12248176 rs2505084
B
JCAD/KIAA1462
JCAD region 2
JCAD region 1
gRNA 1 gRNA 2 gRNA 3 gRNA 4
rs1342150 rs113622617
791 76 709 218 392 JCAD 2
rs9337951
396 684 1097 480 782 JCAD 1
A B C D
C
Expression %
Mock Neg CTL A B C D A B C D
JCAD region 1 JCAD region 2
RNP
* * ** * ns * ns ns
D
100kb plus (6µl) No Treat. Mock Neg. CTL A B C D
JCAD region 1
JCAD region 2
Rep1
Rep2
Rep3
E
JCAD/KIAA1462
JCAD region 2
JCAD region 1
gRNA L1 L2 L3 gRNA R1 gRNA R2
rs1342150 rs113622617
A B C D E F
gRNA L1 gRNA R1 gRNA R2
rs9337951
A B
F
CRISPRi_KRAB HAEC
Expression %
Mock A B C D E F A B
JCAD region 2
JCAD region 1
G
CA ART ACAP CAP TIP VCAP VEN INF DIV PER SMC
JCAD SVIL
Fraction of cells in group (%)
10 20 30 40 50
Mean expression in group
0.5 1.0
H
AD CM EEC FB L MESO MP N PER SC SMC VEC
SVIL
Fraction of cells in group (%)
20 40 60 80
Mean expression in group
0.5 1.0 1.5
I
SVIL MT CM_ambiant
AD CM EEC FB L MESO MP N PER SC SMC VEC
SVIL MT-ND1 MT-ND2 MT-CO1 MT-CO2 MT-ATP6 MT-CO3 MT-ND3 MT-ND4 MT-CYB TTN RYR2 PAM TNNT2 RABGAP1L PDLIM5 MYL7 MYH6
Fraction of cells in group (%)
20 40 60 80 100
Mean expression in group
2.5 5.0

Fig. S10. Dissection of JCAD / SVIL locus genetic variants, and JCAD/SVIL expression distribution in heart tissue. Related to Figure 7.

A. Browser shot of the rs9337951 locus on chromosome 10 with histone marker signals for aorta, endothelial cells, heart, liver, muscle, and spleen. **B**. Schematic describing the regions targeted for CRISPR-mediated deletion using different combinations of two guide RNAs (gRNAs within the *JCAD*/*SVIL* locus. **C.** The effect of CRISPR deletion on *JCAD* gene expression. **D.** Confirmation of the success and efficiency of deletion using PCR. **E.** Schematic describing the regions targeted for CRISPR-mediated inhibition using different combinations of two gRNAs **F.** The effect of CRISPR inhibition on *JCAD* gene expression. **G.** *JCAD* and *SVIL* expression in tissue cells by snRNA-seq. **H**. *SVIL* expression in main cell types in snRNA-seq data. **I**. *SVIL* expression together with mitochondrial (MT) and typical ambient RNA genes in the snRNA-seq data.

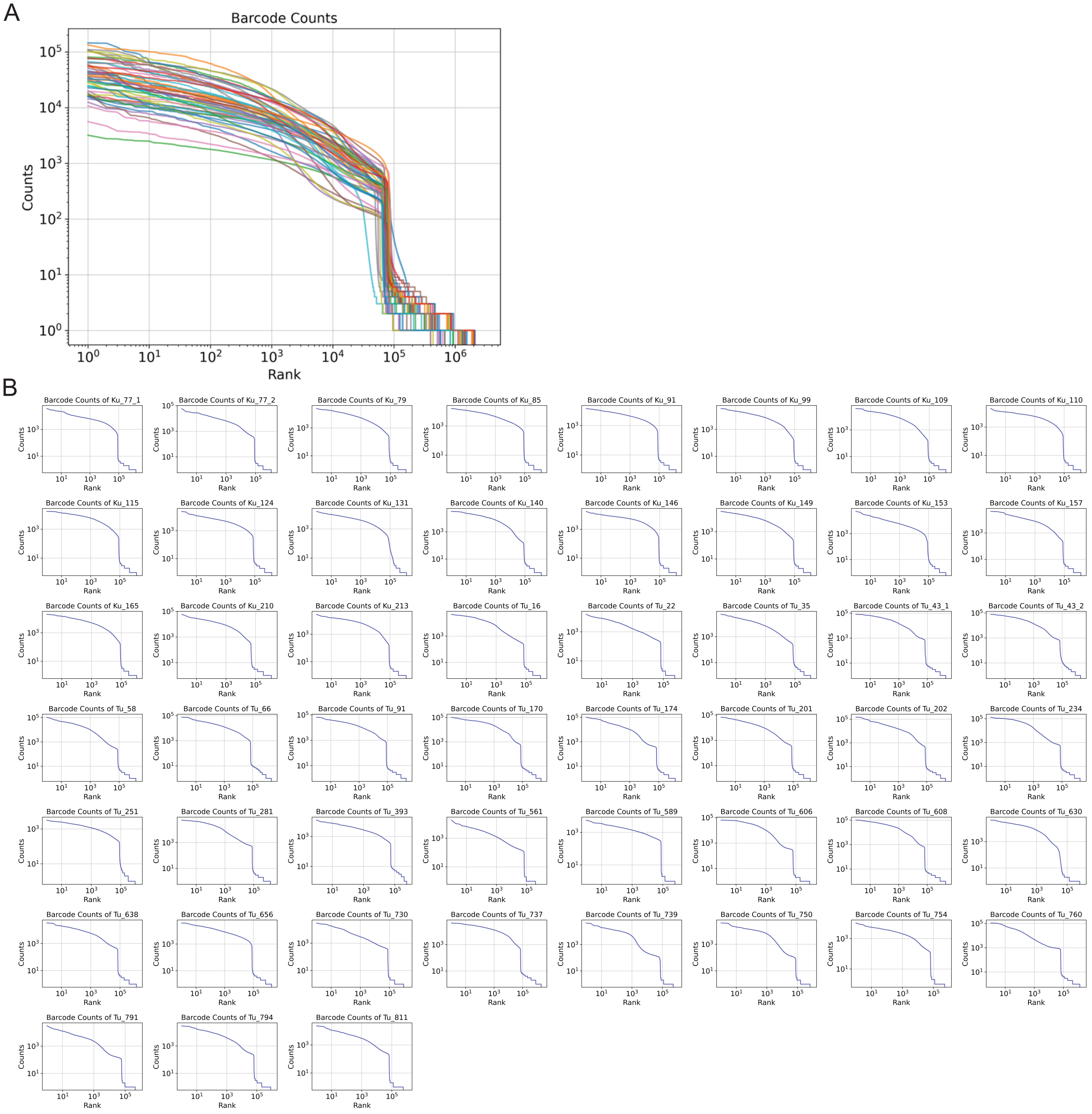
A
Barcode Counts
Counts
Rank
B
Barcode Counts of Ku_77_1
Barcode Counts of Ku_77_2
Barcode Counts of Ku_79
Barcode Counts of Ku_85
Barcode Counts of Ku_91
Barcode Counts of Ku_99
Barcode Counts of Ku_109
Barcode Counts of Ku_110
Barcode Counts of Ku_115
Barcode Counts of Ku_124
Barcode Counts of Ku_131
Barcode Counts of Ku_140
Barcode Counts of Ku_146
Barcode Counts of Ku_149
Barcode Counts of Ku_153
Barcode Counts of Ku_157
Barcode Counts of Ku_165
Barcode Counts of Ku_210
Barcode Counts of Ku_213
Barcode Counts of Tu_16
Barcode Counts of Tu_22
Barcode Counts of Tu_35
Barcode Counts of Tu_43_1
Barcode Counts of Tu_43_2
Barcode Counts of Tu_58
Barcode Counts of Tu_66
Barcode Counts of Tu_91
Barcode Counts of Tu_170
Barcode Counts of Tu_174
Barcode Counts of Tu_201
Barcode Counts of Tu_202
Barcode Counts of Tu_234
Barcode Counts of Tu_251
Barcode Counts of Tu_281
Barcode Counts of Tu_393
Barcode Counts of Tu_561
Barcode Counts of Tu_589
Barcode Counts of Tu_606
Barcode Counts of Tu_608
Barcode Counts of Tu_630
Barcode Counts of Tu_638
Barcode Counts of Tu_656
Barcode Counts of Tu_730
Barcode Counts of Tu_737
Barcode Counts of Tu_739
Barcode Counts of Tu_750
Barcode Counts of Tu_754
Barcode Counts of Tu_760
Barcode Counts of Tu_791
Barcode Counts of Tu_794
Barcode Counts of Tu_811

Fig. S11. Heart snRNA-seq barcode rank plots. Related to STAR Methods.

A-B. Heart snRNA-seq barcode rank Plots. **A**: Combined plot for all samples. **B**: Separate plot for each sample.

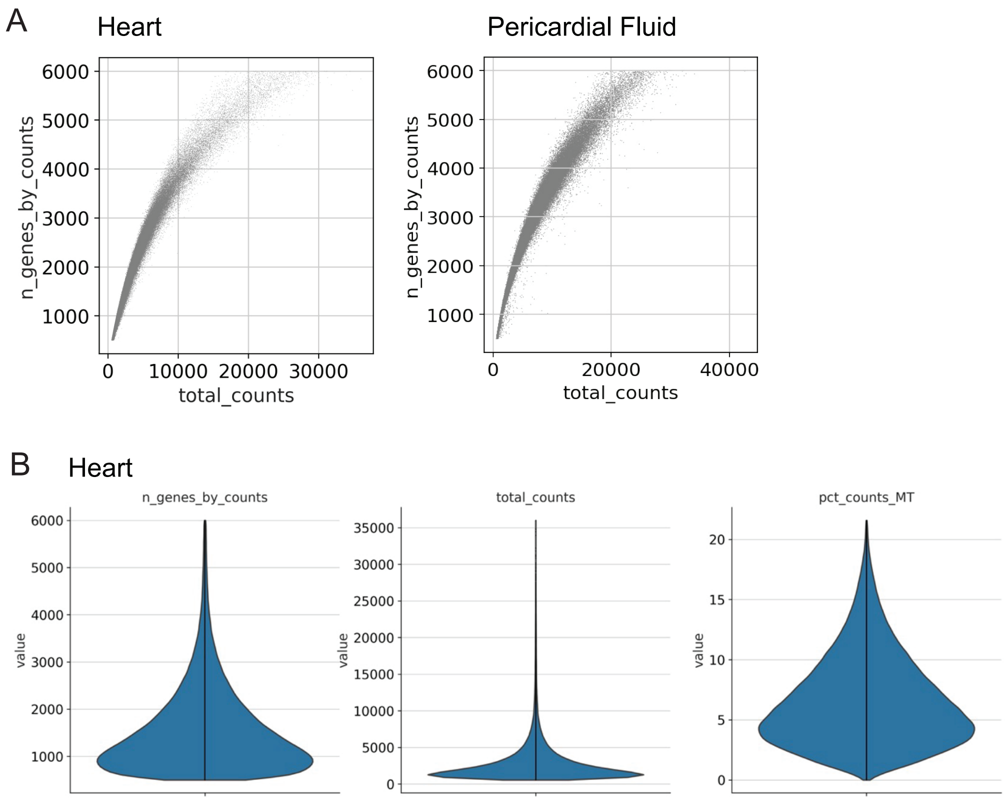

A
Heart
Pericardial Fluid
n_genes_by_counts
total_counts
6000
5000
4000
3000
2000
1000
0
10000
20000
30000
40000
B
Heart
n_genes_by_counts
total_counts
pct_counts_MT
value
35000
25000
15000
5000
20
15
10
5

Fig. S12. snRNA-seq QC metrics distribution. Related to STAR Methods.

A. Scatter plot representing the number of detected genes and the total number of counts, for each nucleus, in the heart snRNA-seq samples (left), and pericardial fluid snRNA-seq samples (right). **B.** QC metric distribution across nuclei for heart snRNA-seq samples, after filtering. From left to right: number of detected genes, total number of counts, mitochondrial expression percentage.

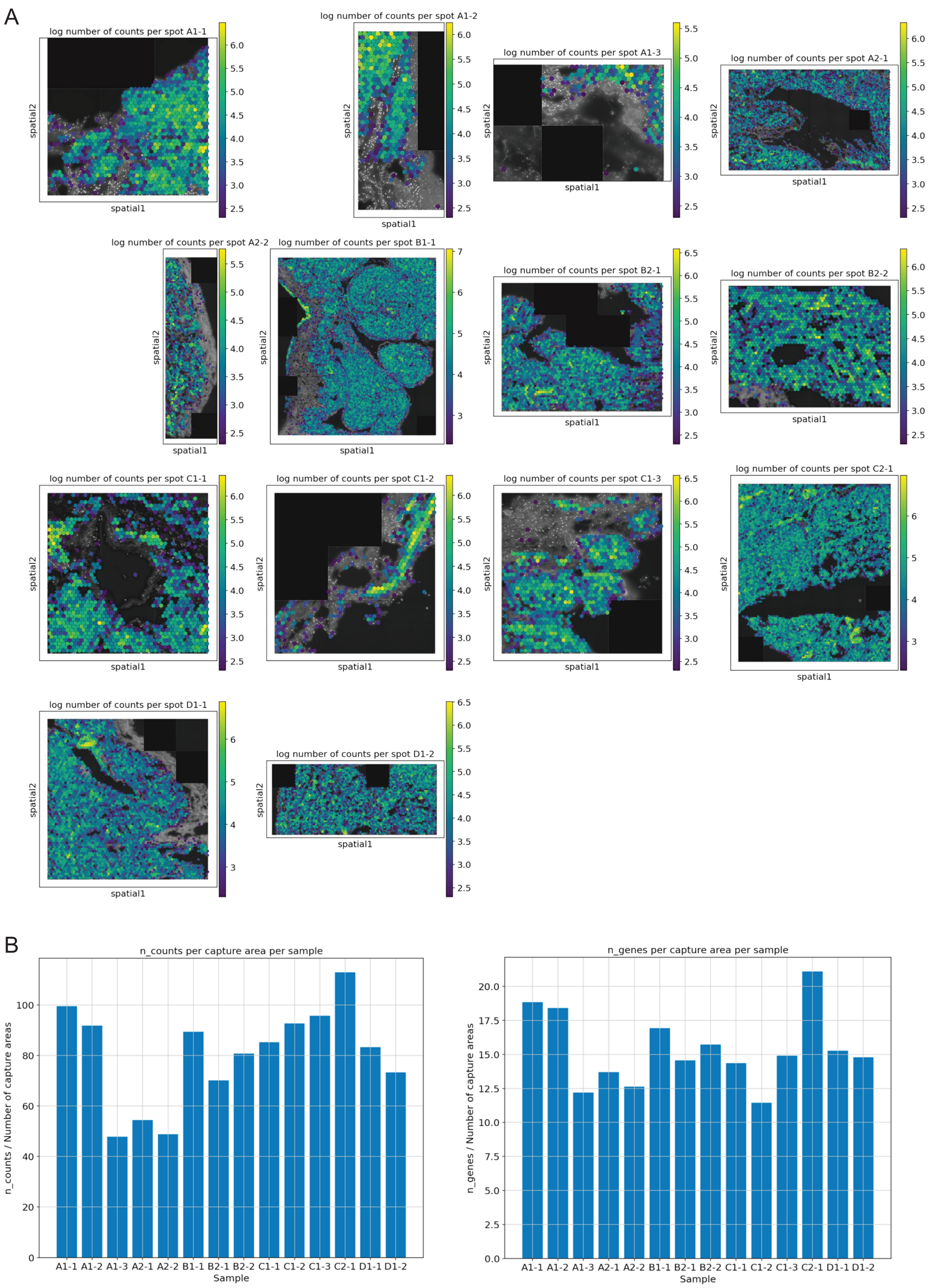
A
log number of counts per spot A1-1
log number of counts per spot A1-2
log number of counts per spot A1-3
log number of counts per spot A2-1
log number of counts per spot A2-2
log number of counts per spot B1-1
log number of counts per spot B2-1
log number of counts per spot B2-2
log number of counts per spot C1-1
log number of counts per spot C1-2
log number of counts per spot C1-3
log number of counts per spot C2-1
log number of counts per spot D1-1
log number of counts per spot D1-2
spatial1
spatial2
B
n_counts per capture area per sample
n_counts / Number of capture areas
n_genes per capture area per sample
n_genes / Number of capture areas
Sample
A1-1 A1-2 A1-3 A2-1 A2-2 B1-1 B2-1 B2-2 C1-1 C1-2 C1-3 C2-1 D1-1 D1-2

Fig. S13. Resolve spatial transcriptomics QC metrics distribution. Related to STAR Methods.

A. Distribution of number per transcript, for each Resolve spatial transcriptomics slide, in log scale. **B.** Average number of counts (left) and detected genes (right) per spot, for each Resolve spatial transcriptomics.

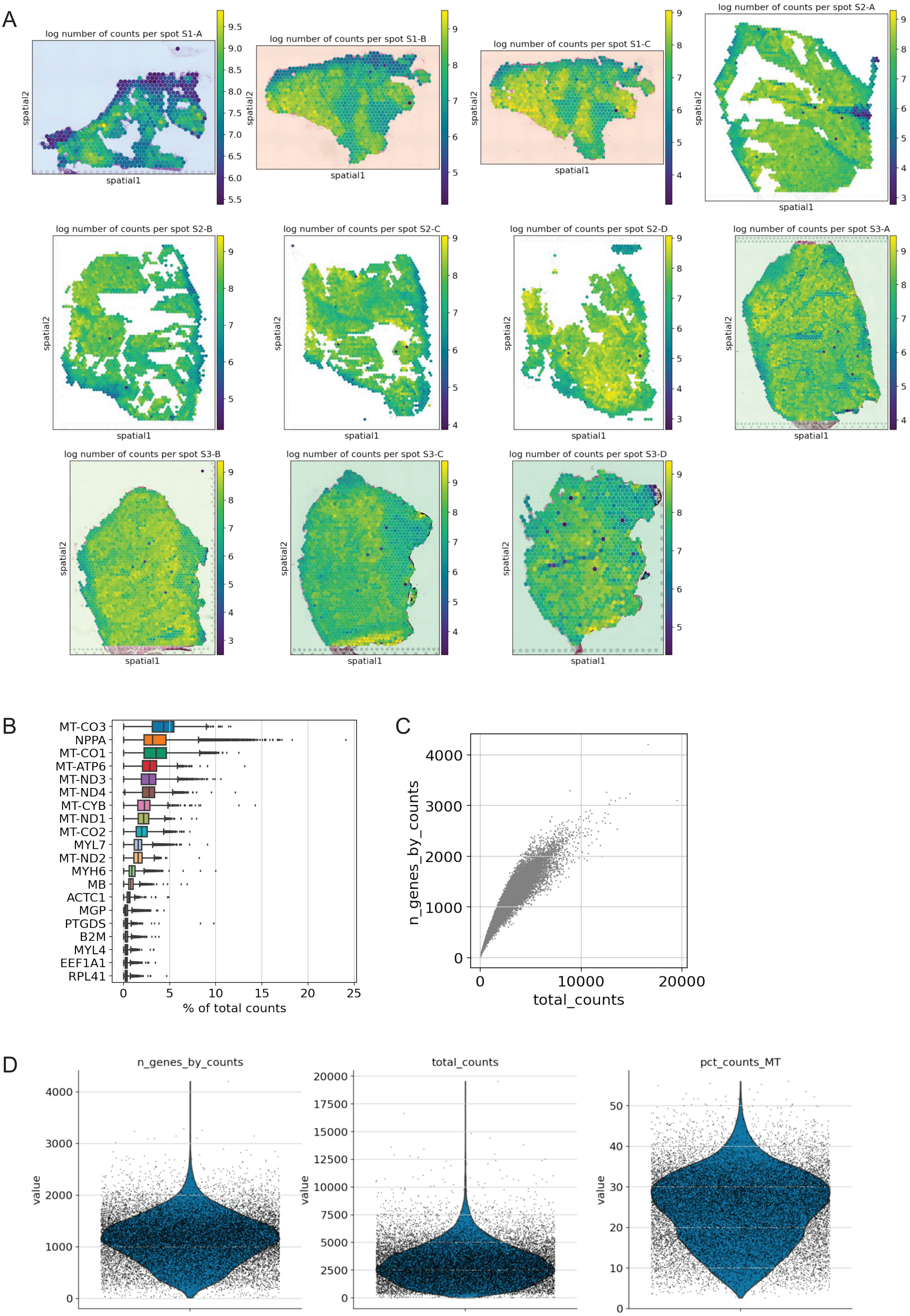
A
log number of counts per spot S1-A
log number of counts per spot S1-B
log number of counts per spot S1-C
log number of counts per spot S2-A
log number of counts per spot S2-B
log number of counts per spot S2-C
log number of counts per spot S2-D
log number of counts per spot S3-A
log number of counts per spot S3-B
log number of counts per spot S3-C
log number of counts per spot S3-D
spatial1
spatial2
B
MT-CO3
NPPA
MT-CO1
MT-ATP6
MT-ND3
MT-ND4
MT-CYB
MT-ND1
MT-CO2
MYL7
MT-ND2
MYH6
MB
ACTC1
MGP
PTGDS
B2M
MYL4
EEF1A1
RPL41
0 5 10 15 20 25
% of total counts
C
n_genes_by_counts
total_counts
0 10000 20000
0 1000 2000 3000 4000
D
n_genes_by_counts
total_counts
pct_counts_MT
value

Fig. S14. Visium spatial transcriptomics QC distribution. Related to STAR Methods.

A. Distribution of number per transcript, for each Visium spatial transcriptomics slide, in log scale. **B.** Expression distribution (per capture area) of the top 20 highest expressed genes, in all Visium spatial transcriptomics samples. **C.** Scatter plot representing the number of detected genes and the total number of counts, for each spot, in all Visium spatial transcriptomics samples. **D.** QC metric distribution across all spots for all Visium spatial transcriptomics samples, after filtering. From left to right: number of detected genes, total number of counts, mitochondrial expression percentage.

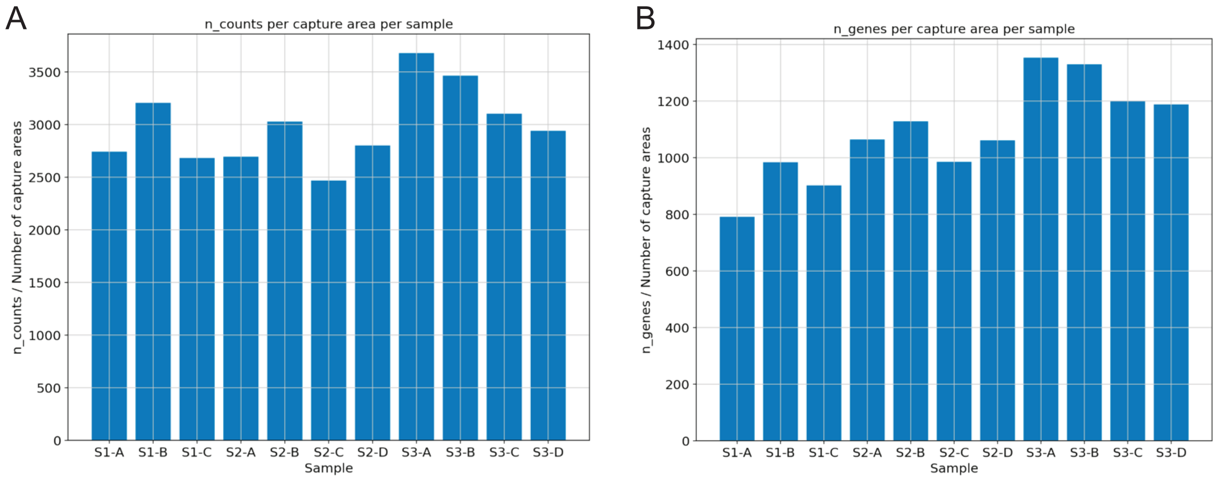
A
n_counts per capture area per sample
n_counts / Number of capture areas
0
500
1000
1500
2000
2500
3000
3500
S1-A S1-B S1-C S2-A S2-B S2-C S2-D S3-A S3-B S3-C S3-D
Sample
B
n_genes per capture area per sample
n_genes / Number of capture areas
0
200
400
600
800
1000
1200
1400
S1-A S1-B S1-C S2-A S2-B S2-C S2-D S3-A S3-B S3-C S3-D
Sample

Fig. S15. Distribution of the number of counts and genes for Visium spatial transcriptomics. Related to STAR Methods.

A-B. Average number of counts (**A**) and detected genes (**B**) per spot, for each Visium spatial transcriptomics sample.

Table S1. Results for allelic activity reporter assay. Related to Figure 7.

		teloHAEC 0h		teloHAEC 6h Il1b		teloHAEC 24h Il1b		HASMC chol		
RS Number	Position (GRCh37)	logFC	FDR	logFC	FDR	logFC	FDR	logFC	FDR	variant typ
rs2478839	chr10:30306804	0,029497	0,89317	-0,22841	0,594769	0,068412	0,895356	0,326823	0,203501	common
rs12762440	chr10:30306879	0,503904	0,12795	0,584236	0,479108	-0,21375	0,566648	-0,50645	0,101235	rare
rs9337951	chr10:30317073	-0,70934	0,0661	-0,22455	0,555735	-0,08465	0,895356	0,471101	0,201433	common
rs531337994	chr10:30317781	0,721114	0,0661	0,307885	0,488862	0,871545	0,16561	1,106221	0,004756	rare
rs7920682	chr10:30317826	-0,44092	0,17706	0,519276	0,479108	-0,27119	0,56637	-0,58955	0,115174	common
rs7920686	chr10:30317838	-0,44092	0,17706	0,519276	0,479108	-0,27119	0,56637	-0,58955	0,115174	common
rs7921028	chr10:30317853	-0,50678	0,33249	1,147921	0,479108	-0,40207	0,56637	-0,1748	0,75763	rare
rs2478835	chr10:30317949	0,256671	0,36864	-0,24893	0,549716	0,61482	0,264878	0,504799	0,101235	common
rs2487928	chr10:30323892	-0,16943	0,42384	0,312584	0,479108	-0,02049	0,912102	-0,26084	0,2621	common
rs148641196	chr10:30331736	-0,92915	0,00225	-1,3914	0,001085	0,108742	0,895356	-1,05572	0,004181	rare
rs113622617	chr10:30331813	0,383709	0,12795	0,696887	0,042102	0,774711	0,49638	0,681045	0,091074	rare
rs1342150	chr10:30331829	-0,4658	0,0661	-1,0913	0,003272	-0,68255	0,49638	-0,83159	0,004756	common
rs193042870	chr10:30331895	0,353719	0,18304	0,954916	0,041686	1,121558	0,332584	-0,4443	0,116393	rare
rs7089816	chr10:30335422	-0,16507	0,43332	-0,25323	0,488862	-0,15209	0,56637	-0,24679	0,2621	common
rs12248176	chr10:30335464	0,115829	0,58075	-0,05112	0,914561	-0,06663	0,895356	0,1731	0,423611	rare
rs2505084	chr10:30335520	0,396745	0,17706	0,02093	0,931401	0,307866	0,332584	0,114771	0,566492	common

Table S2A. CAREBANK samples characteristics. Related to STAR Methods.

	Control	IHD	IHF	NIHF
N	6	11	11	3
Gender	4M 2F	9M 2F	10M 1F	2M 1F
Age (years) *	67± 4	68 ± 8	66 ± 13	72 ± 6
BMI (kg/m2) *	29 ± 4	30 ± 4	28 ± 5	29 ± 4
Smoker	0	0	4	0
Atrial fibrillation	0	0	2	2
Treatment for hypertension	4	9	9	3
Treatment for diabetes	2	4	6	0
Treatment for Hypercholesterolemia	3	10	11	1
Heart failure	0	0	5	3
Prior Stroke / TIA	1	0	1	0
Previous myocardial infarction	0	0	4	0
Previous PCI	0	3	2	0
Previous CABG	0	0	0	0
Number of stenosis in the coronary arteries	0	2	2	0
LVEF*	64 ± 4	64 ± 7	38 ± 7	41 ± 9
Betablocker	2	10	9	3
Calcium blocker	0	3	4	2
ACE-inhibitor	5	7	10	3
Warfarin	0	0	3	0
NOAC	0	1	1	1
ASA	4	9	7	0
ADPB	0	1	1	0
Statin	3	10	11	1
Insuline	0	3	4	0

**Mean ± Standard deviation*

Table S2B. PERIHEART samples characteristics. Related to STAR Methods.

	Control	stable CAD	acute MI	remote MI
N	4	5	4	5
Gender	all male	all male	all male	all male
Age (years) *	61±7	68 ±4	66 ±6	72 ±6
BMI (kg/m2) *	26 ±4	26 ±3	25 ±2	28 ±3
Smoker	0	0	2	2
Atrial fibrillation	0	0	0	0
Treatment for hypertension	3	4	3	3
Treatment for diabetes	0	1	1	2
Treatment for Hypercholesterolemia	1	3	4	3
Heart failure	0	0	2	1
Prior Stroke / TIA	0	0	0	0
Previous myocardial infarction	0	0	4	5
Previous PCI	0	0	1	3
Previous CABG	0	0	0	0
Number of stenosis in the coronary arteries	0	3	3	3
LVEF *	75 ±4	61 ±7	49 ±13	48 ±9
Betablocker	2	2	3	5
Calcium blocker	0	0	2	0
ACE-inhibitor	1	1	2	4
Warfarin	0	0	0	2
NOAC	0	0	0	0
ASA	0	4	3	4
ADPB	0	0	1	1
Statin	1	4	3	5
Insuline	0	0	0	0

**Mean ± Standard deviation*
