# Supplementary material for: Transcriptomic and spatial dissection of human ex vivo right atrial tissue reveals proinflammatory microvascular changes in ischemic heart disease
Source: Cell Rep Med. 2024 May 21;5(5):101556. doi: 10.1016/j.xcrm.2024.101556 (PMC11148807; doi:10.1016/j.xcrm.2024.101556)
Supplement: Document S2. Article plus supplemental information [file mmc3.pdf]

Cell Reports
Medicine

Article

Transcriptomic and spatial dissection of human *ex vivo* right atrial tissue reveals proinflammatory microvascular changes in ischemic heart disease

Graphical abstract

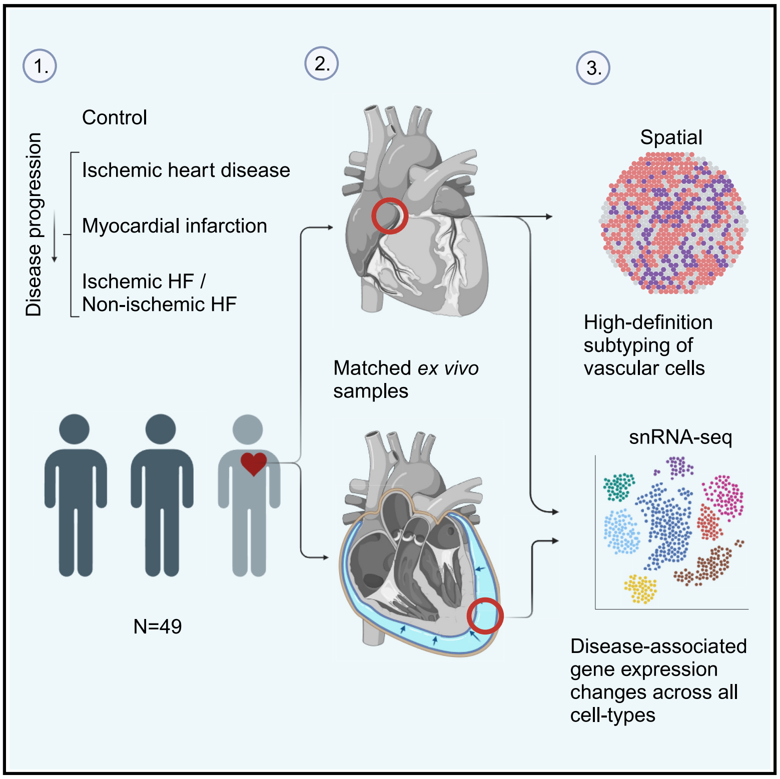

Highlights

- High-definition mapping of vascular cell subtypes in human *ex vivo* tissue
- Disease progression alters cell composition and gene expression of right atrium
- Pathway changes across cell types indicate vascular component in the progression
- Pro-inflammatory changes and lipid-associated macrophages relate to progression

Authors

Suvi Linna-Kuosmanen, Eloi Schmauch, Kyriakitsa Galani, ..., Pasi Tavi, Tuomas Kiviniemi, Manolis Kellis

Correspondence

suvi.linna-kuosmanen@uef.fi (S.L.-K.), manoli@mit.edu (M.K.)

In brief

In this paper, Linna-Kuosmanen et al. provide a high-resolution dissection of the changes occurring in human *ex vivo* right atrial tissue in response to ischemic heart disease and heart failure, revealing cellular and transcriptomic alterations promoting inflammation and vascular dysfunction along disease progression.

Linna-Kuosmanen et al., 2024, Cell Reports Medicine *5*, 101556
May 21, 2024 © 2024 The Author(s). Published by Elsevier Inc.
https://doi.org/10.1016/j.xcrm.2024.101556

CellPress

Cell Reports Medicine

CellPress
OPEN ACCESS

Article

Transcriptomic and spatial dissection of human *ex vivo* right atrial tissue reveals proinflammatory microvascular changes in ischemic heart disease

Suvi Linna-Kuosmanen,[1,2,3,13,14,*] Eloi Schmauch,[1,2,3,13] Kyriakitsa Galani,[1,2] Johannes Ojanen,[1,2,3] Carles A. Boix,[1,2] Tiit Örd,[3] Anu Toropainen,[3] Prosanta K. Singha,[3] Pierre R. Moreau,[3] Kristiina Harju,[3] Adriana Blazeski,[2,4] Åsa Segerstolpe,[2] Veikko Lahtinen,[5,6] Lei Hou,[1,2] Kai Kang,[1,2] Elamaran Meibalan,[2,4] Leandro Z. Agudelo,[1,2] Hannu Kokki,[7] Jari Halonen,[7,8] Juho Jalkanen,[6] Jarmo Gunn,[5,9] Calum A. MacRae,[2,10,11,12] Maija Hollmén,[6] Juha E.K. Hartikainen,[7,8] Minna U. Kaikkonen,[3] Guillermo García-Cardeña,[2,4,12] Pasi Tavi,[3] Tuomas Kiviniemi,[5,9,11] and Manolis Kellis[1,2,*]

[1]Computer Science and Artificial Intelligence Laboratory, Massachusetts Institute of Technology, Cambridge, MA 02139, USA
[2]Broad Institute of MIT and Harvard, Cambridge, MA 02142, USA
[3]A. I. Virtanen Institute for Molecular Sciences, University of Eastern Finland, 70211 Kuopio, Finland
[4]Center for Excellence in Vascular Biology, Department of Pathology, Brigham and Women's Hospital and Harvard Medical School, Boston, MA 02115, USA
[5]Heart Center, Turku University Hospital, 20521 Turku, Finland
[6]MediCity Research Laboratories and InFLAMES Flagship, University of Turku, 20500 Turku, Finland
[7]School of Medicine, University of Eastern Finland, 70211 Kuopio, Finland
[8]Heart Center, Kuopio University Hospital, 70200 Kuopio, Finland
[9]Department of Medicine, University of Turku, 20500 Turku, Finland
[10]Department of Medicine, Harvard Medical School, Boston, MA 02115, USA
[11]Cardiovascular Medicine and Network Medicine Division, Brigham and Women's Hospital, Harvard Medical School, Boston, MA 02115, USA
[12]Harvard Stem Cell Institute, Cambridge, MA 02138, USA
[13]These authors contributed equally
[14]Lead contact
*Correspondence: suvi.linna-kuosmanen@uef.fi (S.L.-K.), manoli@mit.edu (M.K.)
https://doi.org/10.1016/j.xcrm.2024.101556

SUMMARY

Cardiovascular disease plays a central role in the electrical and structural remodeling of the right atrium, predisposing to arrhythmias, heart failure, and sudden death. Here, we dissect with single-nuclei RNA sequencing (snRNA-seq) and spatial transcriptomics the gene expression changes in the human *ex vivo* right atrial tissue and pericardial fluid in ischemic heart disease, myocardial infarction, and ischemic and non-ischemic heart failure using asymptomatic patients with valvular disease who undergo preventive surgery as the control group. We reveal substantial differences in disease-associated gene expression in all cell types, collectively suggesting inflammatory microvascular dysfunction and changes in the right atrial tissue composition as the valvular and vascular diseases progress into heart failure. The data collectively suggest that investigation of human cardiovascular disease should expand to all functionally important parts of the heart, which may help us to identify mechanisms promoting more severe types of the disease.

INTRODUCTION

Several papers describing the cellular composition of the heart[1–4] and its transcriptional changes in cardiovascular disease[5–9] have been published. However, the effects of cardiovascular disease on the right atrium have not been investigated, despite its central role in the normal cardiac function[10] and the unknown mechanisms by which chronic cardiovascular diseases cause electrical and structural remodeling of the atrial myocardium, predisposing to unique pathologies, such as sinus node dysfunction and arrhythmias, and increasing the risk of heart failure and sudden death.[11–13]

According to recent studies, less than 20% of patients with known or suspected ischemic heart disease have obstructive disease.[14–17] Instead, coronary microvascular dysfunction has been suggested as an underlying factor that associates with more advanced disease and worse disease outcomes,[17–22] but the lack of tools for microvascular dissection has left the mechanisms unexplored in human tissue.[17] Microvascular aberrations mediate ischemia and cause symptoms both in patients with

© 2024 The Author(s). Published by Elsevier Inc.
This is an open access article under the CC BY license (http://creativecommons.org/licenses/by/4.0/).

obstructed and unobstructed coronary arteries,[23,24] and, therefore, its effects are unlikely to be limited to the left ventricle.

Here, we present a comprehensive single-cell atlas of the human *ex vivo* right atrium in valvular and ischemic heart disease, following their progression to heart failure.

RESULTS

Cells of the *ex vivo* right atrium

We obtained *ex vivo* cardiac tissue samples from the right atrial appendages of 49 patients (Figure 1A). As the samples were collected during open-heart surgeries, no age-matched healthy control samples exist. Instead, we formed our control group (altogether 10 samples) from the samples of the patients with valvular heart disease, who were at the time of collection asymptomatic, undergoing preventive surgery, and whose hearts appeared otherwise healthy. In addition, we profiled 11 patients with ischemic heart disease (IHD, i.e., patients with coronary artery disease [CAD], including patients with valvular disease), 11 patients with IHD and heart failure (ischemic heart failure [IHF], including patients with valvular disease), and three patients with non-ischemic heart failure (NIHF; i.e., patients with valvular disease and heart failure but no CAD). In addition, we had three groups for myocardial infarction (MI): five patients with stable CAD (i.e., patients who underwent elective surgery; no MI), four patients hospitalized and operated due to MI (acute MI), and five patients who had suffered MI earlier in life (remote MI). We profiled the samples with single-nuclei RNA sequencing (snRNA-seq) and processed the data using QClus,[25] resulting in 296,682 nuclei. These nuclei were grouped into 12 main cell types (Figures 1B–1F), using previously published marker genes[1–3] and Gene Ontology (GO) enrichments of biological processes (Figure S1A).

The distribution of detected cell types corresponded to expected ratios in the cardiac tissue,[1–9] given the collection of all three layers of the heart wall—epicardium, myocardium, and endocardium (Figures 1A, and S1B)—with cardiomyocytes (CMs, 23%), endocardial endothelial cells (EECs, 30%), vascular endothelial cells (VECs, 10%), fibroblasts (FBs, 17%), and mesothelial cells (MESOs, 10%) as the main cell types (Figures 1B, and S1C).

We detected several putative marker genes for identifying the different cell types in spatial transcriptomics (Figure 1C, 1F, and 1G, and http://compbio2.mit.edu/scheart/; see Figure S1D for instructions). For example, EECs expressed several genes that allowed their distinction from VECs, including *LEPR*, *NPR3*, and *PCDH15*, whereas VECs expressed *GRB10*, *PREX2*, and *VWF* (Figures 1D–1F). We tested several of these marker genes with spatial transcriptomics, using a low-resolution technique (Visium, 10X Genomics) that captures 1–10 cells per spot (Figure 1F), and a high-resolution technique (Molecular Cartography, Resolve Biosciences) to reach sub-cellular resolution (Figures 1C and 1G), establishing specific marker genes for different cell types, such as *CHRM2* for CMs, *PCDH7* for EECs, *ABCA6* for FBs, *PRG4* for epicardial MESOs, and *MPZ* for Schwann cells (Figure 1G). However, some of the best cellular marker genes in snRNA-seq, such as *NPR3* for EECs (Figures 1D–1F), failed in the high-resolution spatial mapping due to their low abundance and/or specificity issues (Figures 1E, and S1E), indicating a necessity for validating cellular marker genes with spatial techniques, as they might not be identical to those uncovered in snRNA-seq.

High-definition map of the right atrial vasculature

To identify vascular cell types and cell subtypes, we used a combination of known and novel marker genes distinguishing a total of 11 populations, many of which were previously incompletely recovered (Figures 2A–2C, S1F and S1G).[1–3,26] We utilized the identified marker genes in spatial transcriptomics to characterize the cardiac vasculature—coronary arteries, arterioles, arterial and venous capillaries, and veins—in the samples (Figures 2D–2G) and confirmed the localized expression of mechano-activated transcription factor *KLF2* in the VECs (Figure 2H). KLF2 is known to integrate hemodynamic and pro-inflammatory stimuli to maintain vascular homeostasis and integrity[27,28] In snRNA-seq, most of the vascular endothelial subtypes showed downregulation of *KLF2* in the IHD group compared to control samples (Figure 2I), suggesting the emergence of endothelial cell dysfunction in the cardiac vasculature, which has been shown to be critical in the initiation and progression of cardiovascular disease.[29]

Transcriptional patterns reveal pro-inflammatory microvascular changes in the right atrium

To dissect similarities and differences in cardiac cell function on pathway level across main cell types in the three groups (IHD, IHF, and NIHF), we determined the differentially expressed genes across all cell types and cell subtypes using Nebula[30] (as described in STAR Methods), and interpreted the results using ingenuity pathway analysis (IPA, QIAGEN)[31] (Figure 3A, data and details available at http://compbio2.mit.edu/scheart/). The most significant enrichments in IHD and IHF included fibrosis for FBs and barrier disruption and hypertrophy for endocardial cells. In addition, both FBs and EECs were enriched for growth factors and mesenchymal transition, suggesting differentiation/activation of dysfunctional phenotypes, such as myoFBs,[32] and transition to pathological state, such as endocardial dysfunction,[33] which in an early stage disrupts normal tissue function and later on contributes to progression of the heart failure.

The top pathways in CMs and epicardial MESOs were the same across all three comparisons, i.e., EIF2 signaling, mitochondrial dysfunction, oxidative phosphorylation, and sirtuin signaling. Comparison of the pathway enrichments across the vascular cells and endothelial cell subtypes revealed the same four pathways that were detected in other main cell types as the most significant enrichments across all three conditions (Figures 3B, and S2A), suggesting accumulation of lipids, lipid peroxidation, and inflammation in the tissue due to impaired mitochondrial function, metabolic changes, cardiac hypertrophy, and increased production of reactive oxygen species (ROS) and inflammatory mediators. To validate these predictions in the IHF and control samples, we first utilized spatial transcriptomics (Figures 3C, and S2B, as described in STAR Methods). Compared to control, the IHF tissue exhibited increased expression of many pro-inflammatory mediators,

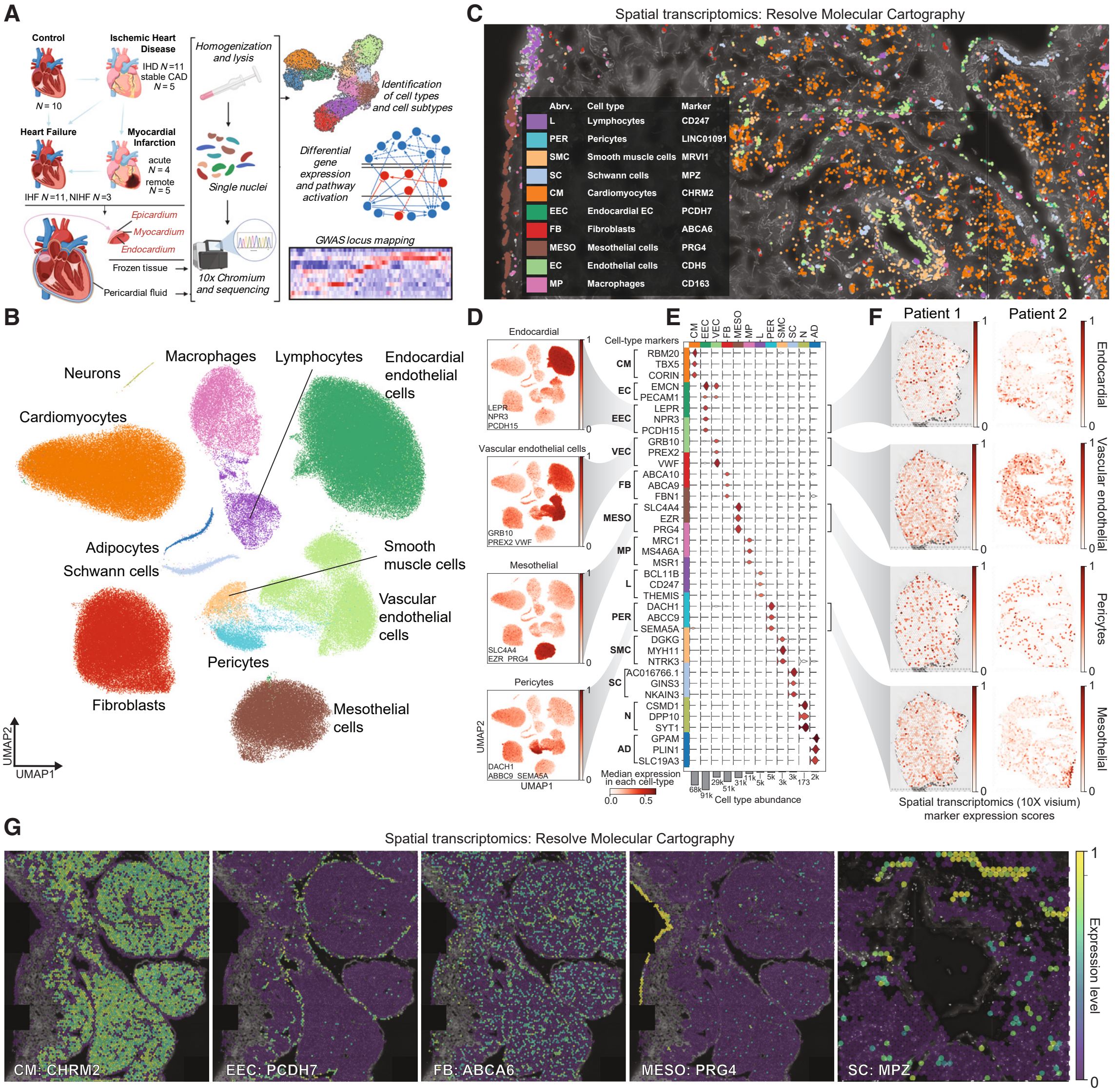

Figure 1. Cells of the *ex vivo* right atrium

(A) Overview of the samples and experimental approach. Created using BioRender.com.

(B) snRNA-seq data distinguishes 12 main cell types.

(C) Spatial transcriptomics image (Resolve Biosciences) of the cardiac tissue in IHD sample.

(D) snRNA-seq data highlight the expression of the marker genes for EEC (*LEPR*, *NPR3*, and *PCDH15*), VEC (*GRB10*, *PREX2*, and *VWF*), MESO (*SLC4A4*, *EZR*, and *PRG4*), and PER (*DACH1*, *ABCC9*, and *SEMA5A*).

(E) Violin plot of the marker genes from (D) and other main cell types from (B).

(F) Spatial expression (Visium, 10X Genomics) of the marker genes for EEC (*LEPR*, *NPR3*, and *PCDH15*), VEC (*GRB10*, *PREX2*, and *VWF*), MESO (*SLC4A4*, *EZR*, and *PRG4*), and PER (*DACH1*, *ABCC9*, and *SEMA5A*) in control (patient 1) and IHF (patient 2) samples.

(G) Spatial expression (Resolve Biosciences) of the CM (CHRM2), EEC (PCDH7), FB (ABCA6), MESO (PRG4), and Schwann cell (SC) (MPZ) marker genes in the right atrial tissue of IHD sample. MESO, epicardial mesothelial cell; L, lymphocyte; PER, pericyte; SMC, smooth muscle cell; N, neuron; AD, adipocyte.

such as *TRBC2* (an immune response activator)[34] and *CCL5* (a chemoattractant for blood monocytes, memory T helper cells and eosinophils).[35] In addition, the expression of key metabolic mediators and regulators, such as CD36, which mediates the uptake of long-chain fatty acids in cardiac tissue, and its regulator, nuclear receptor PPARα, was reduced in IHF compared to control. Impaired synthesis of CD36 has been shown to shorten myocardial energy supply, resulting in insufficient fatty acid uptake and accumulation of toxic lipids, ultimately leading to heart failure.[36–38] Consistent with the predictions, the expression of the key regulator of fatty acid and energy metabolism, PPARγ,[39] was also decreased in the IHF compared to control sections (Figures 3C, and S2B). Cytokine screening from the patient serum samples confirmed the presence of

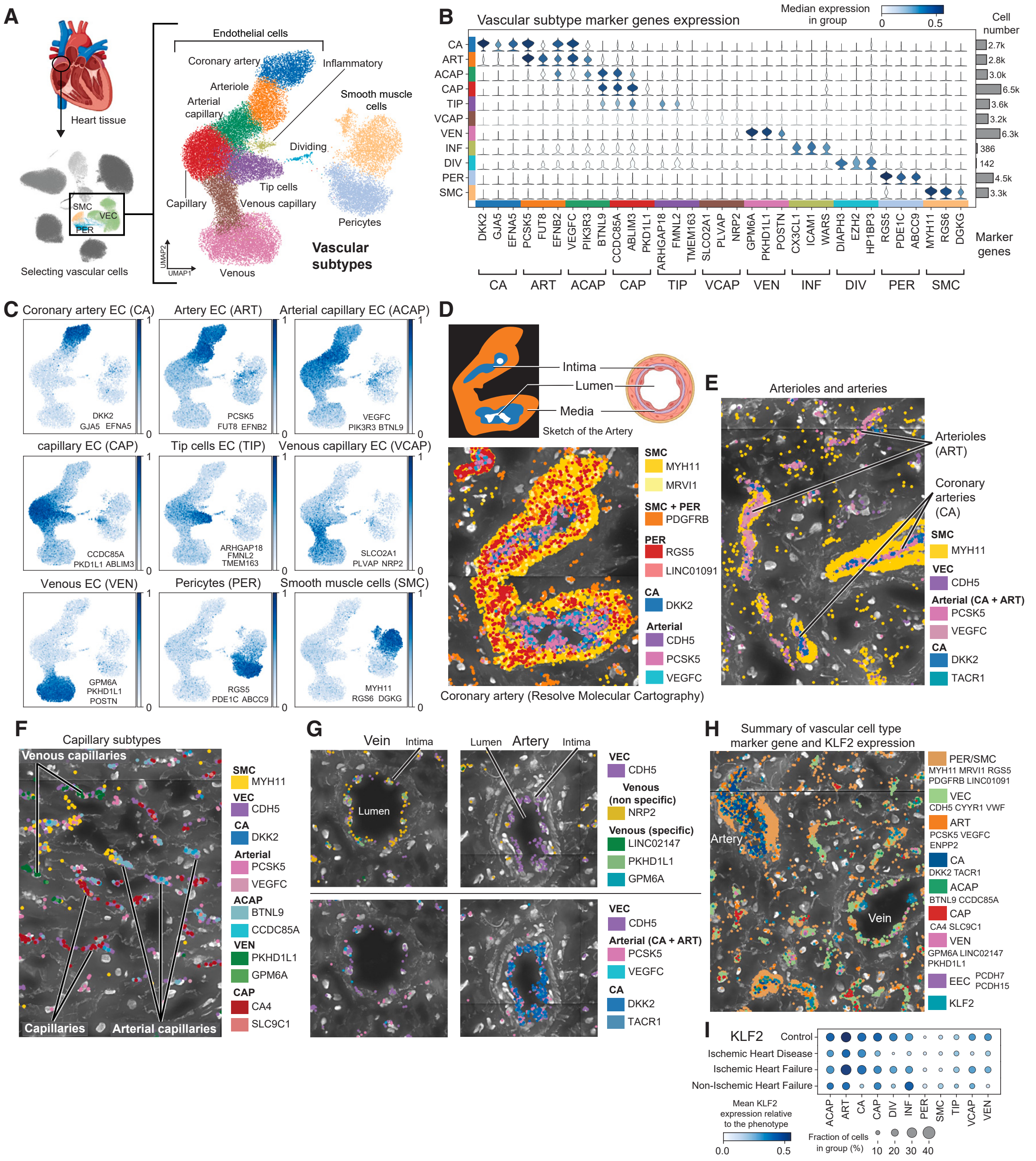

Figure 2. High-definition map of the right atrial vasculature

(A) Uniform manifold approximation and projection (UMAP) embedding of the snRNA-seq data for vascular cell subtypes, separating distinct EC, SMC, and PER populations. Heart image from BioRender.com.

(B) Marker genes for vascular subtypes: coronary artery (CA), arteriole (ART), arterial capillary (ACAP), capillary (CAP), tip cells (TIP), venous capillary (VCAP), venous (VEN), inflammatory ECs (INF), dividing ECs (DIV).

(C) UMAP embeddings showing vascular marker gene expression for CA (*DKK2*, *GJA5*, and *EFNA5*), ART (*PCSK5*, *FUT8*, and *EFNB2*), ACAP (*VEGFC*, *PIK3R3*, and *BTNL9*), CAP (*CCDC85A*, *ABLIM3*, and *PKD1L1*), TIP (*ARHGAP18*, *FMNL2*, and *TMEM163*), VCAP (*SLCO2A1*, *PLVAP*, and *NRP2*), VEN (*GPM6A*, *PKHD1L1*, and *POSTN*), PER (*RGS5*, *PDE1C*, and *ABCC9*), and SMC (*MYH11*, *RGS6*, and *DGKG*).

(D–G) Spatial images (Resolve Biosciences) of coronary arteries and arterioles (D and E), venous and arterial capillaries (F), and a vein and an artery (G) in right atrial tissue of control (D), IHF (E and F), and IHD (G) samples. Cross-section of an artery from BioRender.

(H) Summary image (Resolve Biosciences) of *KLF2* expression in an IHF sample.

(I) *KLF2* expression in snRNA-seq data across disease groups in vascular cell subtypes.

CellPress
OPEN ACCESS

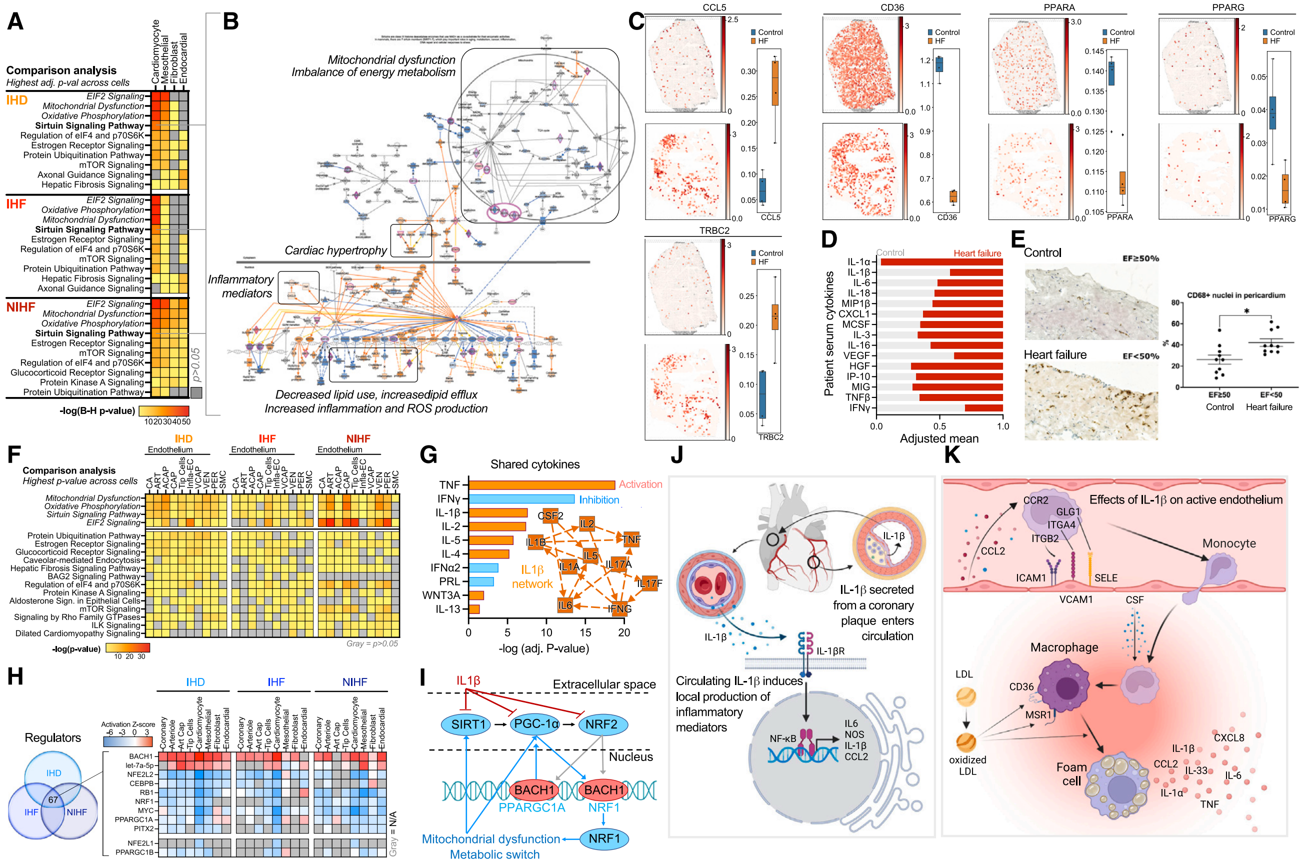

Figure 3. Heart disease promotes inflammatory microvascular dysfunction in the right atrium

(A) Top 10 canonical pathways with the highest significance score (B–H adjusted p value <0.05 for all) by IPA's comparison analysis across the main cell types, as described in STAR Methods, in IHD (N = 11), IHF (N = 11), and NIHF (N = 3) against control (N = 6). Full table and details available at http://compbio2.mit.edu/scheart/.

(B) Sirtuin signaling pathway from IPA for CMs in NIHF against control as a representative for all three groups. Blue indicates inhibition of the molecule, orange/red activation. Purple highlight marks significantly differentially expressed molecules in the dataset. Full version of the pathway figure can be found in Figure S2A.

(C) Spatial expression (Visium) of *TRBC*, *CCL5*, *CD36*, *PPARA*, and *PPARG* in a control and heart failure (HF) sample. For each gene, quantitation of the signal is shown across control (n = 4) and HF (n = 4) sections (right) and in one representative section (left), as described in STAR Methods. Whiskers show the maximum and minimum values, except for outliers (more than 1.5 times the interquartile).

(D) Scaled (0–1) mean of serum cytokines in patients with HF (N = 6) and controls (N = 7). No statistical significance.

(E) Quantitation of CD68$^+$ tissue MPs in pericardium of patients with HF (N = 10) and control group (N = 10) (*p < 0.0104). A representative immunohistostaining image provided for both groups, showing MPs in brown.

(F) Top 10 canonical pathways with the highest significance score (Fisher's exact test, p < 0.05 for all) across vascular cells within each group: IHD, IHF, and NIHF. Top 10 pathways for each condition were selected and p value is presented across all conditions.

(G) Top shared cytokines in IPA's Upstream Regulator analysis between IHD, IHF, NIHF, and disturbed flow in HUVECs dataset (N = 3 for d-flow and for control). IL-1β network from 2-h time point by IPA's machine-learning-based graphical summary is a representative for all four time points (2, 8, 14, and 32 h of IL-1β treatment compared to control [N = 3] in HAECs).

(H) Top overlapping upstream regulators (Fisher's exact test p < 0.05 for all) with similar activity patterns from IPA (microRNAs and transcriptional regulators only) for IHD, IHF, and NIHF in main cell types and four EC subtypes. Red is for predicted activity and blue for inhibition.

(I) Depiction of the connections between the potential mediators of the mitochondrial and metabolic changes.

(J) Overview of IL-1β signaling.

(K) Effects of IL-1β on activated endothelium.

(J) and (K) were created with BioRender.com.

many pro-inflammatory cytokines (Figure 3D), including interleukin (IL)-1-associated cytokines (IL-1α, IL-1β, IL-6, IL-18, MIP1b, and CXCL1), immune cell activators (IL-3 and IL-16), growth factors (MCSF, HGF, and vascular endothelial growth factor [VEGF]), and biomarkers for heart failure and left ventricular dysfunction, such as IP-10 (CXCL10), and MIG (CXCL9).[40]

IL-1 has been shown to contribute to all stages of atherosclerotic plaque life from initiation to rupture, although circulating IL-1 levels in patients rarely correlate with disease severity.[41] For example, circulating levels of IL-1α and IL-1β can be below detection level even in high-risk patients, yet their neutralization reduces adverse effects and improves disease outcomes.[42,43]

Here, IL-1α exhibited increased and IL-1β decreased presence in the HF group compared to control, although sample numbers were too small for statistical significance (Figure 3D). Taken together, the results suggested cytokine-induced pro-inflammatory activation and resulting accumulation of immune cells close to vasculature, endocardium, and epicardium in HF samples, which was then confirmed with immunostaining of $CD68^+$ tissue macrophages from HF and control samples (Figure 3E).

As suggested by the serum cytokine levels (Figure 3D), production of the angiogenic factor, VEGF,[44] was found to be suppressed in cardiac tissue cells in all three conditions (IHD, IHF, and NIHF), although induction of its regulator, hypoxia-inducible factor (HIF1α) was observed expectedly in ischemic (IHD and IHF) CMs (Figure S2C). A similar trend was observed across endothelial cell subtypes, suggesting inhibition of VEGF-regulated angiogenesis (Figure S3). Hypoxia-induced response patterns indicated endothelial activation across most subtypes and diseases, but endothelial movement and/or blood vessel maturation remained mostly inhibited, suggesting endothelial dysfunction and abnormal angiogenesis (Figure S4). Similarly, consistent with the serum cytokine levels and spatial transcriptomics results (Figures 3C and 3D), CCL2-, tumor necrosis factor (TNF)-, and CCL5-induced vasoconstriction was increased across endothelial subtypes and diseases (Figure S5), suggesting microvascular dysfunction with an inflammatory component.

A closer look into the differential expression enrichments in the vascular cells and cell subtypes revealed high similarity with the other main cell types, having the same four pathways, top the most significant enrichments across all three conditions (Figure 3F). To recreate endothelial dysfunction in an *in vitro* model, we exposed human umbilical vein endothelial cells (HUVECs) to disturbed flow (d-flow, i.e., oscillatory non-laminar shear stress).[45] We compared these changes to changes observed in the human cardiac tissue and saw decreased mitochondrial metabolic activity and increased HIF1α activity, cardiac hypertrophy signaling, and pro-inflammatory changes comparable to those in the tissue cells (Figure S6A). To screen for potential intercellular mediators of the observed changes, we extracted overlapping cytokines from the three conditions (IHD, IHF, NIHF) and d-flow endothelial cells using Upstream Regulator analysis of IPA (Figure 3G), and found several cytokines that were measured from the patient serum, such as TNF, which was more abundant in HF patient samples compared to controls, interferon (IFN) γ, which was more abundant in controls, and general enrichment of IL-1-related factors (Figure 3D). The analysis suggested IL-1β as a central regulator of the observed gene expression changes and a driver of the observed cytokine expression. To validate the finding, we performed RNA sequencing (RNA-seq) from IL-1β-stimulated human aortic endothelial cells (HAECs), confirming an IL-1β-centric interleukin network connecting the observed mediators in the cells (Figures 3G, and S6B). To further identify the potential regulators of the shared functional changes across cardiac cell types, we overlapped transcriptional regulators and microRNAs from Upstream Regulator analysis of IPA for the three conditions (IHD, IHF, and NIHF) (Figure 3H). Among the top regulators were transcription factors BACH1, NRF2 (*NFE2L2*), and NRF1 and a coactivator PGC1α (*PPARGC1A*) (Figure 3H), which are known regulators of mitochondrial function, metabolism, and cellular redox stress.[46–49] Their interactive network (Figure 3I) may thus partially explain the observed pathway changes in the main cell types. The notion was supported by the data collected from IL-1β-stimulated HAECs (Figure S6C), which illustrated a switch to anaerobic glycolysis, downregulation of mitochondrial biogenesis and metabolism, increased inflammation, ROS production, and hypertension signaling, highlighting suppression of SIRT1, PGC1α, and NRF2 as mediators of these events (Figure 3I). Collectively, these data suggest that IL-1β, which has been shown to be a component of coronary plaques[50] and produced by activated endothelia, smooth muscle cells, FBs, and immune cells in the body,[41] is either produced locally or travels to the right atrium from a distant site and induces local production of inflammatory mediators (Figure 3J), leading to recruitment of immune cells (Figure 3K). Accumulation of immune cells (Figures 3C and 3E), lipids (Figure 3B), and oxidative stress (Figure 3B) are known predecessors of foam cell formation (Figure 3K).[51]

Disease-driven immune cell accumulation causes chronic inflammation in the atrium

To explore the inflammatory changes in the right atrium, we next characterized the immune cell populations (Figure 4A) using previously established marker genes (Figure 4B)[1–3,52–56] and GO term enrichments (http://compbio2.mit.edu/scheart/). The immune clusters consisted of rich populations of macrophages (MPs) and T cells, in addition to dendritic cells, B cells, and plasma cells. Spatial transcriptomics of the atrial tissue illustrated immune cell clusters that localized to epicardial mesothelium and vascular endothelium (Figure 4C). The most prevalent immune cell population across all disease groups was MPs (Figure 4D). Other subtypes displayed more variability, suggesting increase in abundance from control to more severe disease (Figure 4D). In spatial analyses (described in STAR Methods), the MP marker gene, *CD163*, co-localized with infla-MP marker gene, *LYVE1*, with increased expression in IHF compared to control, consistent with previous findings (Figures 3C and 3E), whereas LT-CD4 marker gene, *IL7R*, expression overlapped only partially with the MP marker genes, although similarly higher expression in IHF sections compared to control was observed (Figures 4E, and S7A).

Chronic inflammatory response is part of adverse cardiac remodeling that precedes development of heart failure.[57] This remodeling is characterized by re-expression of the fetal gene program that includes natriuretic peptide B (*NPPB*) and α1 skeletal muscle actin (*ACTA1*),[58] both of which were observed in the IHF sections (Figures 4E, and S7A). To a lesser degree, the genes were also expressed in the control sections and co-localized to the same areas of the section, indicating localized disease-associated changes in gene expression before clinical manifestation of the disease. However, strong immunoglobulin expression was only observed in the IHF, consistent with chronic inflammatory state and accumulation of antibody deposits in the failing atrial myocardium.

As a confirmation for the results in the microvasculature that suggested lipid accumulation and early stages of foam cell formation (Figure 3), we detected two populations of lipid-associated MPs (LAMs), out of which LAM1 indicated partiality to

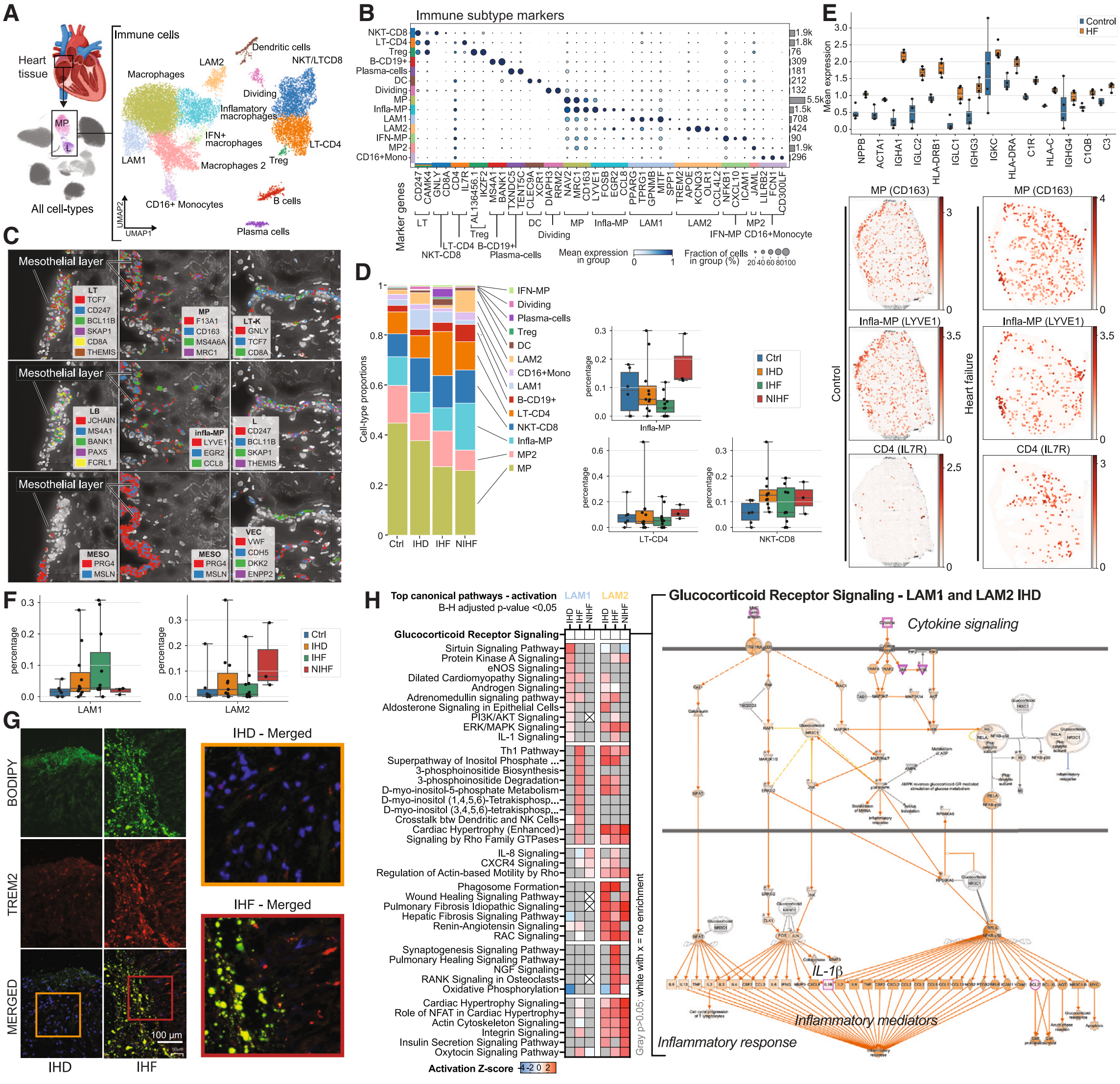

Figure 4. Disease-driven immune cell accumulation causes chronic inflammation in the atrium

(A) snRNA-seq for immune cell subtypes in right atrial tissue separating 14 populations. Heart image from BioRender.com.

(B) Marker genes for immune subtypes (DCs, MPs, LAMs, IFN-MPs, monocytes [mono]).

(C) Spatial expression images (Resolve Biosciences) of the right atrial tissue in IHD samples, depicting immune cells as marked by the expression of their marker genes near epicardial mesothelium and vasculature.

(D) Immune cell proportions in control, IHD, IHF, and NIHF samples. Bar charts depict the immune cell subtype proportions (%) of all cells in each sample for LT-CD4, infla-MP, and NKT-CD8 in control, IHD, IHF, and NIHF groups. Whiskers show the maximum and minimum values, except for outliers (more than 1.5 times the interquartile).

(E) Spatial expression (Visium) of *NPPB*, *ACTA1*, and immunoglobulins in a control and heart failure (HF) sample. Quantitation of the signal across control ($n = 4$) and HF ($n = 4$) sections is shown (as described in STAR Methods). Whiskers show the maximum and minimum values, except for outliers (more than 1.5 times the interquartile). Representative Visium images shown for *CD163*, *LYVE1*, and *IL7R* expression.

(F) LAM1 and LAM2 proportions (%) of all cells in each control, IHD, IHF, and NIHF sample depicted by groups. Whiskers show the maximum and minimum values, except for outliers (more than 1.5 times the interquartile).

(G) Immunofluorescence images of LAMs labeled using anti-TREM2 (red) and bodipy (green) in the epicardial side of the human right atrial appendage. Patients with IHF (middle panel) had increased number of LAMs compared to patients with IHD (left panel). Right panel shows zoomed region of interest from the merged image. Representative samples are shown.

(H) Top canonical pathways with highest activation Z scores for LAMs in IHD ($N = 11$), IHF ($N = 11$), and NIHF ($N = 3$) against control ($N = 6$) based on IPA analysis. Predicted pathway activation is shown in red and inhibition in blue based on the expression changes of the pathway molecules in the dataset and current literature-based knowledge curated into the QIAGEN Knowledge Base. Gray is for enriched pathways with (B)–(H) adjusted p value over 0.05 and white with x for no enrichment. All pathways with activation Z score are statistically significant (B–H adjusted p value <0.05). A representative glucocorticoid receptor pathway chart is shown.

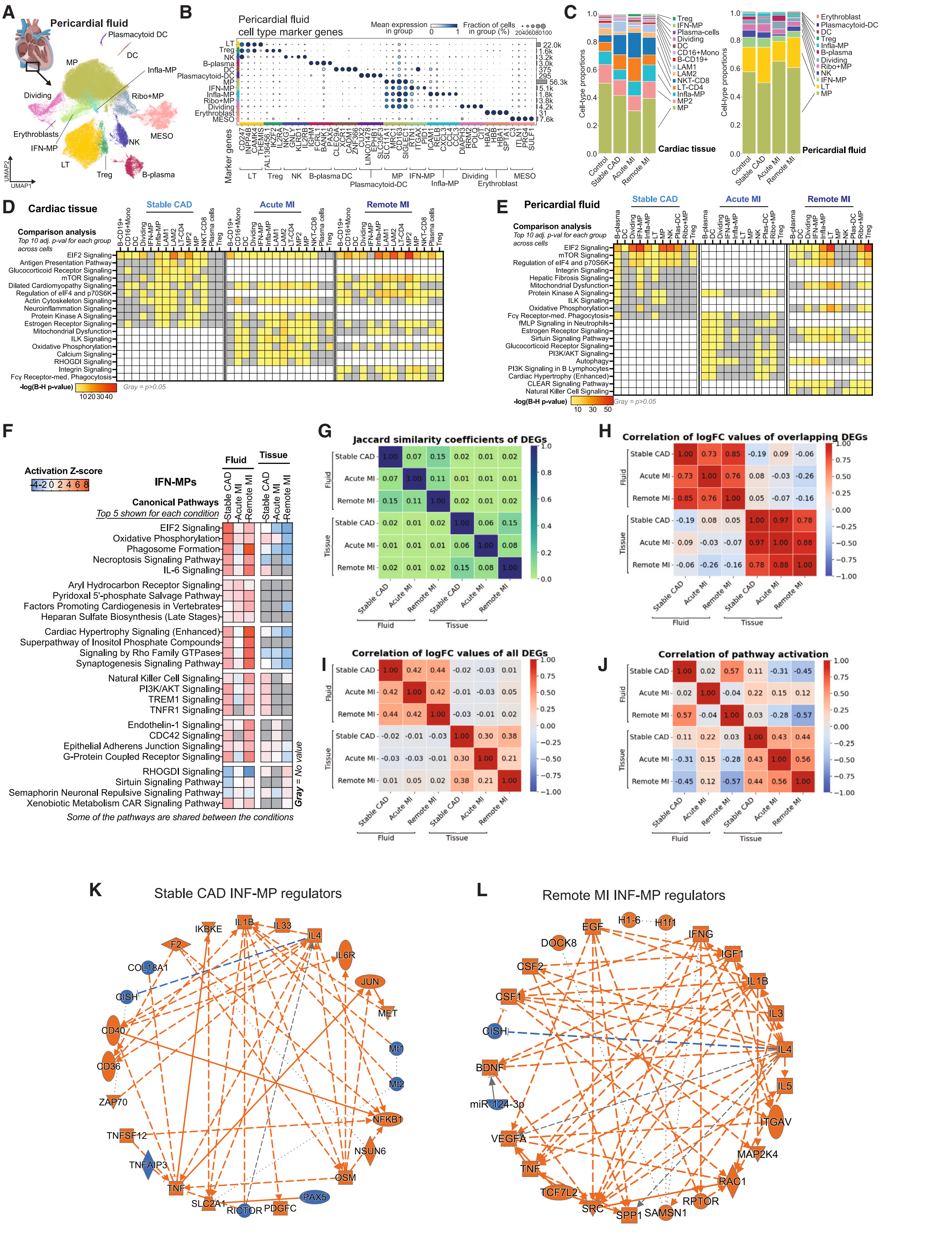

(legend on next page)

ischemic disease and LAM2 to NIHF (Figure 4F). We confirmed the colocalization of an established LAM marker TREM2[59] with lipid droplets in IHF sections and observed increased signal in IHF compared to IHD (Figure 4G).

Exploration of pathway enrichments for differentially expressed genes in the LAM populations revealed differences in the pathway numbers, their sharing, and in the enriched pathways themselves. Altogether, the LAM1 population enriched for 118 and LAM2 for 228 pathways (B–H adjusted p value <0.05). Ten percent of the LAM1 pathways were not found from the LAM2 populations (Data S1). In IHD, the top activated pathways (based on activation Z score by IPA) were sirtuin signaling pathway, protein kinase A signaling, eNOS signaling, and dilated cardiomyopathy signaling for the LAM1 population and cardiac hypertrophy (enhanced), phagosome formation, wound healing and fibrosis signaling pathways for LAM2. Half of the enriched LAM1 pathways were shared with LAM2, including pathways for oxidative phosphorylation, mitochondrial dysfunction, sirtuin signaling, estrogen receptor signaling, and glucocorticoid receptor signaling, comparably to the main cell types (Figures 3A and 3F, and Data S1). Glucocorticoid receptor signaling was among the most significant enrichments in all but one LAM group (LAM2, IHF) (Figure 4H and http://compbio2.mit.edu/scheart/), highlighting increased cytokine-mediated production of IL-1β, among other key pro-inflammatory mediators. The LAM1-specific pathways included IL-1 and IL-6 signaling, whereas LAM2-specific populations consisted of neuronal-, inflammatory-, cardiac-hypertrophy-, and growth-factor-related pathways (Data S1). Only 34% of the LAM1 pathways in IHD were shared with the LAM1 pathways in IHF and none with NIHF (Figure 4H, and Data S1). LAM1s in NIHF only enriched for 11 pathways altogether, seven of which were shared with IHF. LAM2 populations shared more pathways between conditions (Figure 4H, and Data S1). For example, only 13% of the IHD-enriched pathways were unique to IHD, 27% were shared with IHF, 21% with NIHF, and 39% were shared by all three conditions. Overall, LAM2 in IHF enriched for more pathways than the other two conditions and therefore also had more unique pathways. Collectively, the pathway enrichments in the LAM1 population consisted mostly of extracellular signaling-, metabolism-, and senescence-related terms, and in the LAM2 population, of pathway terms that intersect with cardiovascular disease, inflammation, and cancer, suggesting response to environmental cues for LAM1s and pro-disease signaling for LAM2s.

Pericardial fluid cells reflect the changes in disease states

Pericardial fluid is an enriched milieu of cytokines, growth factors, and cardiac hormones that reflect and regulate overall heart function, yet its cell composition has not been thoroughly examined.[60] Given the detected accumulation of immune cell clusters to the epicardial mesothelium in spatial transcriptomics (Figures 4C, 3C and 3E), we explored the cell populations in paired right atrial tissue and pericardial fluid samples of IHD patients to gain insight into the potential interactions between the tissue and the fluid. Although the tissue sample is limited to the right atrium, the pericardial fluid provides a more integrated view of the cardiac function, comprising signals from all parts of the heart.

We focused on three distinct groups: patients with stable coronary artery disease who underwent elective surgery (stable CAD), patients with acute MI, and patients who had suffered infarction earlier in life (remote MI) to compare stable disease to acute event and recovery after the acute event. Expectedly, snRNA-seq showed pericardial fluid to be enriched for immune cells (Figures 5A and 5B). The cell proportions between the tissue and the fluid showed both similarities and differences (Figure 5C). The largest population in both was MPs, followed by lymphocytes and natural killer cells. Cardiac-tissue-specific populations included LAMs, monocytes, and plasma cells, whereas pericardial-fluid-specific populations included Ribo+MPs (i.e., MPs with increased expression of ribosomal genes), plasmacytoid dendritic cells (plas-DCs), and erythroblasts. Infla-MPs were proportionally larger population in the tissue compared to the fluid, whereas IFN-MPs were more predominant among the fluid immune cells.

In the tissue samples, MP2 population seemed to be more prevalent in the control samples, whereas NKT-CD8 and infla-MPs were more abundant in stable CAD and MI groups (Figure S7B). The LT-CD4 population was more numerous in the stable CAD and acute MI, but in remote MI the levels resembled control levels. In the pericardial fluid, MP, LT, infla-MP, and regulatory T (Treg) proportions were more variable in the IHD groups compared to control, suggesting active exchange between the tissue and the fluid and response to acute changes in disease signaling (Figure S7B).

Differentially expressed gene enrichments were similar, although not identical, in the pericardial fluid and corresponding tissue with 66%–68% of all enriched pathways being shared between the fluid and tissue cells in each of the three groups

Figure 5. Pericardial fluid cells reflect the changes in disease states

(A and B) (A) snRNA-seq data and (B) marker genes for immune cell subtypes in pericardial fluid. Heart image by BioRender.com.

(C) Immune cell proportions in cardiac tissue and corresponding pericardial fluid samples.

(D and E) Top 10 pathways with highest significance score in IPA comparison analysis (B–H adjusted p value <0.05) in cardiac tissue (D) and corresponding pericardial fluid samples (E) in stable CAD ($N = 5$), acute MI ($N = 4$), and remote MI ($N = 5$) compared to control ($N = 4$). Only the top 10 for each group are shown. Full data can be explored at http://compbio2.mit.edu/scheart/.

(F) Top pathways based on activation Z score from IPA for each condition in fluid and tissue IFN-MPs. Positive values indicate pathway activation based on underlying gene expression patterns and blue pathway inhibition.

(G) Jaccard similarity coefficiency for differentially expressed genes (DEGs) in IFN-MPs.

(H) Correlation of log fold change (logFC) values of overlapping DEGs (i.e., found in all compared groups) in IFN-MPs.

(I) Correlation of logFC values of all DEGs in IFN-MPs.

(J) Correlation of activation Z scores for enriched pathways in IFN-MPs.

(K and L) Regulator networks for stable CAD and remote MI in IFN-MPs from IPA's graphical summary.

(Figures 5D and 5E and http://compbio2.mit.edu/scheart/). Stable CAD and remote MI had 100% overlap in pathways enriched in each, both in fluid and tissue samples, although the rank order in remote MI was a mix of stable CAD and acute MI, likely due to its shared disease component with both groups (Figures 5D, 5E, and Data S1, and http://compbio2.mit.edu/scheart/). Also, 12% and 18% of enriched pathways were unique to acute MI in fluid (63 unique pathways) and tissue (83 unique pathways), respectively, including terms related to IL-1 signaling and metabolism in the fluid and secondary messenger signaling, interleukins, and metabolism in the tissue. In tissue, MPs were the most active populations in all three conditions, promoting immune response, intracellular and second messenger signaling, and cellular stress and injury in stable CAD and cardiovascular signaling and disease-specific pathways in acute MI, leading to glucocorticoid-mediated CCL2 and CCL3 production in both (Figures S7C and S7D). CCL2 is a known regulator of MP recruitment and polarization in inflammation[61] and CCL3 is an acute pro-inflammatory recruiter and activator of leukocytes in the heart tissue, as well as an inducer of TNF and IFN production that is associated with CM injury, cardiac dysfunction, and delayed ventricular repolarization.[62]

For a closer look into the molecular intersection of the pericardial fluid and tissue cells, we chose one of the most prominent specialized populations in the ischemic patients, IFN-MPs (Figure 5C). General overview of the top enriched canonical pathways for each condition based on activation *Z* scores showed no immediate overlap of activation patterns between fluid and tissue cells (Figure 5F). When estimating the similarity of the differentially expressed genes across different conditions (stable CAD, acute MI, and remote MI) and different sample types (cardiac tissue and pericardial fluid), the overlap was found to be weak (Figure 5G). Although the correlation of logFC values for the overlapping differentially expressed genes was good within sample types across conditions (Figure 5H), the overall correlation of logFC values for all differentially expressed genes was significantly weaker and resulted in poor correlation of activation *Z* scores on the pathway level (Figures 5I and 5J), suggesting condition- and environment-specific gene expression patterns and pathway activation across sample types and conditions.

The fluid IFN-MPs in stable CAD and remote MI revealed higher similarity in the enriched pathways (B-H adjusted p value <0.05) and activation *Z* scores than the other group comparisons (Figure 5J and http://compbio2.mit.edu/scheart/). For example, phagosome formation, IL-6 signaling, and cardiac hypertrophy (enhanced) pathways were among the top pathways in both stable CAD and remote MI. However, there were underlying differences in the pathway molecules. For instance, in the hypertrophy pathway of the remote MI, the analysis highlighted TNF, insulin-like growth factor 1, and pro-inflammatory cytokines as main upstream molecules activating the signaling cascades, whereas, in stable CAD, endothelin-1, TNF, and integrins were highlighted (data not shown). Acute MI enriched for similar pathways compared to stable CAD and remote MI, but the activation *Z* score was opposite in many cases. Dissection of the biological and disease-associated functions of the IFN-MPs suggested increased blood cell activation, recruitment, movement, adhesion, migration, engulfment, and atherosclerosis in stable CAD, decreased lipid droplet accumulation, blood platelet aggregation, blood cell accumulation, and cell movement in acute MI, and increased infection, cell movement, cell migration, proliferation, engulfment, angiogenesis, fibrogenesis, and vascular development in remote MI (Data S1). Central factors orchestrating these changes included IL-1β (stable CAD and remote MI) and IFNγ (remote MI) (Figures 5K and 5L). Taken together, the results suggest that presence of IFN-MPs in the tissue correlates with advanced disease and chronic inflammatory state of the right atrium. Moreover, the data suggest that the IFN-MP population present in the pericardial fluid reflects the changes in the tissue function and responds to environmental cues of acute and chronic disease states. However, the fluid results are not an exact replica of the right atrial tissue results, due to fluid cells being a collection of cells that can originate from any part of the heart that now reside in a distinct microenvironment specific to the pericardial compartment.

Vascular cells interact with IFN-responsive immune cells

To dissect the co-expression patterns in the IFN-MP population in the pericardial fluid, we used a gene expression module approach that highlighted several functional gene clusters (Figure 6A and http://compbio2.mit.edu/scheart/). The proportion of the cells was generally lower in the tissue of stable CAD and MI patients and higher in their pericardial fluid, suggesting mobilization of the cells in the earlier and acute stages of the disease, whereas, in the more advanced disease, the data suggested potential accumulation in the tissue (Figure 6B). In spatial images (Visium), *CXCL10*, a marker gene for the IFN-MP population, was detected in IHF, clustering in the myocardium but not in the control sections (Figures 6C, and S8A). CXCL10 (IP-10, detected in the patient serum samples; Figure 3D) is a known chemoattractant and polarizing factor for various immune cells, promoter of T cell adhesion to endothelial cells (ECs), inhibitor of angiogenesis, and a biomarker for heart failure and left ventricular dysfunction.[40,63,64] In the higher-resolution spatial images, *CXCL10* clusters were found in the vicinity of vasculature and endocardium, colocalizing with MPs, lymphocytes, and the marker genes for infla-MPs (*CLL8* and *EGR2P*) (Figures 6D, and S8B). In the vascular cells, module analysis confirmed the presence of an IFN module comprising several IHD-genome-wide association study (GWAS) genes (Figure 6E). The IFN module was present across all vascular cell subtypes and enriched for several related pathways (Figure 6F), confirming the connection between the vascular cells and the IFN-responsive immune cells at the transcriptional level.

Disease-associated genetic variation affects disease-relevant modules across cell types

Next, we sought to dissect IHD-associated GWAS gene interactomes across different cell types by annotating our modules of coordinated gene expression changes with GWAS-linked genes in each cell type (all data can be explored at http://compbio2.mit.edu/scheart/). One of the genes highlighted by the module analysis was *SVIL*, which was present both in the vascular and pericardial fluid cells (Figures 7A and 7B). It resides in a GWAS locus on chromosome 10 that gives rise to two IHD-associated genes, *SVIL* and

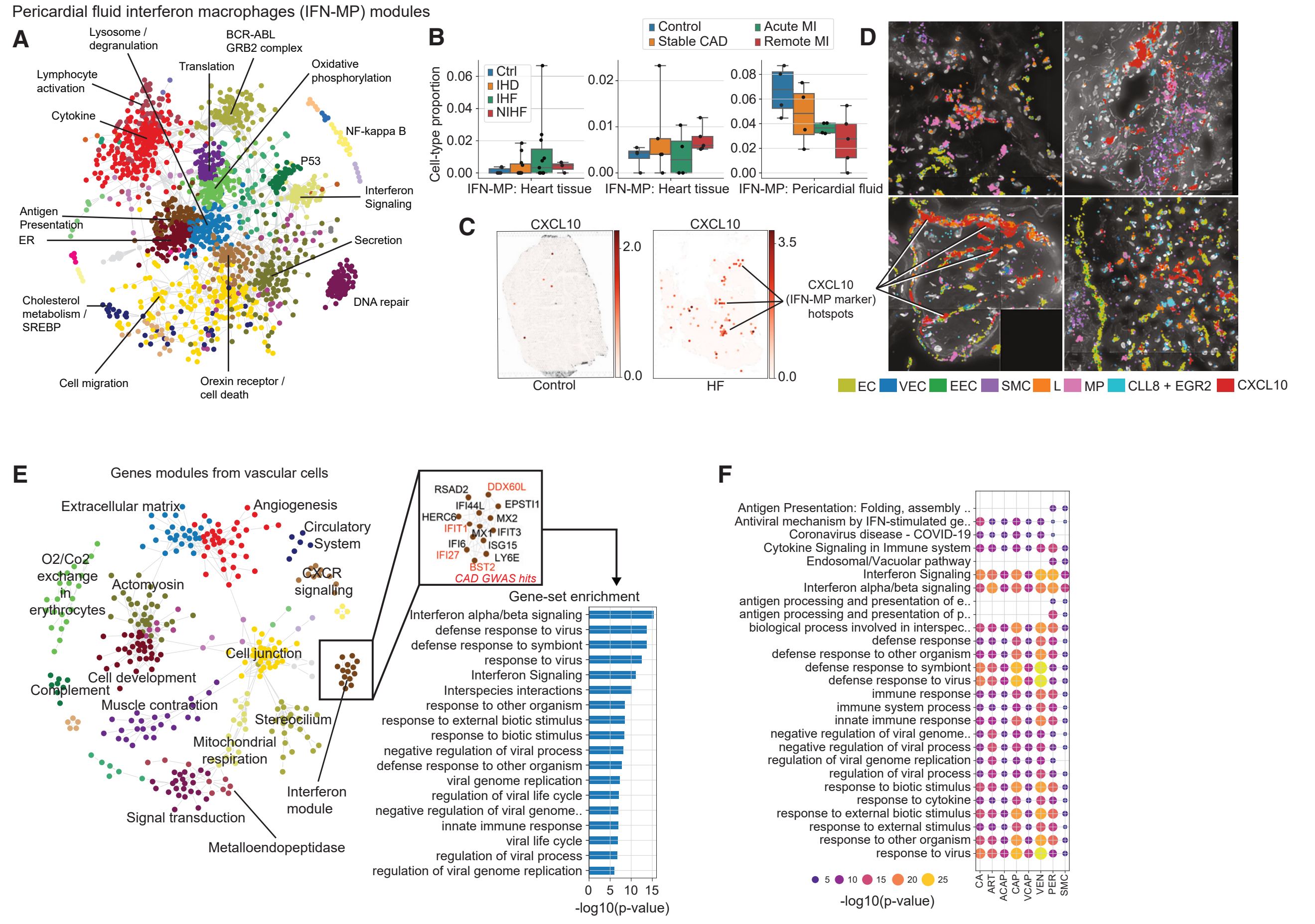

Figure 6. Disease-associated genetic variation affects disease-relevant modules across cell types

(A) Gene expression modules in IFN-MPs of the pericardial fluid.

(B) IFN-MP proportions (%) of all cells depicted by group. Whiskers show the maximum and minimum values, except for outliers (more than 1.5 times the interquartile).

(C) Representative Visium images shown for *CXCL10* expression.

(D) Spatial images (Resolve) for EC (*EMCN*, *ERG*, *PECAM1*, *CDH5*, *VWF*), VEC (*DKK2*, *ENPP2*, *PCSK5*, *CYYR1*), EEC (*PCDH7*), SMC (*NTRK3*, *MRVII*), *L (BCL11B*, *CD247*, *SKAP1*, *THEMIS*), MP (*CD163*, *MRC1*, *F13A1*, *MS4A6A*), inflammatory (*CCL8* and *EGR2*), and *CXCL10* gene expression in IHD (upper panel and lower left) and control (lower right) samples.

(E) Gene expression modules for vascular cells (all subtypes combined) highlighting interferon module, its genes (GWAS-linked genes in red), and pathway enrichments.

(F) Enrichments of the interferon module across vascular cells.

JCAD, that link to the same regulatory element bearing several IHD-associated variants (Figure 7C). *JCAD* has been previously implicated in endothelial dysfunction, pathological angiogenesis, and atherosclerotic plaque formation,[65] whereas the role of *SVIL* in cardiovascular disease remains unknown, but it may promote angiogenesis and epithelial to mesenchymal transition.[66]

SVIL was present in modules across multiple cell types and involved in several cell-type-specific functions. In smooth muscle cells, *SVIL* resided in a module that enriched for glycosaminoglycan-, proteoglycan-, and chondroitin sulfate-related pathways (Figures 7A, and S9A), whereas the *JCAD* module consisted of three genes, *JCAD*, *CFAP36*, and *PALMD*, which have been shown to regulate aortic valve calcification via altered glycolysis and inflammation.[67] Proteoglycans regulate extracellular matrix composition and organization, partake in inflammatory processes through leukocyte adhesion and migration, and are central modifiers of ischemic and pressure-overload-related cardiac remodeling that leads to heart failure.[68] In pericardial cells, the *SVIL* module was enriched for cell communication, intracellular signal transduction, and regulation of cell adhesion (Figures 7B, and S9B). *SVIL* was also involved in modules in T/NK cells, enriched for cell signaling and communication; in B-plasma cells for T cell differentiation, activation, and signaling; and in MESOs for extracellular matrix organization, blood vessel development, and morphogenesis of an anatomical structure (Figures S9C–S9F).

Although GWASs successfully associate genomic loci with diseases, they are restricted in their ability to dissect complex

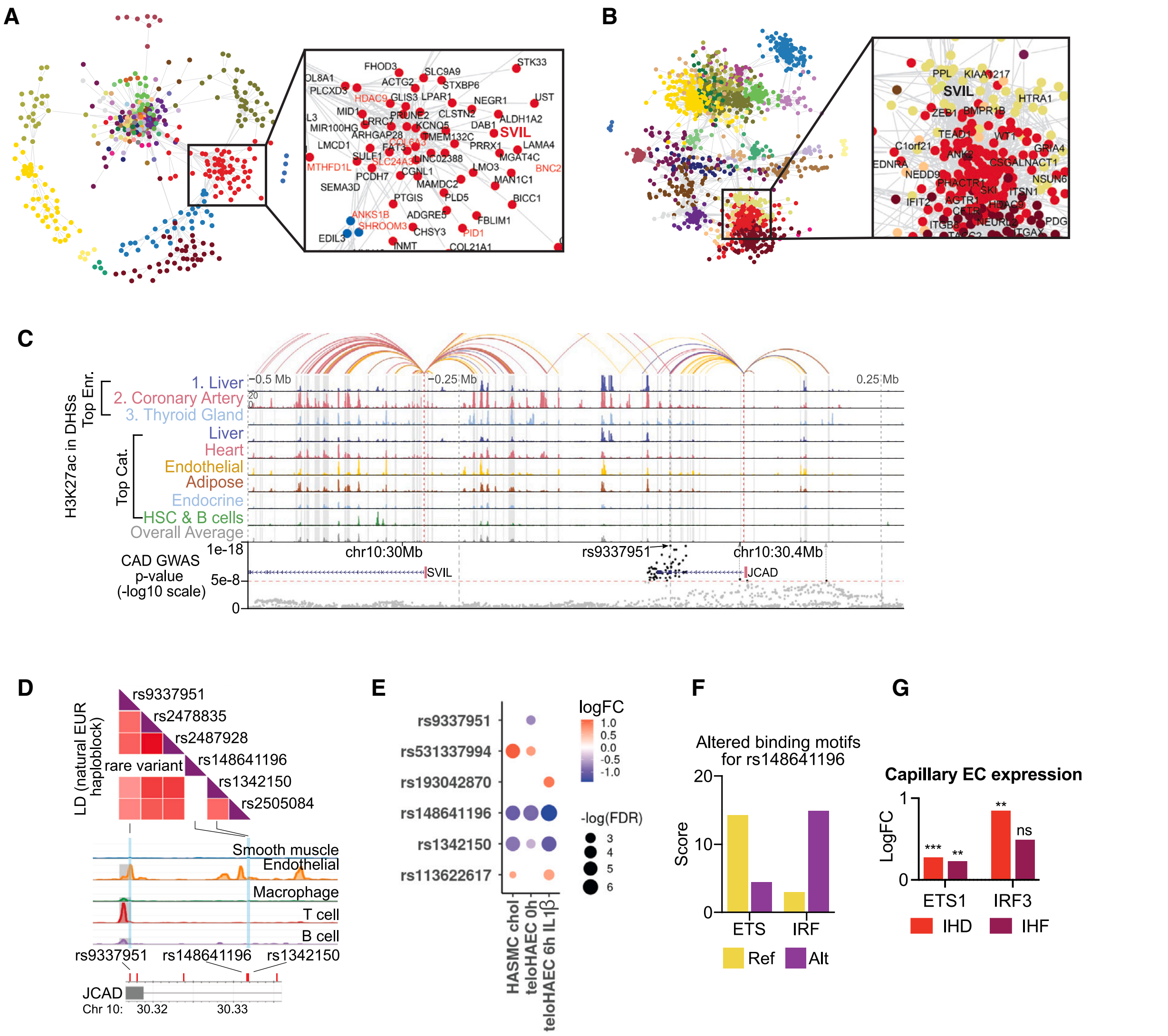

Figure 7. Dissection of *JCAD/SVIL* locus

(A and B) Gene expression module for SVIL in SMCs (A) and pericardial fluid cells (B). GWAS-linked genes in red.

(C) Epimap linking of the *JCAD/SVIL* locus.

(D) Illustration of LD SNPs of the natural European haploblock combined with the single-cell assay for transposase-accessible chromatin with sequencing (scATAC-seq) data from human coronary arteries generated using LDlink.

(E) Allele-specific enhancer activity measured with STARR-seq in teloHAECs under basal and inflammatory conditions (6 h IL-1β) and HASMCs subjected to cholesterol loading for 24 h. SNPs demonstrating significant changes (false discovery rate [FDR] <0.1) in enhancer activity.

(F) Transcription factor binding motifs altered by rs148641196. Position weight matrix scores shown for reference and alternate.

(G) Changes in transcription factor *ETS1* and *IRF3* gene expression in capillary ECs in IHD and IHF in snRNA-seq by Nebula. (**$p < 0.01$, ***$p < 0.001$)

causality, as they do not specify the causal variant(s). For example, the *JCAD/SVIL* locus investigated in this study associates significantly with IHD and several of the top associated variants reside in the regulatory elements that are active in the heart and coronary arteries (Figures 7C, and S10A). To bridge the gap between GWAS association and biological understanding of underlying disease risk, we utilized our recently published EpiMap analysis,[69] which suggested rs9337951 as the top candidate (p = 1e−17; Figures 7C and 7D). To further identify individual causal variants, we tested each regulatory variant for its effect on self-transcribing active regulatory region sequencing (STARR-seq) reporter gene expression in HAECs and in primary human aortic smooth muscle cells (HASMCs) subjected to pro-atherogenic stimuli (IL-1β and cholesterol loading). Altogether, six regions of 200 bp were studied representing haplotypes that encompassed nine common variants and seven rare variants in the European population. Of these, two common variants, namely rs9337951 and rs1342150, and four rare variants, namely rs531337994, rs148641196, rs113622617, and rs193042870, exhibited allele-specific enhancer activity (Figure 7E, and

Table S1). To validate the regulatory effect of the identified common variants, we used CRISPR technology to perturb the enhancer activity. First, we performed CRISPR-mediated deletion of the regulatory elements in teloHAECs, resulting in 50%–70% decrease in *JCAD* expression (Figures S10B and C) but no change in *SVIL* expression (data not shown). This effect was strongest for the rs9337951, the top candidate suggested by the EpiMap analysis, likely due to its exonic location (Figures S10B–S10F). To ensure no mRNA sequence itself was targeted in the CRISPR perturbation, we additionally performed a CRISPR inhibition experiment where the regulatory elements were targeted with the transcriptional repressor dCas9-KRAB-MeCP2. This approach resulted in similar effects for both targeted regions where, on average, 70% repression in *JCAD* expression was seen, thus confirming the role of variant-carrying enhancers in gene regulation (Figures S10D–S10F). Although *SVIL* expression was not changed in the static *in vitro* model in basal conditions, we cannot rule out the possibility that the dynamics of the enhancer function are stimuli specific. In VECs, *SVIL* expression was strongest in dividing (not shown), inflammatory, and tip cells (Figure S10G), which all represent more specific cell states, highlighting the importance of human tissue data and disease-relevant stimulus in GWAS dissection. In addition to vascular cells, *SVIL* expression was detected in all main cell types (Figure S10H), with strongest expression in CMs and FBs, but was not due to ambient RNA contamination (Figure S10I).

To explore potential mechanisms of action for rs148641196, a rare variant with the highest allele-specific activity in reporter assay (Figure 7E), we utilized motif analysis.[70,71] The analysis suggested that rs148641196 restores a functional interferon regulatory factor (IRF) binding site from an ETS binding site (Figure 7F), which could potentially promote inflammation-induced dysregulation of the locus during disease development. In our dataset, the expression of both *ETS1* and *IRF3* was upregulated in capillary ECs in IHD and IHF (Figure 7G). ETS1 is an essential factor for vascular angiogenesis,[72] whereas cardiac-damage-induced IRF3-IFN activation has been linked to expression of inflammatory cytokines and chemokines, inflammatory cell infiltration of the heart, and fatal response to MI.[73] Although the rare variant is unlikely to explain general disease-linked signal arising from the *JCAD/SVIL* locus, it suggests that, when present, rare variants such as rs148641196 that are inherited with common GWAS SNPs may significantly increase an individual's risk for a disease.

Taken together, these data illustrate how a disease-associated variant can affect expression of more than one gene in several cell types, partaking in distinct functions in each through functional gene expression modules. They also show how less-characterized genes can be linked to functions based on their co-expression patterns, which may help to identify novel disease-associated connections beyond previously established pathways.

DISCUSSION

One of the hallmarks of cardiovascular disease progression is the switch from fatty acids to glucose, and accompanied suppression of mitochondrial oxidative phosphorylation, together with a gradual decrease in mitochondrial biogenesis, and overall downregulation of oxidative metabolism.[74] In this study, we documented the metabolic switch in the earlier stage of the disease prior to major hypertrophic and inflammatory changes of the tissue, across various right atrial cell types from CMs to vascular and immune cells. Although metabolic reprogramming would be expected in the hypertrophic tissue of the ventricles, it is more surprising to find it in the right atrial appendage for its remote location, as it suggests major changes in the tissue function far away from the atherosclerotic plaque and right next to the site where heartbeat initiates. Furthermore, metabolic reprogramming is present in the atrial tissue both in the IHF and NIHF, suggesting underlying mechanistic similarities between the conditions.

As the main causal changes for the disorders studied here are expected to manifest elsewhere, further dissection of the data suggested the pro-inflammatory molecule, IL-1β, as the initiator of the metabolic reprogramming and subsequent inflammation in the remote location profiled in this study. IL-1β is highly produced and systemically released by activated MPs, FBs, smooth muscle cells, and ECs in response to danger signals that activate pro-inflammatory processes, whereas IL-1α is a pro-cytokine that is not processed or released outside the cell, unless the cell is injured, which makes it a marker of cell damage.[75] In this study, higher levels of IL-1α in serum samples of the patients with HF compared to the control samples may have been an indicator of vascular inflammation,[41] as the gene expression patterns of the CMs, MESOs, vascular cells, and various immune cells suggested coronary microvascular dysfunction with an inflammatory component as one of the causal mediators of disease-associated changes in the tissue. However, it should be noted that the main regulator may also be one of the factors downstream of IL-1β activity, such as IL-6, which has been suggested to play a causal role in IHD.[76]

Inflammation has long been known to play a detrimental role in the atherosclerotic plaques in IHD[77] and to correlate with increased risk for cardiovascular events,[78,79] which has prompted the question of whether inflammation should be targeted in the cardiovascular disease treatment more broadly. As the data presented here suggest an active role for the interleukins in initiation of severe cardiovascular remodeling far from the acute site, targeting low residual inflammation in cardiovascular disease using widely available drugs seems reasonable. Although measurement of IL-1β in the clinic is not feasible, C-reactive protein (CRP) is a correlate for IL-1 and IL-6 activity[80–82] that is available in every laboratory. Mechanistically, CRP release from hepatocytes is a reaction to circulating IL-6 levels, which, in turn, are controlled by IL-1β production and release. As drugs targeting IL-1β and IL-6 are available for use in cardiovascular disease treatment,[42,83–87] further investigation of these interleukins in the disease manifestation and progression in adult human tissue and human disease models is critical.

Limitations of the study

Due to the higher number of men compared to women among the study participants, sex-specific effects cannot be evaluated. All participants of the study are Finns, which increases the

statistical power of the small sample groups due to the homogeneity of the Finnish population but may limit the direct extrapolation of the results to other ethnicities. As the samples were collected during open-heart surgeries, no healthy control individuals could be included, and the control patients were selected among individuals who were operated due to mitral valve regurgitation or valvular heart disease and had no other significant cardiovascular disease.

Conclusions

The integrated information from cell and cell-subtype-specific gene expression changes across various cardiovascular traits can be used to better understand the complex molecular disease mechanisms behind the traits and to identify critical drivers of cardiovascular disease, as well as to guide the development of novel therapeutic strategies for cardiovascular disease treatment. The data collected in this study suggest that investigation of human cardiovascular disease should expand beyond the obvious sites of disease, such as atherosclerotic plaques and left ventricle, to gain insight into the pathological remodeling occurring in distant yet functionally important parts of the heart that may play a role in the sustenance and progression of the disease.

STAR★METHODS

Detailed methods are provided in the online version of this paper and include the following:

- KEY RESOURCES TABLE
- RESOURCE AVAILABILITY
  - ○ Lead contact
  - ○ Materials availability
  - ○ Data and code availability
- EXPERIMENTAL MODEL AND STUDY PARTICIPANT DETAILS
  - ○ Human biopsies - sample selection
  - ○ Endothelial cells
- METHOD DETAILS
  - ○ Histochemistry
  - ○ Isolation of nuclei from *ex vivo* cardiac tissue and pericardial fluid
  - ○ Droplet-based snRNA-seq
  - ○ Endothelial cells – Disturbed flow
  - ○ Endothelial cells – IL-1β stimulation
  - ○ Resolve molecular cartography
  - ○ Visium spatial transcriptomics
  - ○ Cytokine screening from serum samples
  - ○ Immunohistochemistry
  - ○ Allelic activity reporter assay
  - ○ CRISPR-RNP transfection
  - ○ CRISPRi: Plasmid transfection
- QUANTIFICATION AND STATISTICAL ANALYSIS
  - ○ Bioinformatics preprocessing of snRNA-seq samples
  - ○ Expression correction, sample heterogeneity correction, embedding and clustering
  - ○ Cell type identification
  - ○ Cell subtyping
  - ○ Differential expression analysis
  - ○ RNA-seq on HUVECs for disturbed flow experiment
  - ○ RNA-seq on HAECs for IL-1β time course analysis
  - ○ Ingenuity Pathway Analysis
  - ○ Resolve molecular cartography
  - ○ Visium spatial transcriptomics
  - ○ Gene expression modules
  - ○ GWAS linked genes
  - ○ Prediction of TF binding motif disruption

SUPPLEMENTAL INFORMATION

Supplemental information can be found online at https://doi.org/10.1016/j.xcrm.2024.101556.

ACKNOWLEDGMENTS

We thank Ms. Sari Mäki and Ms. Teija Kanasuo for their contribution to the immunostainings and multiplex cytokine measurements, respectively. This work was supported by The Academy of Finland grants 342074 (S.L.K.), 333021 (M.U.K.),325510 (P.T.); Clinical Research Fund (EVO) of Turku University Hospital, Turku, Finland (T.K.); European Research Council (ERC) grant 802825 (M.U.K.); Finnish Association of Cardiothoracic Surgery (K.H.); Finnish Cardiac Society (K.H.); Finnish Foundation for Cardiovascular Research (S.L.K., P.R.M., M.U.K., P.T., and T.K.); Finnish Government Research Funding (K.H.); Finnish Medical Foundation (T.K.); Ida Montin Foundation (P.R.M.); Instrumentarium Science Foundation (P.R.M.); Saastamoinen Foundation (E.S.); Sigrid Jusélius Foundation (S.L.K., M.U.K., and P.T.); Orion Research Foundation (S.L.K., E.S., and K.H.); and Yrjö Jahnsson Foundation (S.L.K. and E.S.). This work was supported in part by NIH grants AG081017, NS129032, NS115064, AG074003, NS127187, AG067151, and HG008155 (M.K.)

AUTHOR CONTRIBUTIONS

Conceptualization, S.L.K.; formal analysis, S.L.K., E.S., J.O., C.A.B., T.Ö., P.R.M., L.H., K.K., and L.Z.A.; methodology, S.L.K., E.S., K.G., T.Ö., A.T., P.K.S., P.R.M., V.L., K.H., A.B., Å.S., E.M., M.H., M.U.K., G.G.C., and T.K.; investigation, S.L.K., E.S., K.G., T.Ö., A.T., P.K.S., P.R.M., V.L., K.H., A.B., Å.S., E.M., M.H., M.U.K., G.G.C., and T.K.; visualization, S.L.K., E.S., C.A.B., T.Ö., P.K.S., V.L., E.M., M.H., M.U.K., G.G.C., and T.K.; resources, S.L.K., K.H., H.K., J.H., J.J., J.G., C.A.M., M.H., J.E.K.H., M.U.K., G.G.C., P.T., T.K., and M.K.; software, S.L.K., E.S., J.O., C.A.B., L.H., and K.K.; funding acquisition, S.L.K., E.S., K.H., P.R.M., M.U.K., P.T., and T.K.; project administration, S.L.K.; supervision, S.L.K., M.U.K., G.G.C., P.T., T.K., and M.K.; writing – original draft, S.L.K., E.S., K.G., T.Ö., M.H., M.U.K., and T.K.; writing – review & editing, S.L.K., J.H., J.E.K.H., G.G.C., P.T., and M.K.

DECLARATION OF INTERESTS

Authors declare no competing interests.

Received: February 2, 2023
Revised: November 27, 2023
Accepted: April 16, 2024
Published: May 21, 2024

REFERENCES

1. Wang, L., Yu, P., Zhou, B., Song, J., Li, Z., Zhang, M., Guo, G., Wang, Y., Chen, X., Han, L., and Hu, S. (2020). Single-cell reconstruction of the adult human heart during heart failure and recovery reveals the cellular landscape underlying cardiac function. Nat. Cell Biol. *22*, 108–119.
2. Litviňuková, M., Talavera-López, C., Maatz, H., Reichart, D., Worth, C.L., Lindberg, E.L., Kanda, M., Polanski, K., Heinig, M., Lee, M., et al. (2020). Cells of the adult human heart. Nature *588*, 466–472.
3. Tucker, N.R., Chaffin, M., Fleming, S.J., Hall, A.W., Parsons, V.A., Bedi, K.C., Jr., Akkad, A.-D., Herndon, C.N., Arduini, A., Papangeli, I., et al. (2020). Transcriptional and cellular diversity of the human heart. Circulation *142*, 466–482.
4. Hocker, J.D., Poirion, O.B., Zhu, F., Buchanan, J., Zhang, K., Chiou, J., Wang, T.-M., Zhang, Q., Hou, X., Li, Y.E., et al. (2021). Cardiac cell

CellPress
OPEN ACCESS

type-specific gene regulatory programs and disease risk association. Sci. Adv. *7*, eabf1444.

5. Nicin, L., Schroeter, S.M., Glaser, S.F., Schulze-Brüning, R., Pham, M.-D., Hille, S.S., Yekelchyk, M., Kattih, B., Abplanalp, W.T., Tombor, L., et al. (2022). A human cell atlas of the pressure-induced hypertrophic heart. Nat. Cardiovasc. Res. *1*, 174–185.
6. Kuppe, C., Ramirez Flores, R.O., Li, Z., Hayat, S., Levinson, R.T., Liao, X., Hannani, M.T., Tanevski, J., Wünnemann, F., Nagai, J.S., et al. (2022). Spatial multi-omic map of human myocardial infarction. Nature *608*, 766–777.
7. Reichart, D., Lindberg, E.L., Maatz, H., Miranda, A., Viveiros, A., Shvetsov, N., Lee, M., Kanemaru, K., Milting, H., Noseda, M., et al. (2022). Pathogenic variants damage cell compositions and single cell transcription in cardiomyopathies. Eur. Heart J. *43*. https://doi.org/10.1093/eurheartj/ehac544.2992.
8. Koenig, A.L., Shchukina, I., Amrute, J., Andhey, P.S., Zaitsev, K., Lai, L., Bajpai, G., Bredemeyer, A., Smith, G., Jones, C., et al. (2022). Single-cell transcriptomics reveals cell-type-specific diversification in human heart failure. Nat. Cardiovasc. Res. *1*, 263–280.
9. Chaffin, M., Papangeli, I., Simonson, B., Akkad, A.-D., Hill, M.C., Arduini, A., Fleming, S.J., Melanson, M., Hayat, S., Kost-Alimova, M., et al. (2022). Single-nucleus profiling of human dilated and hypertrophic cardiomyopathy. Nature *608*, 174–180.
10. Hansen, B.J., Csepe, T.A., and Fedorov, V.V. (2018). Mechanisms of normal and dysfunctional sinoatrial nodal excitability and propagation. In Cardiac Electrophysiology: From Cell to Bedside (Elsevier), pp. 259–271.
11. Wallace, M.J., El Refaey, M., Mesirca, P., Hund, T.J., Mangoni, M.E., and Mohler, P.J. (2021). Genetic complexity of sinoatrial node dysfunction. Front. Genet. *12*, 654925.
12. Kornej, J., Börschel, C.S., Benjamin, E.J., and Schnabel, R.B. (2020). Epidemiology of atrial fibrillation in the 21st century. Circ. Res. *127*, 4–20.
13. Morillo, C.A., Banerjee, A., Perel, P., Wood, D., and Jouven, X. (2017). Atrial fibrillation: the current epidemic. J. Geriatr. Cardiol. *14*, 195–203.
14. Berry, C., and Duncker, D.J. (2020). Coronary microvascular disease: the next frontier for Cardiovascular Research. Cardiovasc. Res. *116*, 737–740.
15. Vancheri, F., Longo, G., Vancheri, S., and Henein, M. (2020). Coronary microvascular dysfunction. J. Clin. Med. *9*, 2880.
16. Kaski, J.-C., Crea, F., Gersh, B.J., and Camici, P.G. (2018). Reappraisal of ischemic heart disease. Circulation *138*, 1463–1480.
17. Godo, S., Suda, A., Takahashi, J., Yasuda, S., and Shimokawa, H. (2021). Coronary microvascular dysfunction. Arterioscler. Thromb. Vasc. Biol. *41*, 1625–1637.
18. Pepine, C.J., Anderson, R.D., Sharaf, B.L., Reis, S.E., Smith, K.M., Handberg, E.M., Johnson, B.D., Sopko, G., and Bairey Merz, C.N. (2010). Coronary microvascular reactivity to adenosine predicts adverse outcome in women evaluated for suspected ischemia. J. Am. Coll. Cardiol. *55*, 2825–2832.
19. Sara, J.D., Widmer, R.J., Matsuzawa, Y., Lennon, R.J., Lerman, L.O., and Lerman, A. (2015). Prevalence of coronary microvascular dysfunction among patients with chest pain and nonobstructive coronary artery disease. JACC Cardiovasc. Interv. *8*, 1445–1453.
20. Hage, C., Svedlund, S., Saraste, A., Faxén, U.L., Benson, L., Fermer, M.L., Gan, L.-M., Shah, S.J., Lam, C.S.P., and Lund, L.H. (2020). Association of coronary microvascular dysfunction with heart failure hospitalizations and mortality in heart failure with preserved ejection fraction: A follow-up in the PROMIS-HFpEF study. J. Card. Fail. *26*, 1016–1021.
21. Radico, F., Zimarino, M., Fulgenzi, F., Ricci, F., Di Nicola, M., Jespersen, L., Chang, S.M., Humphries, K.H., Marzilli, M., and De Caterina, R. (2018). Determinants of long-term clinical outcomes in patients with angina but without obstructive coronary artery disease: a systematic review and meta-analysis. Eur. Heart J. *39*, 2135–2146.
22. Lanza, G.A., Crea, F., and Kaski, J.C. (2019). Clinical outcomes in patients with primary stable microvascular angina: is the jury still out? Eur. Heart J. Qual. Care Clin. Outcomes *5*, 283–291.
23. Camici, P.G., and Crea, F. (2007). Coronary microvascular dysfunction. N. Engl. J. Med. *356*, 830–840.
24. Phan, A., Shufelt, C., and Merz, C.N.B. (2009). Persistent chest pain and no obstructive coronary artery disease. JAMA *301*, 1468–1474.
25. Schmauch, E., Ojanen, J., Galani, K., Jalkanen, J., Hollmen, M., Gunn, J., Kiviniemi, T., Kaikkonen-Määttä, M., Kellis, M., and Linna-Kuosmanen, S. (2022). QClus: Robust and reliable preprocessing method for human heart snRNA-seq. Preprint at bioRxiv. https://doi.org/10.1101/2022.10.21.513315.
26. Kalucka, J., de Rooij, L.P.M.H., Goveia, J., Rohlenova, K., Dumas, S.J., Meta, E., Conchinha, N.V., Taverna, F., Teuwen, L.-A., Veys, K., et al. (2020). Single-cell transcriptome atlas of Murine endothelial cells. Cell *180*, 764–779.e20.
27. Parmar, K.M., Larman, H.B., Dai, G., Zhang, Y., Wang, E.T., Moorthy, S.N., Kratz, J.R., Lin, Z., Jain, M.K., Gimbrone, M.A., Jr., and García-Cardeña, G. (2006). Integration of flow-dependent endothelial phenotypes by Kruppel-like factor 2. J. Clin. Invest. *116*, 49–58.
28. Sangwung, P., Zhou, G., Nayak, L., Chan, E.R., Kumar, S., Kang, D.-W., Zhang, R., Liao, X., Lu, Y., Sugi, K., et al. (2017). KLF2 and KLF4 control endothelial identity and vascular integrity. JCI Insight *2*, e91700.
29. Gimbrone, M.A., Jr., and García-Cardeña, G. (2016). Endothelial cell dysfunction and the pathobiology of atherosclerosis. Circ. Res. *118*, 620–636.
30. He, L., Davila-Velderrain, J., Sumida, T.S., Hafler, D.A., Kellis, M., and Kulminski, A.M. (2021). NEBULA is a fast negative binomial mixed model for differential or co-expression analysis of large-scale multi-subject single-cell data. Commun. Biol. *4*, 629.
31. Krämer, A., Green, J., Pollard, J., Jr., and Tugendreich, S. (2014). Causal analysis approaches in Ingenuity Pathway Analysis. Bioinformatics *30*, 523–530.
32. Sun, C., Tian, X., Jia, Y., Yang, M., Li, Y., and Fernig, D.G. (2022). Functions of exogenous FGF signals in regulation of fibroblast to myofibroblast differentiation and extracellular matrix protein expression. Open Biol. *12*, 210356.
33. Smiljic, S. (2017). The clinical significance of endocardial endothelial dysfunction. Medicina (Kaunas) *53*, 295–302.
34. Rossjohn, J., Gras, S., Miles, J.J., Turner, S.J., Godfrey, D.I., and McCluskey, J. (2015). T cell antigen receptor recognition of antigen-presenting molecules. Annu. Rev. Immunol. *33*, 169–200.
35. Appay, V., and Rowland-Jones, S.L. (2001). RANTES: a versatile and controversial chemokine. Trends Immunol. *22*, 83–87.
36. Sung, M.M., Byrne, N.J., Kim, T.T., Levasseur, J., Masson, G., Boisvenue, J.J., Febbraio, M., and Dyck, J.R.B. (2017). Cardiomyocyte-specific ablation of CD36 accelerates the progression from compensated cardiac hypertrophy to heart failure. Am. J. Physiol. Heart Circ. Physiol. *312*, H552–H560.
37. Dobrzyn, P., Pyrkowska, A., Duda, M.K., Bednarski, T., Maczewski, M., Langfort, J., and Dobrzyn, A. (2013). Expression of lipogenic genes is upregulated in the heart with exercise training-induced but not pressure overload-induced left ventricular hypertrophy. Am. J. Physiol. Endocrinol. Metab. *304*, E1348–E1358.
38. Shu, H., Peng, Y., Hang, W., Nie, J., Zhou, N., and Wang, D.W. (2022). The role of CD36 in cardiovascular disease. Cardiovasc. Res. *118*, 115–129.
39. Chandra, M., Miriyala, S., and Panchatcharam, M. (2017). PPARγ and its role in cardiovascular diseases. PPAR Res. *2017*, 6404638.
40. Altara, R., Manca, M., Hessel, M.H., Gu, Y., van Vark, L.C., Akkerhuis, K.M., Staessen, J.A., Struijker-Boudier, H.A.J., Booz, G.W., and Blankesteijn, W.M. (2016). CXCL10 is a circulating inflammatory marker in

patients with advanced heart failure: A pilot study. J. Cardiovasc. Transl. Res. 9, 302–314.

41. Abbate, A., Toldo, S., Marchetti, C., Kron, J., Van Tassell, B.W., and Dinarello, C.A. (2020). Interleukin-1 and the inflammasome as therapeutic targets in cardiovascular disease. Circ. Res. 126, 1260–1280.
42. Ridker, P.M., Everett, B.M., Thuren, T., MacFadyen, J.G., Chang, W.H., Ballantyne, C., Fonseca, F., Nicolau, J., Koenig, W., Anker, S.D., et al. (2017). Antiinflammatory therapy with canakinumab for atherosclerotic disease. N. Engl. J. Med. 377, 1119–1131.
43. Hickish, T., Andre, T., Wyrwicz, L., Saunders, M., Sarosiek, T., Kocsis, J., Nemecek, R., Rogowski, W., Lesniewski-Kmak, K., Petruzelka, L., et al. (2017). MABp1 as a novel antibody treatment for advanced colorectal cancer: a randomised, double-blind, placebo-controlled, phase 3 study. Lancet Oncol. 18, 192–201.
44. Apte, R.S., Chen, D.S., and Ferrara, N. (2019). VEGF in signaling and disease: Beyond discovery and development. Cell 176, 1248–1264.
45. Gimbrone, M.A., Jr., and García-Cardeña, G. (2013). Vascular endothelium, hemodynamics, and the pathobiology of atherosclerosis. Cardiovasc. Pathol. 22, 9–15.
46. Hayes, J.D., and Dinkova-Kostova, A.T. (2014). The Nrf2 regulatory network provides an interface between redox and intermediary metabolism. Trends Biochem. Sci. 39, 199–218.
47. Popov, L.-D. (2020). Mitochondrial biogenesis: An update. J. Cell Mol. Med. 24, 4892–4899.
48. Dhakshinamoorthy, S., Jain, A.K., Bloom, D.A., and Jaiswal, A.K. (2005). Bach1 competes with Nrf2 leading to negative regulation of the antioxidant response element (ARE)-mediated NAD(P)H:quinone oxidoreductase 1 gene expression and induction in response to antioxidants. J. Biol. Chem. 280, 16891–16900.
49. Nakamura, M., and Sadoshima, J. (2018). Mechanisms of physiological and pathological cardiac hypertrophy. Nat. Rev. Cardiol. 15, 387–407.
50. Dewberry, R., Holden, H., Crossman, D., and Francis, S. (2000). Interleukin-1 receptor antagonist expression in human endothelial cells and atherosclerosis. Arterioscler. Thromb. Vasc. Biol. 20, 2394–2400.
51. Maguire, E.M., Pearce, S.W.A., and Xiao, Q. (2019). Foam cell formation: A new target for fighting atherosclerosis and cardiovascular disease. Vascul. Pharmacol. 112, 54–71.
52. Park, J.-E., Botting, R.A., Domínguez Conde, C., Popescu, D.-M., Lavaert, M., Kunz, D.J., Goh, I., Stephenson, E., Ragazzini, R., Tuck, E., et al. (2020). A cell atlas of human thymic development defines T cell repertoire formation. Science 367, eaay3224.
53. Wirka, R.C., Wagh, D., Paik, D.T., Pjanic, M., Nguyen, T., Miller, C.L., Kundu, R., Nagao, M., Coller, J., Koyano, T.K., et al. (2019). Atheroprotective roles of smooth muscle cell phenotypic modulation and the TCF21 disease gene as revealed by single-cell analysis. Nat. Med. 25, 1280–1289.
54. Zhang, Q., He, Y., Luo, N., Patel, S.J., Han, Y., Gao, R., Modak, M., Carotta, S., Haslinger, C., Kind, D., et al. (2019). Landscape and dynamics of single immune cells in hepatocellular carcinoma. Cell 179, 829–845.e20.
55. Zilionis, R., Engblom, C., Pfirschke, C., Savova, V., Zemmour, D., Saatcioglu, H.D., Krishnan, I., Maroni, G., Meyerovitz, C.V., Kerwin, C.M., et al. (2019). Single-cell transcriptomics of human and mouse lung cancers reveals conserved myeloid populations across individuals and species. Immunity 50, 1317–1334.e10.
56. Eraslan, G., Drokhlyansky, E., Anand, S., Subramanian, A., Fiskin, E., Slyper, M., Wang, J., Wittenberghe, N.V., Rouhana, J.M., Waldman, J., et al. (2021). Single-nucleus cross-tissue molecular reference maps to decipher disease gene function. Preprint at bioRxiv. https://doi.org/10.1101/2021.07.19.452954.
57. van den Hoogen, P., de Jager, S.C.A., Huibers, M.M.H., Schoneveld, A.H., Puspitasari, Y.M., Valstar, G.B., Oerlemans, M.I.F.J., de Weger, R.A., Doevendans, P.A., den Ruijter, H.M., et al. (2019). Increased circulating IgG levels, myocardial immune cells and IgG deposits support a role for an immune response in pre- and end-stage heart failure. J. Cell Mol. Med. 23, 7505–7516.
58. Gerçek, M., Gerçek, M., Kant, S., Simsekyilmaz, S., Kassner, A., Milting, H., Liehn, E.A., Leube, R.E., and Krusche, C.A. (2017). Cardiomyocyte hypertrophy in arrhythmogenic cardiomyopathy. Am. J. Pathol. 187, 752–766.
59. Jaitin, D.A., Adlung, L., Thaiss, C.A., Weiner, A., Li, B., Descamps, H., Lundgren, P., Bleriot, C., Liu, Z., Deczkowska, A., et al. (2019). Lipid-associated macrophages control metabolic homeostasis in a Trem2-dependent manner. Cell 178, 686–698.e14.
60. Fatehi Hassanabad, A., Fedak, P.W.M., and Deniset, J.F. (2021). Acute ischemia alters human pericardial fluid immune cell composition. JACC. Basic Transl. Sci. 6, 765–767.
61. Ashida, N., Arai, H., Yamasaki, M., and Kita, T. (2001). Distinct signaling pathways for MCP-1-dependent integrin activation and chemotaxis. J. Biol. Chem. 276, 16555–16560.
62. Gibaldi, D., Vilar-Pereira, G., Pereira, I.R., Silva, A.A., Barrios, L.C., Ramos, I.P., Mata Dos Santos, H.A., Gazzinelli, R., and Lannes-Vieira, J. (2020). CCL3/macrophage inflammatory protein-1α is dually involved in parasite persistence and induction of a TNF- and IFNγ-enriched inflammatory milieu in Trypanosoma cruzi-induced chronic cardiomyopathy. Front. Immunol. 11, 306.
63. Luster, A.D., Unkeless, J.C., and Ravetch, J.V. (1985). γ-Interferon transcriptionally regulates an early-response gene containing homology to platelet proteins. Nature 315, 672–676.
64. Altara, R., Mallat, Z., Booz, G.W., and Zouein, F.A. (2016). The CXCL10/CXCR3 axis and cardiac inflammation: Implications for immunotherapy to treat infectious and noninfectious diseases of the heart. J. Immunol. Res. 2016, 4396368.
65. Douglas, G., Mehta, V., Al Haj Zen, A., Akoumianakis, I., Goel, A., Rashbrook, V.S., Trelfa, L., Donovan, L., Drydale, E., Chuaiphichai, S., et al. (2020). A key role for the novel coronary artery disease gene JCAD in atherosclerosis via shear stress mechanotransduction. Cardiovasc. Res. 116, 1863–1874.
66. Zhao, C., Zhao, Z., Wang, Z., Hu, L., Wang, H., and Fang, Z. (2020). Supervillin promotes tumor angiogenesis in liver cancer. Oncol. Rep. 44, 674–684.
67. Wang, S., Yu, H., Gao, J., Chen, J., He, P., Zhong, H., Tan, X., Staines, K.A., Macrae, V.E., Fu, X., et al. (2022). PALMD regulates aortic valve calcification via altered glycolysis and NF-κB-mediated inflammation. J. Biol. Chem. 298, 101887.
68. Wang, X., Lu, Y., Xie, Y., Shen, J., and Xiang, M. (2019). Emerging roles of proteoglycans in cardiac remodeling. Int. J. Cardiol. 278, 192–198.
69. van der Harst, P., and Verweij, N. (2018). Identification of 64 novel genetic loci provides an expanded view on the genetic architecture of coronary artery disease. Circ. Res. 122, 433–443.
70. Yan, J., Qiu, Y., Ribeiro Dos Santos, A.M., Yin, Y., Li, Y.E., Vinckier, N., Nariai, N., Benaglio, P., Raman, A., Li, X., et al. (2021). Systematic analysis of binding of transcription factors to noncoding variants. Nature 591, 147–151.
71. Kheradpour, P., and Kellis, M. (2014). Systematic discovery and characterization of regulatory motifs in ENCODE TF binding experiments. Nucleic Acids Res. 42, 2976–2987.
72. Wang, L., Lin, L., Qi, H., Chen, J., and Grossfeld, P. (2022). Endothelial loss of ETS1 impairs coronary vascular development and leads to ventricular non-compaction. Circ. Res. 131, 371–387.
73. King, K.R., Aguirre, A.D., Ye, Y.-X., Sun, Y., Roh, J.D., Ng, R.P., Jr., Kohler, R.H., Arlauckas, S.P., Iwamoto, Y., Savol, A., et al. (2017). IRF3 and type I interferons fuel a fatal response to myocardial infarction. Nat. Med. 23, 1481–1487.
74. Tuomainen, T., and Tavi, P. (2017). The role of cardiac energy metabolism in cardiac hypertrophy and failure. Exp. Cell Res. 360, 12–18.

75. Dinarello, C.A. (2018). Overview of the IL-1 family in innate inflammation and acquired immunity. Immunol. Rev. *281*, 8–27.

76. Ridker, P.M., and Rane, M. (2021). Interleukin-6 signaling and anti-interleukin-6 therapeutics in cardiovascular disease. Circ. Res. *128*, 1728–1746.

77. Ross, R. (1999). Atherosclerosis–an inflammatory disease. N. Engl. J. Med. *340*, 115–126.

78. Ridker, P.M., Cushman, M., Stampfer, M.J., Tracy, R.P., and Hennekens, C.H. (1997). Inflammation, aspirin, and the risk of cardiovascular disease in apparently healthy men. N. Engl. J. Med. *336*, 973–979.

79. Ridker, P.M., Hennekens, C.H., Buring, J.E., and Rifai, N. (2000). C-reactive protein and other markers of inflammation in the prediction of cardiovascular disease in women. N. Engl. J. Med. *342*, 836–843.

80. Zhang, D., Jiang, S.L., Rzewnicki, D., Samols, D., and Kushner, I. (1995). The effect of interleukin-1 on C-reactive protein expression in Hep3B cells is exerted at the transcriptional level. Biochem. J. *310*, 143–148.

81. C Reactive Protein Coronary Heart Disease Genetics Collaboration CCGC; Wensley, F., Gao, P., Burgess, S., Kaptoge, S., Di Angelantonio, E., Shah, T., Engert, J.C., Clarke, R., Davey-Smith, G., et al. (2011). Association between C reactive protein and coronary heart disease: mendelian randomisation analysis based on individual participant data. BMJ *342*, d548.

82. Emerging Risk Factors Collaboration; Kaptoge, S., Di Angelantonio, E., Lowe, G., Pepys, M.B., Thompson, S.G., Collins, R., and Danesh, J. (2010). C-reactive protein concentration and risk of coronary heart disease, stroke, and mortality: an individual participant meta-analysis. Lancet *375*, 132–140.

83. Ridker, P.M., MacFadyen, J.G., Everett, B.M., Libby, P., Thuren, T., Glynn, R.J., P.M., MacFadyen, J.G., Everett, B.M., Libby, P., et al.; CANTOS Trial Group (2018). Relationship of C-reactive protein reduction to cardiovascular event reduction following treatment with canakinumab: a secondary analysis from the CANTOS randomised controlled trial. Lancet *391*, 319–328.

84. Ridker, P.M., Libby, P., MacFadyen, J.G., Thuren, T., Ballantyne, C., Fonseca, F., Koenig, W., Shimokawa, H., Everett, B.M., and Glynn, R.J. (2018). Modulation of the interleukin-6 signalling pathway and incidence rates of atherosclerotic events and all-cause mortality: analyses from the Canakinumab Anti-Inflammatory Thrombosis Outcomes Study (CANTOS). Eur. Heart J. *39*, 3499–3507.

85. Ridker, P.M., Devalaraja, M., Baeres, F.M.M., Engelmann, M.D.M., Hovingh, G.K., Ivkovic, M., Lo, L., Kling, D., Pergola, P., Raj, D., et al. (2021). IL-6 inhibition with ziltivekimab in patients at high atherosclerotic risk (RESCUE): a double-blind, randomised, placebo-controlled, phase 2 trial. Lancet *397*, 2060–2069.

86. Kleveland, O., Kunszt, G., Bratlie, M., Ueland, T., Broch, K., Holte, E., Michelsen, A.E., Bendz, B., Amundsen, B.H., Espevik, T., et al. (2016). Effect of a single dose of the interleukin-6 receptor antagonist tocilizumab on inflammation and troponin T release in patients with non-ST-elevation myocardial infarction: a double-blind, randomized, placebo-controlled phase 2 trial. Eur. Heart J. *37*, 2406–2413.

87. Broch, K., Anstensrud, A.K., Woxholt, S., Sharma, K., Tøllefsen, I.M., Bendz, B., Aakhus, S., Ueland, T., Amundsen, B.H., Damås, J.K., et al. (2021). Randomized trial of interleukin-6 receptor inhibition in patients with acute ST-segment elevation Myocardial Infarction. J. Am. Coll. Cardiol. *77*, 1845–1855.

88. Mountjoy, E., Schmidt, E.M., Carmona, M., Schwartzentruber, J., Peat, G., Miranda, A., Fumis, L., Hayhurst, J., Buniello, A., Karim, M.A., et al. (2021). An open approach to systematically prioritize causal variants and genes at all published human GWAS trait-associated loci. Nat. Genet. *53*, 1527–1533.

89. Li, L., Chen, Z., von Scheidt, M., Li, S., Steiner, A., Güldener, U., Koplev, S., Ma, A., Hao, K., Pan, C., et al. (2022). Transcriptome-wide association study of coronary artery disease identifies novel susceptibility genes. Basic Res. Cardiol. *117*, 6.

90. Erdmann, J., Kessler, T., Munoz Venegas, L., and Schunkert, H. (2018). A decade of genome-wide association studies for coronary artery disease: the challenges ahead. Cardiovasc. Res. *114*, 1241–1257. https://doi.org/10.1093/cvr/cvy084.

91. Wolf, F.A., Angerer, P., and Theis, F.J. (2018). SCANPY: large-scale single-cell gene expression data analysis. Genome Biol. *19*, 15. https://doi.org/10.1186/s13059-017-1382-0.

92. Wolock, S.L., Lopez, R., and Klein, A.M. (2019). Scrublet: Computational identification of cell Doublets in Single-cell transcriptomic data. Cell Syst. *8*, 281–291.e9.

93. Korsunsky, I., Millard, N., Fan, J., Slowikowski, K., Zhang, F., Wei, K., Baglaenko, Y., Brenner, M., Loh, P.-R., and Raychaudhuri, S. (2019). Fast, sensitive and accurate integration of single-cell data with Harmony. Nat. Methods *16*, 1289–1296.

94. McInnes, L., Healy, J., Saul, N., and Großberger, L. (2018). UMAP: Uniform Manifold Approximation and Projection. J. Open Source Softw. *3*, 861.

95. Becht, E., McInnes, L., Healy, J., Dutertre, C.-A., Kwok, I.W.H., Ng, L.G., Ginhoux, F., and Newell, E.W. (2018). Dimensionality reduction for visualizing single-cell data using UMAP. Nat. Biotechnol. *37*, 38–44.

96. Traag, V.A., Waltman, L., and van Eck, N.J. (2019). From Louvain to Leiden: guaranteeing well-connected communities. Sci. Rep. *9*, 5233.

97. Kuleshov, M.V., Jones, M.R., Rouillard, A.D., Fernandez, N.F., Duan, Q., Wang, Z., Koplev, S., Jenkins, S.L., Jagodnik, K.M., Lachmann, A., et al. (2016). Enrichr: a comprehensive gene set enrichment analysis web server 2016 update. Nucleic Acids Res. *44*, W90–W97.

98. Fang, Z., Liu, X., and Peltz, G. (2023). GSEApy: a comprehensive package for performing gene set enrichment analysis in Python. Bioinformatics *39*, btac757. https://doi.org/10.1093/bioinformatics/btac757.

99. Benjamini, Y., and Hochberg, Y. (1995). Controlling the false discovery rate: A practical and powerful approach to multiple testing. J. R. Stat. Soc. *57*, 289–300.

100. Ewels, P.A., Peltzer, A., Fillinger, S., Patel, H., Alneberg, J., Wilm, A., Garcia, M.U., Di Tommaso, P., and Nahnsen, S. (2020). The nf-core framework for community-curated bioinformatics pipelines. Nat. Biotechnol. *38*, 276–278.

101. Dobin, A., Davis, C.A., Schlesinger, F., Drenkow, J., Zaleski, C., Jha, S., Batut, P., Chaisson, M., and Gingeras, T.R. (2013). STAR: ultrafast universal RNA-seq aligner. Bioinformatics *29*, 15–21.

102. Patro, R., Duggal, G., Love, M.I., Irizarry, R.A., and Kingsford, C. (2017). Salmon provides fast and bias-aware quantification of transcript expression. Nat. Methods *14*, 417–419.

103. Love, M.I., Huber, W., and Anders, S. (2014). Moderated estimation of fold change and dispersion for RNA-seq data with DESeq2. Genome Biol. *15*, 550.

104. Heinz, S., Benner, C., Spann, N., Bertolino, E., Lin, Y.C., Laslo, P., Cheng, J.X., Murre, C., Singh, H., and Glass, C.K. (2010). Simple combinations of lineage-determining transcription factors prime cis-regulatory elements required for macrophage and B cell identities. Mol. Cell *38*, 576–589.

105. Ritchie, M.E., Phipson, B., Wu, D., Hu, Y., Law, C.W., Shi, W., and Smyth, G.K. (2015). limma powers differential expression analyses for RNA-sequencing and microarray studies. Nucleic Acids Res. *43*, e47.

106. Palla, G., Spitzer, H., Klein, M., Fischer, D., Schaar, A.C., Kuemmerle, L.B., Rybakov, S., Ibarra, I.L., Holmberg, O., Virshup, I., et al. (2022). Squidpy: a scalable framework for spatial omics analysis. Nat. Methods *19*, 171–178.

107. Kuosmanen, S.M., Hartikainen, J., Hippeläinen, M., Kokki, H., Levonen, A.-L., and Tavi, P. (2015). MicroRNA profiling of pericardial fluid samples from patients with heart failure. PLoS One *10*, e0119646.

108. Neumann, F.-J., Sousa-Uva, M., Ahlsson, A., Alfonso, F., Banning, A.P., Benedetto, U., Byrne, R.A., Collet, J.-P., Falk, V., Head, S.J., et al. (2019). 2018 ESC/EACTS Guidelines on myocardial revascularization. Eur. Heart J. *40*, 87–165.

109. Lawton, J.S., Tamis-Holland, J.E., Bangalore, S., Bates, E.R., Beckie, T.M., Bischoff, J.M., Bittl, J.A., Cohen, M.G., DiMaio, J.M., Don, C.W., et al. (2022). 2021 ACC/AHA/SCAI guideline for coronary artery revascularization: A report of the American college of cardiology/American heart association joint committee on clinical practice guidelines. Circulation *145*, e18–e114.

110. Ponikowski, P., Voors, A.A., Anker, S.D., Bueno, H., Cleland, J.G.F., Coats, A.J.S., Falk, V., González-Juanatey, J.R., Harjola, V.-P., Jankowska, E.A., et al. (2016). 2016 ESC Guidelines for the diagnosis and treatment of acute and chronic heart failure. Eur. Heart J. *37*, 2129–2200.

111. Hollenberg, S.M., Warner Stevenson, L., Ahmad, T., Amin, V.J., Bozkurt, B., Butler, J., Davis, L.L., Drazner, M.H., Kirkpatrick, J.N., Peterson, P.N., et al. (2019). 2019 ACC expert consensus decision pathway on risk assessment, management, and clinical trajectory of patients hospitalized with heart failure: A report of the American college of cardiology solution set oversight committee. J. Am. Coll. Cardiol. *74*, 1966–2011.

112. Howe, K.L., Achuthan, P., Allen, J., Allen, J., Alvarez-Jarreta, J., Amode, M.R., Armean, I.M., Azov, A.G., Bennett, R., Bhai, J., et al. (2021). Ensembl 2021. Nucleic Acids Res. *49*, D884–D891.

113. Chistiakov, D.A., Killingsworth, M.C., Myasoedova, V.A., Orekhov, A.N., and Bobryshev, Y.V. (2017). CD68/macrosialin: not just a histochemical marker. Lab. Invest. *97*, 4–13.

114. Betjes, M.G., Haks, M.C., Tuk, C.W., and Beelen, R.H. (1991). Monoclonal antibody EBM11 (anti-CD68) discriminates between dendritic cells and macrophages after short-term culture. Immunobiology *183*, 79–87.

115. Murray, P.J., and Wynn, T.A. (2011). Protective and pathogenic functions of macrophage subsets. Nat. Rev. Immunol. *11*, 723–737.

116. Muerdter, F., Boryń, Ł.M., Woodfin, A.R., Neumayr, C., Rath, M., Zabidi, M.A., Pagani, M., Haberle, V., Kazmar, T., Catarino, R.R., et al. (2018). Resolving systematic errors in widely used enhancer activity assays in human cells. Nat. Methods *15*, 141–149.

117. Toropainen, A., Stolze, L.K., Örd, T., Whalen, M.B., Torrell, P.M., Link, V.M., Kaikkonen, M.U., and Romanoski, C.E. (2022). Functional noncoding SNPs in human endothelial cells fine-map vascular trait associations. Genome Res. *32*, 409–424.

118. Örd, T., Õunap, K., Stolze, L.K., Aherrahrou, R., Nurminen, V., Toropainen, A., Selvarajan, I., Lönnberg, T., Aavik, E., Ylä-Herttuala, S., et al. (2021). Single-cell epigenomics and functional fine-mapping of atherosclerosis GWAS loci. Circ. Res. *129*, 240–258.

119. ENCODE Project Consortium (2012). An integrated encyclopedia of DNA elements in the human genome. Nature *489*, 57–74.

120. Sha, Y., Phan, J.H., and Wang, M.D. (2015). Effect of low-expression gene filtering on detection of differentially expressed genes in RNA-seq data. In 2015 37th Annual International Conference of the IEEE Engineering in Medicine and Biology Society (EMBC) (IEEE)). https://doi.org/10.1109/embc.2015.7319872.

121. Nelson, C.P., Goel, A., Butterworth, A.S., Kanoni, S., Webb, T.R., Marouli, E., Zeng, L., Ntalla, I., Lai, F.Y., Hopewell, J.C., et al. (2017). Association analyses based on false discovery rate implicate new loci for coronary artery disease. Nat. Genet. *49*, 1385–1391.

122. Malik, R., Chauhan, G., Traylor, M., Sargurupremraj, M., Okada, Y., Mishra, A., Rutten-Jacobs, L., Giese, A.-K., van der Laan, S.W., Gretarsdottir, S., et al. (2018). Multiancestry genome-wide association study of 520,000 subjects identifies 32 loci associated with stroke and stroke subtypes. Nat. Genet. *50*, 524–537.

123. Kichaev, G., Bhatia, G., Loh, P.-R., Gazal, S., Burch, K., Freund, M.K., Schoech, A., Pasaniuc, B., and Price, A.L. (2019). Leveraging polygenic functional enrichment to improve GWAS power. Am. J. Hum. Genet. *104*, 65–75.

124. Matsunaga, H., Ito, K., Akiyama, M., Takahashi, A., Koyama, S., Nomura, S., Ieki, H., Ozaki, K., Onouchi, Y., Sakaue, S., et al. (2020). Transethnic meta-analysis of genome-wide association studies identifies three new loci and characterizes population-specific differences for coronary artery disease. Circ. Genom. Precis. Med. *13*, e002670.

125. Koyama, S., Ito, K., Terao, C., Akiyama, M., Horikoshi, M., Momozawa, Y., Matsunaga, H., Ieki, H., Ozaki, K., Onouchi, Y., et al. (2020). Population-specific and trans-ancestry genome-wide analyses identify distinct and shared genetic risk loci for coronary artery disease. Nat. Genet. *52*, 1169–1177.

126. Hartiala, J.A., Han, Y., Jia, Q., Hilser, J.R., Huang, P., Gukasyan, J., Schwartzman, W.S., Cai, Z., Biswas, S., Trégouët, D.A., et al. (2021). Genome-wide analysis identifies novel susceptibility loci for myocardial infarction. Eur. Heart J. *42*, 919–933.

127. Kurki, M.I., Karjalainen, J., Palta, P., Sipilä, T.P., Kristiansson, K., Donner, K., Reeve, M.P., Laivuori, H., Aavikko, M., Kaunisto, M.A., et al. (2022). FinnGen: Unique genetic insights from combining isolated population and national health register data. Preprint at bioRxiv. https://doi.org/10.1101/2022.03.03.22271360.

128. Zhou, W., Nielsen, J.B., Fritsche, L.G., Dey, R., Gabrielsen, M.E., Wolford, B.N., LeFaive, J., VandeHaar, P., Gagliano, S.A., Gifford, A., et al. (2018). Efficiently controlling for case-control imbalance and sample relatedness in large-scale genetic association studies. Nat. Genet. *50*, 1335–1341.

129. Aragam, K.G., Jiang, T., Goel, A., Kanoni, S., Wolford, B.N., Weeks, E.M., Wang, M., Hindy, G., Zhou, W., Grace, C., et al. (2021). Discovery and systematic characterization of risk variants and genes for coronary artery disease in over a million participants. Preprint at bioRxiv. https://doi.org/10.1101/2021.05.24.21257377.

STAR★METHODS

KEY RESOURCES TABLE

REAGENT or RESOURCE	SOURCE	IDENTIFIER
Antibodies		
Anti-CD68 antibody	Abcam	cat#ab845
anti-TREM2 antibody	Novus Biologicals	Clone#37920
Bacterial and virus strains		
lentiviral vector lenti_dCas9-KRAB-MeCP2	Andrea Califano	# 122205; http://n2t.net/addgene:122205; RRID: Addgene_122205
pSPgRNA vector	Charles Gersbach	Addgene plasmid # 47108; http://n2t.net/addgene:47108; RRID: Addgene_47108
Biological samples		
PERIHEART right atrial appendage samples	PERIHEART	N/A
PERIHEART pericardial fluid samples	PERIHEART	N/A
CAREBANK right atrial appendage samples	CAREBANK	ClinicalTrials.gov Identifier: NCT03444259
CAREBANK serum samples	CAREBANK	ClinicalTrials.gov Identifier: NCT03444259
Chemicals, peptides, and recombinant proteins		
IL-1β 10 ng/ml	GIBCO	Cat#PHC0814
Hematoxylin	Vector Laboratories	Cat#H-3401-500
Eosin	Sigma-Aldrich	Cat#HT110116
EcoMount mounting medium	BioCare Medical	Cat#EM897L
EGM; basal medium	Lonza	Cat#CC-4133
Fetal bovine serum (FBS)	GIBCO	Cat#A3840001
ACK lysing buffer	GIBCO, Thermo Fisher Scientific, Inc	Cat#A1049201
RNase inhibitors	TaKaRa	Cat#2313A
EDTA-free protease inhibitor cocktail	Roche	Cat#11873580001
BODIPY^{TM} 493/503 (4,4-Difluoro-1,3,5,7,8-Pentamethyl-4-Bora-3a,4a-Diaza-*s*-Indacene)	ThermoFisher	Cat#D3922
Vascular Cell Basal Medium	ATCC	Cat#PCS-100-030
Vascular Endothelial Cell Growth Kit-VEGF	ATCC	Cat#PCS-100-041
Resuspension buffer R	Thermo Fisher Sci.	Cat# MPK1096
Quick-DNA/RNA Miniprep Plus Kit	Zymo	Cat#D7003
Blasticidin S HCL	Corning, Fisher Scientific	Cat#15383671
Monarch® Total RNA Miniprep Kit	NEB	Cat#T2010
Critical commercial assays		
Chromium Single Cell 3' GEM, Library & Gel Bead Kit v3, 16 rxns	10x Genomics	Cat#PN-1000075
Dual Index kit TT set A	10x Genomics	Cat#PN-1000215
NovaSeq 6000 S2 System	Illumina	N/A
RNeasy Mini Kit	QIAGEN	Cat#74104
Molecular Cartography^{TM} spatial transcriptomics	Resolve Bioscience	N/A
Visium Spatial Gene Expression Slide & Reagent Kit, 16 rxns	10x Genomics	Cat#PN-1000184
Visium Spatial Tissue Optimization Slide & Reagent Kit, 4 slides	10x Genomics	Cat#PN-1000193
NextSeq 500 system (150 cycles)	Illumina	N/A

(*Continued on next page*)

Continued		
REAGENT or RESOURCE	SOURCE	IDENTIFIER
Bio-Plex Pro Human Cytokine Screening Panel	Bio-rad	Cat#12007283
10μL Neon Transfection System kit	Thermo Fisher Sci.	Cat#MPK1025
Deposited data		
OpenTargets Genetics Portal (data release: June 2021)	Mountjoy, E. et al.[88]	https://genetics.opentargets.org/
Human reference genome NCBI build 38, GRCh38	Genome Reference Consortium	http://www.ncbi.nlm.nih.gov/projects/genome/assembly/grc/human/
TWAS of CAD	by Li *et al.*[89]	N/A
CAD GWAS review	Erdmann et al.[90]	N/A
snRNA and spatial transcriptomics data	This paper	10.5281/zenodo.10822323
Experimental models: Cell lines		
HUVECs	This paper	N/A
HAEC	LONZA	N/A
Primary human aortic smooth muscle cells	Thermo Scientific	N/A
TeloHAECs	This paper	N/A
Oligonucleotides		
JCAD1_FWD: GCACTTCCT CCTGCCATAAA	This paper	N/A
JCAD1_REV: ACACCCAACAT CCCTGTATTC	This paper	N/A
JCAD2_FWD: CCTCTTTGCCT ACTTCCTCTTAC	This paper	N/A
JCAD2_REV: GTGGAACCCTC ATTACCTCATC	This paper	N/A
FH2_KIAA1462: AACAATGACTT AAAGCCCAG	This paper	N/A
BH2_KIAA1462: ACTGAGGTCA TTTGTTTGTC	This paper	N/A
FH1_GAPDH: TCGGAGTCAACGGATTTG	This paper	N/A
BH1_GAPDH: CAACAATATCCA CTTTACCAGAG	This paper	N/A
Software and algorithms		
Cell Ranger v6.0	10x Genomics	https://www.10xgenomics.com/support/software/cell-ranger/latest
Scanpy v1.7	Wolf, F. A. et al.[91]	https://scanpy.readthedocs.io/en/latest/_tutorials.html
python v3.9	Python Software Foundation	https://www.python.org/
QClus	Schmauch E. et al.[25]	https://github.com/scHEARTGROUP/qclus
Scrublet	Wolock, S. L. et al.[92]	https://github.com/swolock/scrublet
Harmony	Korsunsky, I. et al.[93]	https://github.com/slowkow/harmonypy
UMAP	McInnes, L. et al.[94] Becht, E. et al.[95]	N/A
Leiden	Traag, V. A. et al.[96]	N/A
Enrichr	Kuleshov, M. V. et al.[97]	https://maayanlab.cloud/Enrichr/
gseapy	Fang, Z. et al.[98]	https://gseapy.readthedocs.io/en/latest/introduction.html
Benjamini-Hochberg	Benjamini, Y. & Hochberg, Y.[99]	N/A
Nebula	He, L. et al.[30]	https://github.com/lhe17/nebula
Nf-core RNA-seq pipeline	Ewels, P. A. et al.[100]	https://nf-co.re/rnaseq/3.7.0
STAR	Dobin, A. et al.[101]	N/A

(*Continued on next page*)

Continued

REAGENT or RESOURCE	SOURCE	IDENTIFIER
Salmon	Patro, R. et al.[102]	N/A
Deseq2	Love, M. I. et al.[103]	https://bioconductor.org/packages/release/bioc/html/DESeq2.html
HOMER v4.9	Heinz, S. et al.[104]	http://homer.ucsd.edu/homer/
Limma v3.46.0	Ritchie, M. E. et al.[105]	https://bioconductor.org/packages/release/bioc/html/limma.html
IPA	QIAGEN, Krämer, A. et al.[31]	https://digitalinsights.qiagen.com/IPA
Venny	Juan Carlos Oliveros BioinfoGP	https://bioinfogp.cnb.csic.es/tools/venny/index.html
Squidpy	Palla, G. et al.[106]	https://squidpy.readthedocs.io/en/stable/notebooks/tutorials/index.html
**Other**		
Nikon DX-DB digital camera	Nikon	N/A
Nikon Digital Sight DS-U1 microscope	Nikon	N/A
Leica DMi8 microscope	Leica	N/A
Pannoramic 250 digital slide scanner	3DHISTECH	N/A

RESOURCE AVAILABILITY

Lead contact

Further information and requests for resources and reagents should be directed to and will be fulfilled by the lead contact, Suvi Linna Kuosmanen (suvi.linna-kuosmanen@uef.fi).

Materials availability

This study did not generate new unique reagents.

Data and code availability

- Processed data, raw counts, annotations, and images have been deposited in Zenodo (https://doi.org/10.5281/zenodo.10822323).
- Data analysis was performed as described in the methods, according to pipeline and code described in the used packages tutorials. These can be found at https://scanpy-tutorials.readthedocs.io/en/latest/index.html, https://squidpy.readthedocs.io/en/stable/notebooks/tutorials/index.html, https://github.com/lhe17/nebula, https://nf-co.re/rnaseq/3.7.0/docs/usage, https://www.bioconductor.org/packages/release/bioc/vignettes/DESeq2/inst/doc/DESeq2.html, and https://gseapy.readthedocs.io/en/latest/introduction.html.
- The code and usage of the QClus droplet filtering method used in this paper can be found at https://github.com/scHEARTGROUP/qclus. Detailed description of the method and its benchmarking are available in the method preprint (https://doi.org/10.1101/2022.10.21.513315).
- Any additional information required to reanalyze the data reported in this work paper is available from the lead contact upon request.

EXPERIMENTAL MODEL AND STUDY PARTICIPANT DETAILS

Human biopsies - sample selection

Human right atrial appendage samples were harvested as a part of the ongoing prospective PERIHEART and CAREBANK studies (ClinicalTrials.gov Identifier: NCT03444259). Ethical Committees of the Hospital Districts of Southwest Finland and Northern Savo approved the protocol and study complies with the Declaration of Helsinki as revised in 2002. The CAREBANK study (Table S2A) has enrolled patients undergoing open-heart cardiac surgery (coronary bypass surgery, operations for valvular heart disease and ascending aorta) since February 2016 at Turku University Hospital and the PERIHEART study (Table S2B) since 2012 at Kuopio University Hospital. All patients gave their written informed consent prior to surgery.

Clinical data collection was performed in both collections prospectively as electronic case reports with structured questionnaires by a trained research nurse. Pericardial fluid was collected in the beginning of the surgery by aspirating to a syringe. Pericardial fluid samples were processed as described in Kuosmanen *et al.*[107] and all tubes were snap-frozen and stored at −80°C. Tissue samples were taken from the right atrial appendage, from the anatomical site of cardiopulmonary bypass venous cannulation. Samples were always obtained in the beginning of the operation immediately before patient was coupled to cardiopulmonary bypass to avoid the

effects of cardiopulmonary bypass time or technical surgery type on the sample quality. Every sample contained all three layers of the heart wall (epicardium, myocardium, and endocardium). Tissue samples were snap-frozen immediately upon collection and stored at −80°C.

In the CAREBANK samples, control individuals were operated due to valvular heart disease, had no other cardiovascular disease, and their hearts appeared otherwise healthy (preventive surgery). Patients with ischemic heart disease (IHD) had an indication for coronary revascularization as appropriate by the current guidelines.[108,109] Ischemic heart failure (IHF) was defined as reduced left ventricular ejection fraction below 50% in echocardiography in addition to above criteria for revascularization.[110,111] Non-ischemic heart failure (NIHF) was defined as reduced left ventricular ejection fraction below 50% without coronary artery disease in preoperative coronary angiogram. All patients in control and NIHF groups had valvular disease but no coronary artery disease, and both IHD and IHF included patients with valvular disease.

In the PERIHEART study, control individuals were operated due to mitral valve regurgitation, had no other significant cardiovascular disease, and their hearts appeared otherwise healthy. In IHD groups, stable coronary artery disease (stable CAD) consisted of patients with significant coronary artery disease who underwent elective Coronary Artery Bypass Grafting (CABG) surgery[108,109]; patients with acute myocardial infarction (acute MI) had preoperative myocardial infarction and were operated with CABG during the same hospital stay; and patients with remote myocardial infarction (remote MI) had suffered myocardial infarction in the past but not prior to CABG surgery.

Endothelial cells

For the disturbed flow experiment, Human umbilical vein endothelial cells (HUVECs) were isolated and cultured as previously described.[27] For the IL-1β stimulation experiment, human aortic endothelial cells (HAEC)(LONZA) were maintained in endothelial cell growth medium (EGM; basal medium with SingleQuots supplements CC-4133; Lonza) supplemented with 10% fetal bovine serum (FBS; GIBCO) on T-75 cell culture flasks coated with 10 g/mL fibronectin (Sigma, St Louis, MO, USA) and 0.05% gelatin at 37°C in a humidified atmosphere at 5% CO2 and used at passage 4 to 6.

METHOD DETAILS

Histochemistry

Tissues were embedded in Optimal cutting temperature (OCT) compound and stored at −80°C. OCT-embedded tissue blocks were sectioned at a thickness of 10 microns. Frozen tissue sections were air-dried at room temperature and then fixed in 4% paraformaldehyde in PBS solution for 10 min. Fixed tissue sections were stained with Hematoxylin (Vector Laboratories, Burlingame, CA) for 10 min. After several washes in distilled water, sections were then stained with 0.5% Eosin (Sigma-Aldrich, St. Louis, MO) for 10 s followed by several washes in distilled water. Sections were then dehydrated by sequentially dipping 10 times in 50%, 70%, 95% and 100% ethanol respectively. Slides were completely air-dried and then cleared by dipping in xylene for 10s. Stained tissue sections were mounted using EcoMount mounting medium (Biocare Medical, Pacheco, CA) and images were captured with a Nikon DX-DB digital camera using a Nikon Digital Sight DS-U1 microscope.

Isolation of nuclei from *ex vivo* cardiac tissue and pericardial fluid

Pericardial fluid was processed using a three-step centrifugation protocol as described in Kuosmanen *et al.,*[107] and the cell pellets were flash-frozen immediately after collection. For nuclei isolation, the frozen pellet was thawed for 3 min in a water bath at 37°C, spun down and treated with 0.5 mL ACK lysing buffer (Gibco, Thermo Fisher Scientific, Inc) for 5 min at RT. Cells were centrifuged for 5 min, at 300 x g at 4°C, the supernatant was removed, and the cleared pellet was lysed in 0.1 mL lysis buffer (10 mM Tris-HCl (pH 7.4), 10 mM NaCl, 3 mM MgCl, 0.1% Tween 20, 0.1% NP40, 1% BSA, 1 mM DDT, 1 U/μL Rnase inhibitors (TaKaRa)). Lysis progression was monitored under the microscope and stopped as soon as 95–98% of cells were lysed with the addition of 1 mL ice-cold wash buffer (10 mM Tris-HCl (pH 7.4), 10 mM NaCl, 3 mM MgCl, 0.1% Tween 20, 1% BSA, 1 mM DDT, 1 U/ul Rnase inhibitors (TaKaRa)). Nuclei were spun down for 5 min, at 500 x g, 4°C, resuspended in 0.04% BSA in PBS, stained with Trypan Blue, counted on a hemocytometer, and adjusted to a concentration of 1000 nuclei/μL.

For tissue samples in OCT, surrounding OCT was removed with a scalpel and the tissue was washed three times with ice-cold 0.04% BSA in PBS. For all tissue samples, the tissue was transferred into a tube containing 1 mL of ice-cold lysis buffer (0.32 M Sucrose, 5 mM $CaCl_2$, 3mM MgAc, 2.0 mM EDTA, 0.5mM EGTA, 10 mM Tris-HCl (pH 8.0), 1 mM DDT, EDTA-free protease inhibitor cocktail (Roche, Complete tablet), 1 U/μL RNase inhibitors (TaKaRa)), cut into 2–3 mm pieces with scissors, transferred to an ice-cold glass dounce tissue grinder and after 5 min of incubation on ice, stroked with a "Loose" and a "Tight" pestle, 15 times each. The solution was passed through a 40-micron strainer and centrifuged at 1000 x g for 8 min at 4°C on a swinging bucket rotor. The supernatant was removed, and the nuclear pellet was resuspended in 1 mL of nuclei suspension buffer (0.04% BSA in PBS with RNase inhibitors) and centrifuged at 1000 x g for 8 min at 4°C on a swinging bucket rotor. After removal of the supernatant, nuclear pellet was resuspended in the remaining nuclei suspension buffer. The quality of the nuclear suspension was estimated under the microscope following staining with Trypan Blue and nuclei concentration was adjusted to 1000 nuclei/μL.

Droplet-based snRNA-seq

Immediately after nuclei isolation, single-nuclei RNA (snRNA) libraries were processed using the droplet-based RNA sequencing technology. Briefly, 5000–7000 nuclei were profiled per sample using the Chromium Single Cell 3′ RNA reagent kit v3 according to the 10X Genomics protocol. The generated cDNA libraries were indexed, pooled, and sequenced using the NovaSeq 6000 S2 system and reagent kits (100 cycles) (Illumina, Inc).

Endothelial cells – Disturbed flow

HUVECS were then exposed to disturbed, non-laminar flow or left under static (no flow) conditions for 24h. At the end of the experiments, cells were lysed, and mRNA was isolated and used for NGS library preparation and sequencing at the Broad Institute.

Endothelial cells – IL-1β stimulation

Prior to IL-1β stimulation, HAEC medium was changed with fresh endothelial cell growth medium (EGM; basal medium; Lonza) supplemented with 1% fetal bovine serum (FBS; GIBCO). HAEC were then stimulated with 10 ng/mL of IL-1β (PHC0814, GIBCO) at 2, 8, and 23h time points. RNA was extracted using RNeasy Mini Kit (74104, QIAGEN) according to manufacturer protocol. Extracted RNA was sent to the Sequencing Service GeneCore Sequencing Facility (EMBL, www.genecore.embl.de) for NGS library preparation using stranded rRNA-depleted RNA-Seq protocol.

Resolve molecular cartography

Right atrial appendage biopsy fresh-frozen tissue was sectioned and transferred to capture areas on spatial transcriptomics slides by Resolve Bioscience and processed using their Molecular Cartography spatial transcriptomics. One hundred genes were measured at a time. For each gene, all transcripts for ENSEMBL[112] were utilized to ensure that they were all counted. Samples were primed and hybridized with all probes which were then fluorescently tagged. Regions of interest were imaged and decolorized to remove fluorescent signals. Development of color, imaging and decolorization steps were repeated several times in cycles to create a combination of fluorescence, which was decoded to retrieve the gene information from the images.

Visium spatial transcriptomics

One heart failure and one control sample were processed, and four technical replicates were measured for each sample. Thin (10μm) sections of OCT-embedded tissue were cryosectioned at −20°C on a cryostat, mounted on tissue optimization (TO) and gene expression (GEX) Visium slides and stored at −80°C for up to one week. TO slides were used to determine the optimum tissue permeabilization time following the protocol of the manufacturer (Visium Spatial TO Slide and Reagent kit, 10x Genomics, Pleasanton, CA). Briefly, sections were methanol-fixed, stained with hematoxylin and eosin (H&E stain), and imaged on a Leica DMi8 microscope. Next, a permeabilization time course was performed, followed by steps of fluorescent cDNA synthesis, tissue removal, and fluorescent imaging. GEX slides were similarly fixed, H&E stained, and imaged on a Leica DMi8 microscope. Next, the tissue was permeabilized according to the TO-determined permeabilization time and four cDNA libraries were prepared according to the manufacturer protocol (Visium Spatial GEX Slide and Reagent kit, 10x Genomics, Pleasanton, CA). Each set of 4 libraries was pooled and sequenced using the NextSeq 500 system (150 cycles) (Illumina, Inc).

Cytokine screening from serum samples

Patient samples were selected from CAREBANK study (ClinicalTrials.gov Identifier: NCT03444259). Serum samples were collected preoperatively after at least 8h of fasting by routine sampling of the certified hospital laboratory. The serum samples were stored at −70°C until analysis. Serum cytokine screening was done using the Bio-Plex Pro Human Cytokine Screening Panel, 48-Plex (cat#: 12007283, Bio-rad) according to the instructions of the manufacturer.

Immunohistochemistry

For immunostaining, OCT blocks were sectioned at a thickness of 5 μm. Standard immunostaining protocols were used. Anti-CD68 antibody (prediluted, cat#: ab845, Abcam) was used as a common macrophage marker, anti-TREM2 antibody (clone: 237920, Novus Biologicals) for identifying LAMs, and BODIPY 493/503 (4,4-Difluoro-1,3,5,7,8-Pentamethyl-4-Bora-3a,4a-Diaza-*s*-Indacene; cat#: D3922, ThermoFisher) for staining lipids.[113–115] Stained slides were scanned using Pannoramic 250 digital slide scanner. Criteria for macrophages included morphology and granular inclusions in the cytoplasm. For each subject the same sized randomly selected areas from pericardium were evaluated. Images were analyzed using ImageJ2/Fiji. Number of CD68 positive macrophages were divided by total nuclei count.

Allelic activity reporter assay

STARR-Seq massively parallel reporter assay[116] was used to compare the transcriptional activity of SNP alleles in teloHAEC human immortalized aortic endothelial cells (ATCC) and in primary human aortic smooth muscle cells (Thermo Scientific). The library generation, quality control and analysis has been described in detail in Toropainen *et al.*[117] and Örd *et al.*,[118] respectively.

CRISPR-RNP transfection

The human endothelial cells, TeloHAECs were maintained in Vascular Cell Basal Medium (ATCC PCS-100-030), supplemented with Vascular Endothelial Cell Growth Kit-VEGF (ATCC PCS-100-041) and 100 I.U./mL penicillin- 100 (μg/mL) streptomycin. The cells were grown to 70–90% confluency before the experiment. The CRISPR deletion experiment was performed as per standard protocol provided by IDT. Briefly, Alt-R CRISPR-Cas9 crRNA/gRNA, and tracrRNA were dissolved in duplex buffer, and Alt-R CRISPR-Cas9 Electroporation Enhancer (EE) in IDTE, pH 8.0 to a final concentration of 100 μM. The crRNA:tracrRNA duplexes were formed by mixing two oligos at equimolar concentrations in a sterile microcentrifuge, then heated at 95°C for 5 min. For each electroporation reaction, 36μM of Alt-R S.p. HiFi Cas9 enzyme and crRNA:tracrRNA duplexes were incubated together to form the ribonucleoprotein (RNP) complexes following the manufacturer guidelines. The cells were then washed, trypsinized, and re-washed with warm PBS before preparing the working cell stock in resuspension buffer R (Thermo Fisher Sci. Cat# MPK1096). 50 000 cells were used per 10μL of reaction volume, and three reactions were performed for each well of a 12-well plate. Finally, cells, specific RNP complex, and 10.8 μM of Alt-R Cas9 electroporation enhancer were mixed before loading into 10μL Neon Transfection System kit. The electroporation was performed using voltage: 1350V, width: 30ms, pulses: 1 pulse. The newly transfected cells were inoculated immediately to pre-warmed complete cell culture media and allowed to grow for 48h before collection. The genomic DNA and RNA were collected from same sample using Quick-DNA/RNA Miniprep Plus Kit (Zymo, Cat D7003). To confirm the CRISPR-RNP deletion of the specific target regions, we performed PCR followed by agarose gel electrophoresis using genomic DNA and target specific primer pairs (JCAD1_FWD: GCACTTCCTCCTGCCATAAA and JCAD1_REV: ACACCCAACATCCCTGTATTC; JCAD2_FWD: CCTCTTTGCCTACTTCCTCTTAC and JCAD2_REV: GTGGAACCCTCATTACCTCATC. Then, we performed qPCR using ΔΔCt method to measure the relative gene expression of JCAD/KIAA1462 among collected RNA samples using GAPDH as the house keeping gene to normalize between control and treatments. We used the following primer pairs for qPCR: FH2_KIAA1462: AACAATGACTTAAAGCCCAG and BH2_KIAA1462: ACTGAGGTCATTTGTTTGTC; FH1_GAPDH: TCGGAGTCAACGGATTTG and BH1_GAPDH: CAACAATATCCACTTTACCAGAG.

CRISPRi: Plasmid transfection

To perform the CRISPR-based gene silencing, we developed Telo-KRAB: TeloHAEC stably expressing dCAS9-KRAB using a lentivirus-based approach. The lentiviral vector lenti_dCas9-KRAB-MeCP2 was a gift from Andrea Califano (Addgene plasmid # 122205; http://n2t.net/addgene:122205; RRID: Addgene_122205). The viral vector was transduced to teloHAEC cells at MOI 20, screened by Blasticidin S HCL (Corning, Fisher Scientific# 15383671) selection (6 μg/mL) and stored in liquid nitrogen for future use. The guide RNAs were cloned into the pSPgRNA vector (pSPgRNA was a gift from Charles Gersbach, Addgene plasmid # 47108; http://n2t.net/addgene:47108; RRID: Addgene_47108) following standard protocol. A total of five gRNAs for region 2 (JCAD 2) and three gRNAs for JCAD 1 were cloned into the pSPgRNA vector and validated by restriction digestions and sanger sequencing. The Telo-KRAb cells were grown to 70–90% confluency before the transfection. The gRNA plasmids were mixed as pairs: one plasmid targeted the upstream, and other targeted the downstream region of the SNP of interest. The control GFP plasmid (10% w/w) was also added to each combination to visualize the transfection efficiency microscopically. The cells were washed, trypsinized, and re-washed before the transfection reaction. A total of 50 000 cells were loaded along with plasmid mixture into each 10 μL tip of the Neon Transfection System kit. The optimized reaction condition used for Telo-KRAB cells was voltage: 1350V, width: 30ms, pulses: 1 pulse. Three electroporation reactions were performed for each treatment, and the cells were inoculated into a 12-well plate immediately after transfection. The cells were then grown for 24h to check the GFP expression as the sign of transfection efficiency. The samples for each reaction were collected after 48h of post-transfection. The RNA extraction was performed using Monarch Total RNA Miniprep Kit. (NEB, cat T2010). To measure the extent of gene silencing, qPCR was performed for GAPDH and JCAD/KIAA1462 as described in 'CRISPR-RNP transfection'.

QUANTIFICATION AND STATISTICAL ANALYSIS

Bioinformatics preprocessing of snRNA-seq samples

The samples were processed using Cell Ranger v6.0 (10x Genomics) and the data were analyzed using Scanpy v1.7[91] on python v3.9. To remove empty droplets and highly contaminated cells, we used a method, that we developed for cardiac tissue, QClus.[25] To summarize, QClus computes contamination-related quality metrics, i.e., splicing fraction, expression of nuclear genes, and mitochondrial fraction, along with enrichments of cardiomyocyte and non-cardiomyocyte genes. These metrics are used to cluster the nuclei, within a contamination/quality space embedding, using unsupervised clustering. The resulting clusters allow for the identification and removal of empty and highly contaminated nuclei. In addition, further filtering based on samples-specific distribution of quality metrics (splicing fraction and mitochondrial fraction) is applied. Detailed information can be found in,[25] with its source code available on GitHub (https://github.com/scHEARTGROUP/qclus). The QClus pipeline uses Scrublet[92] to filter doublets. In some samples, the elbow-curve shape was too subtle for Cell Ranger to accurately partition empty and non-empty droplets. This issue was solved with QClus which accurately removed empty droplets with high ambient RNA content, resulting in median of 5318 and mean of 5817 droplets per sample. Barcode rank plot distribution for each sample is shown in Figure S11.

After QClus filtering, all samples raw counts were merged, normalized, and then transformed in log values (ln (counts per 10000) + 1). The resulting normalized matrix was used for visualization of gene expression. Gene and transcript count in heart tissue and pericardial fluid are strongly associated (Figure S12A). QC metrics distribution for heart samples are shown in Figure S12B.

Expression correction, sample heterogeneity correction, embedding and clustering

Genes that were expressed in less than 10 cells (across all samples) were filtered out. Variability filtering of genes was also applied (minimum mean of 0.0125, maximum mean of 3 and minimum normalized dispersion of 0.5). Linear regression was used to correct for the number of counts per cell and mitochondrial percentage. Subsequent steps were standard scaling, Principal Component Analysis (PCA) and Harmony batch correction[93] at the sample level. Afterward, a 10 nearest neighbor graph was constructed, based on the top 40 PCs, which was used for UMAP embedding.[94,95] Clusters were found using the Leiden algorithm[96] at resolution 1.

Cell type identification

Clusters were annotated through cell-type specific marker expression and correlation. Annotations were additionally confirmed through gene set enrichment using Enrichr[97] (from gseapy[98] wrapper), from the first 500 significantly differentially expressed genes (Wilcoxon ranked sum, Benjamini-Hochberg[99] FDR <0.05). At the cell-typing step and further subtyping, when a Leiden cluster[96] showed high level expression of all markers from two distinct cell-types, it was removed as a doublet cluster. Cell-type specific scores were calculated by using the *score_genes* of scanpy[91] with marker genes from these cell-types.

Cell subtyping

Seven main sets of cells were established by combining 12 discovered cell-types, namely cardiomyocytes, endocardial endothelial cells, fibroblasts, mesothelial cells, vascular cells (containing pericytes, smooth muscle cells, and vascular endothelial cells) immune cells (containing lymphocytes and macrophages), and neuro-adipo cells (containing Schwann cells, adipocytes, and neurons). Subclusters were found through the reprocessing of each set, by regression, scaling and PCA steps. Batch correction was also performed again at the sample level, in addition with k nearest neighbors' (knn) graph and UMAP plot. Clusters were discovered with Leiden clustering (resolution 1).[96] For each set, small clusters were found to be enriched for markers specific for other cell types and were thus removed as doublets.

Differential expression analysis

For the main cell types (i.e., cardiomyocytes, fibroblasts, endocardial endothelial cells, and mesothelial cells), we analyzed differential gene expression for the different groups (IHD, IHF, and NIHF) in 'disease-enriched clusters' (Leiden clusters that were most densely populated by a disease group) compared to control cells from 'control clusters' (Leiden clusters that were most densely populated by control cells). These 'disease-enriched clusters' and 'control clusters' had a clear shift between their locations in the UMAP embedding. For vascular and immune cells of the heart tissue, and the pericardial fluid cells, differential expression was determined within a subtype population (i.e., coronary artery endothelial cells in a disease group against coronary artery endothelial cells in a control group).

Apart from cluster-specific marker identification, differential gene expression analysis was performed using the NEBULA[30] R package. Mitochondrial percentage, patient age and patient sex (based on chromosomal genotype). were used as additive confounding covariates in the model. As advised by the documentation, the total number of counts for each cell was imputed as the scaling factor. The LN method was used when the number of cells exceeded 1000, and the HL method when not. Benjamini-Hochberg method[99] was used for FDR correction.

RNA-seq on HUVECs for disturbed flow experiment

Fastq files were processed and reads aligned using nf-core RNA-seq pipeline.[100] Briefly, after QC filtering and trimming, the pipeline used STAR[101] to align the reads to the hg38 Genome, and Salmon[102] to create a count matrix. The resulting length scaled count matrix was used as an input to DESeq2[103] for differential expression analysis.

RNA-seq on HAECs for IL-1β time course analysis

The sequencing reads obtained were trimmed to 3′ A-stretches originating from the library preparation and poor-quality reads were filtered out (minimum 97% of bp over quality cutoff 10). Remaining reads were aligned to GRCh37hg19 reference genome using STAR v2.5.4b[101] with ENCODE recommended options for long RNA-seq analysis.[119] Reads located on exonic locations were quantified using HOMER v4.9[104] 'analyzeRepeats' routine with the settings '-condense genes -count exons'. Differential expression was calculated using limma version 3.46.0[105] after filtering of low expressed transcripts (CPM >0.5 in at least 2 samples) to improve sensitivity and precision of the analysis.[120]

Ingenuity Pathway Analysis

The affected 'Diseases and biological functions', 'Canonical pathways' and 'Upstream regulators' were studied using the 'Core analysis' and 'Comparison analysis' of the Ingenuity Pathway Analysis (IPA, QIAGEN Inc., https://digitalinsights.qiagen.com/IPA)[31] based on the differentially expressed genes for each cell type/cell subtype. The data tables can be found from the website

(http://compbio2.mit.edu/scheart/). Pathway overlaps were explored using Venny (https://bioinfogp.cnb.csic.es/tools/venny/index.html).

Resolve molecular cartography

The data obtained from Resolve Bioscience Molecular Cartography spatial transcriptomics were analyzed using a tailored method in Python to overlap the spatial expression of genes of interest from specific ROIs. We created a grid of 80px wide hexagons, covering the whole slide, and counted signals within each, simulating small, high-resolution spots. We used the Scanpy[91] and Squidpy[106] methods to process the resulting data. The samples were merged into one matrix. As spots contained few counts, and only 100 genes were characterized, the data were not normalized to prevent artifacts. The counts were logarithmized (using log1p function of Scanpy) and linear regression was performed to eliminate the effect of global expression levels between spots (number of counts and number of genes) and the data were scaled (standard scaling, max value of 10). Afterward, PCA was performed, as well as sample-level batch correction, using Harmony.[93] Then, a knn network was constructed for the creation of a UMAP embedding,[95] using the top 15 PCs. Clusters were discovered using the Leiden algorithm.[96] For detailed plotting, each count was plotted as a dot of a particular color, corresponding to a gene, at the exact location where it was associated in the original data, resulting from the experiment. Transcript number distributions and counts/genes per spot are shown in Figure S13A and average per sample in Figure S13B.

Visium spatial transcriptomics

Samples were processed using Scanpy 1.9.1.[91] All sample data matrices were merged into one matrix which was then processed. Counts were normalized (total count of 10,000 per capture area) and logarithmized (using log1p function of Scanpy). For dimension reduction purposes, the raw normalized matrix was further processed: genes that were not characterized as highly variable enough were filtered out (minimum mean of 0.0125, maximum mean of 3 and minimum normalized dispersion of 0.5), and linear regression was performed to eliminate the effect of covariates (total_counts, and mitochondrial genes percentage). The data were then scaled (standard scaling, max value of 10). Afterward, PCA was performed, as well as sample-level batch correction, using Harmony.[93] Then, a knn network was constructed for the creation of a UMAP embedding.[95] Clusters were discovered using the Leiden algorithm.[96] Cell-type-specific scores were calculated by using the *score_genes* of scanpy with marker genes of these cell-types, originating from the snRNA-seq data. The quality metrics for Visium are shown in Figures S14 and S15.

Gene expression modules

We calculated gene expression modules for each major cell class using the scmodule package (manuscript in preparation). Briefly, the method first adjusts the gene expression matrix $\mathbf{X}$ by performing zero-phase component analysis (ZCA) to remove cell-cell correlation structure, which biases the recovery of gene-gene modules. We calculate the SVD of $\mathbf{X}$ as $\mathbf{USV^T}$ and use it to approximate the gene-gene correlation matrix of the ZCA adjusted expression matrix as $C_{ZCA} \sim \mathbf{VS}^{P}\mathbf{V^T}$ at multiple resolutions $\mathbf{P}$ in [0,0.5,1]. For each matrix, we Z score the estimated correlations controlling for sparsity of each gene (number of zeros in the expression matrix) and build a gene-gene graph with correlations with $z > 4.5$. Finally, we detect modules of genes by multi-graph Leiden clustering[96] on the ensemble of graphs (with leiden resolution = 2).

GWAS linked genes

Published gene prioritization results were collected across multiple different prioritization approaches and GWAS studies. The OpenTargets Genetics Portal[88] (data release: June 2021) was used to obtain data for the GWAS studies of CAD, myocardial infarction, and stroke[69,121–126] (FinnGen[127] and UK Biobank[128] data via the OpenTargets study accessions FINNGEN_R5_I9_CORATHER, SAIGE_411, SAIGE_411_2 and SAIGE_411_4). For OpenTargets, the Locus2Gene algorithm prioritized 238 genes, eQTL colocalization 164 genes, and 'nearest gene' 387 genes.

From the recent CAD GWAS by Aragam *et al.,*[129] the per-association overall top-prioritized genes contributed 186 genes, PoPS prioritization 386 genes, 'nearest gene' 216 genes, and, for the GWAS 1% FDR threshold associations, 'nearest gene' contributed 716 genes.

The TWAS of CAD by Li *et al.*[89] prioritized 114 genes and the CAD GWAS review by Erdmann et al.[90] listed 373 genes at CAD loci.

Prediction of TF binding motif disruption

To predict disruption of TF binding due to an SNP, the candidate SNPs were analyzed using all 94 high-confidence binding models that were published by Yan *et al.*[70] and derived by training deltaSVM models on *in vitro* protein–DNA binding data. The model is based on systematic measurements of the binding of 270 human transcription factors to 95,886 noncoding variants in the human genome using an ultra-high-throughput multiplex protein–DNA binding assay, SNP-SELEX. The resulting 828 million measurements of transcription factor–DNA interactions enabled the estimation of the relative affinity of these transcription factors to each variant *in vitro*. The original authors' recommended thresholds were used for determining sequence binding and allelic disruption. In addition, the consequence of the motif disruption was predicted based on the work of Kheradpour and Kellis[71] that is based on systematic motif analysis of 427 human ChIP-seq datasets.

Cell Reports Medicine, Volume *5*

Supplemental information

Transcriptomic and spatial dissection of human *ex vivo* right atrial tissue reveals proinflammatory microvascular changes in ischemic heart disease

Suvi Linna-Kuosmanen, Eloi Schmauch, Kyriakitsa Galani, Johannes Ojanen, Carles A. Boix, Tiit Örd, Anu Toropainen, Prosanta K. Singha, Pierre R. Moreau, Kristiina Harju, Adriana Blazeski, Åsa Segerstolpe, Veikko Lahtinen, Lei Hou, Kai Kang, Elamaran Meibalan, Leandro Z. Agudelo, Hannu Kokki, Jari Halonen, Juho Jalkanen, Jarmo Gunn, Calum A. MacRae, Maija Hollmén, Juha E.K. Hartikainen, Minna U. Kaikkonen, Guillermo García-Cardeña, Pasi Tavi, Tuomas Kiviniemi, and Manolis Kellis

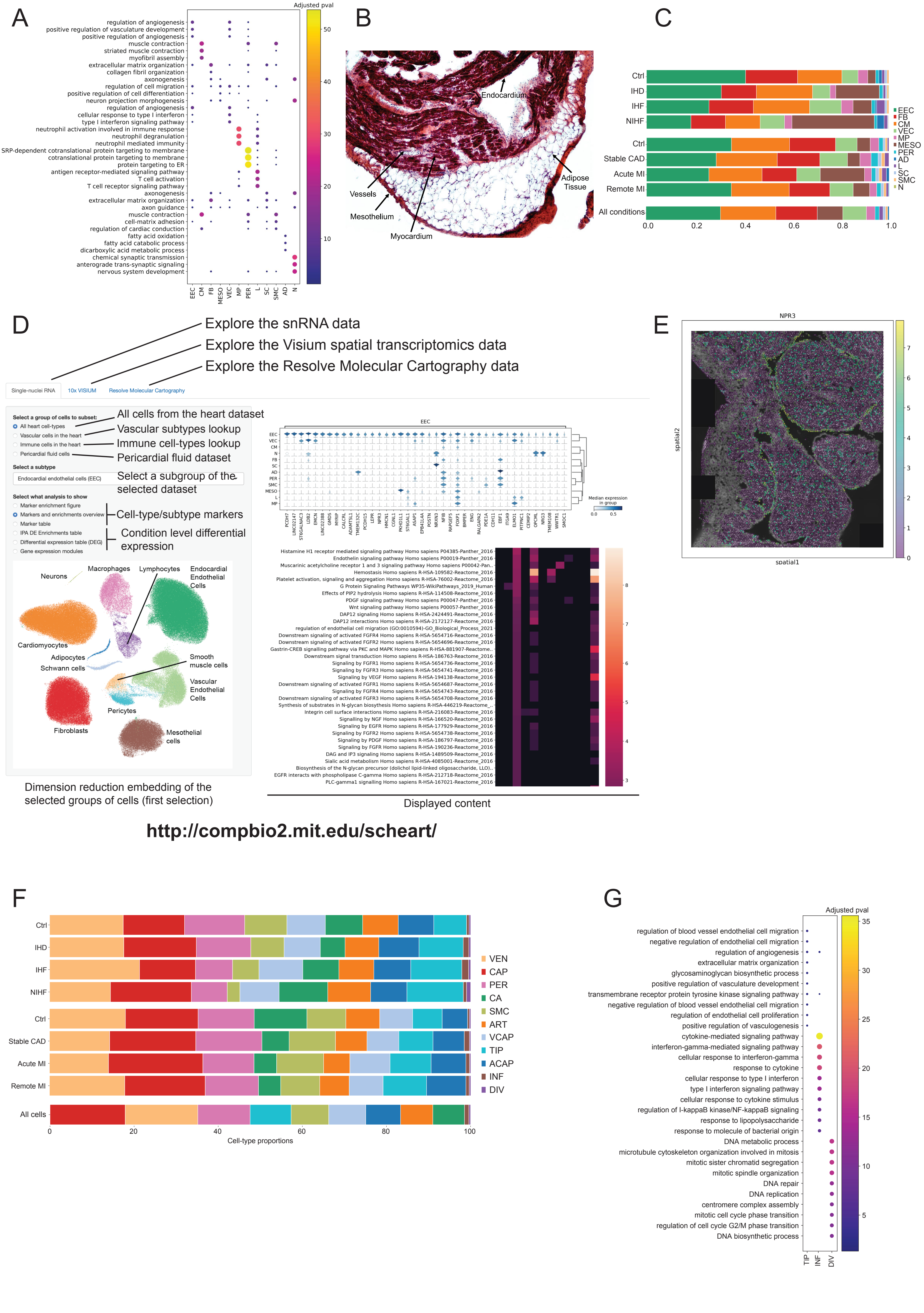
A
Adjusted pval
regulation of angiogenesis
positive regulation of vasculature development
positive regulation of angiogenesis
muscle contraction
striated muscle contraction
myofibril assembly
extracellular matrix organization
collagen fibril organization
axonogenesis
regulation of cell migration
positive regulation of cell differentiation
neuron projection morphogenesis
regulation of angiogenesis
cellular response to type I interferon
type I interferon signaling pathway
neutrophil activation involved in immune response
neutrophil degranulation
neutrophil mediated immunity
SRP-dependent cotranslational protein targeting to membrane
cotranslational protein targeting to membrane
protein targeting to ER
antigen receptor-mediated signaling pathway
T cell activation
T cell receptor signaling pathway
axonogenesis
extracellular matrix organization
axon guidance
muscle contraction
cell-matrix adhesion
regulation of cardiac conduction
fatty acid oxidation
fatty acid catabolic process
dicarboxylic acid metabolic process
chemical synaptic transmission
anterograde trans-synaptic signaling
nervous system development
EEC CM FB MESO VEC MP PER L SC SMC AD N
10 20 30 40 50
B
Endocardium
Vessels
Mesothelium
Myocardium
Adipose Tissue
C
Ctrl
IHD
IHF
NIHF
Ctrl
Stable CAD
Acute MI
Remote MI
All conditions
0.0 0.2 0.4 0.6 0.8 1.0
EEC FB CM VEC MP MESO PER AD L SC SMC N
D
Explore the snRNA data
Explore the Visium spatial transcriptomics data
Explore the Resolve Molecular Cartography data
Single-nuclei RNA
10x VISIUM
Resolve Molecular Cartography
Select a group of cells to subset:
All heart cell-types
Vascular cells in the heart
Immune cells in the heart
Pericardial fluid cells
All cells from the heart dataset
Vascular subtypes lookup
Immune cell-types lookup
Pericardial fluid dataset
Select a subtype
Endocardial endothelial cells (EEC)
Select a subgroup of the selected dataset
Select what analysis to show
Marker enrichment figure
Markers and enrichments overview
Marker table
IPA DE Enrichments table
Differential expression table (DEG)
Gene expression modules
Cell-type/subtype markers
Condition level differential expression
Neurons
Macrophages
Lymphocytes
Endocardial Endothelial Cells
Cardiomyocytes
Adipocytes
Schwann cells
Smooth muscle cells
Vascular Endothelial Cells
Pericytes
Fibroblasts
Mesothelial cells
Dimension reduction embedding of the selected groups of cells (first selection)
EEC
EEC VEC CM N FB SC AD PER SMC MESO L MP
Median expression in group
0.0 0.5
Histamine H1 receptor mediated signaling pathway Homo sapiens P04385-Panther_2016
Endothelin signaling pathway Homo sapiens P00019-Panther_2016
Muscarinic acetylcholine receptor 1 and 3 signaling pathway Homo sapiens P00042-Pan..
Hemostasis Homo sapiens R-HSA-109582-Reactome_2016
Platelet activation, signaling and aggregation Homo sapiens R-HSA-76002-Reactome_2016
G Protein Signaling Pathways WP35-WikiPathways_2019_Human
Effects of PIP2 hydrolysis Homo sapiens R-HSA-114508-Reactome_2016
PDGF signaling pathway Homo sapiens P00047-Panther_2016
Wnt signaling pathway Homo sapiens P00057-Panther_2016
DAP12 signaling Homo sapiens R-HSA-2424491-Reactome_2016
DAP12 interactions Homo sapiens R-HSA-2172127-Reactome_2016
regulation of endothelial cell migration (GO:0010594)-GO_Biological_Process_2021
Downstream signaling of activated FGFR4 Homo sapiens R-HSA-5654716-Reactome_2016
Downstream signaling of activated FGFR2 Homo sapiens R-HSA-5654696-Reactome_2016
Gastrin-CREB signalling pathway via PKC and MAPK Homo sapiens R-HSA-881907-Reactome..
Downstream signal transduction Homo sapiens R-HSA-186763-Reactome_2016
Signaling by FGFR1 Homo sapiens R-HSA-5654736-Reactome_2016
Signaling by FGFR3 Homo sapiens R-HSA-5654741-Reactome_2016
Signaling by VEGF Homo sapiens R-HSA-194138-Reactome_2016
Downstream signaling of activated FGFR1 Homo sapiens R-HSA-5654687-Reactome_2016
Signaling by FGFR4 Homo sapiens R-HSA-5654743-Reactome_2016
Downstream signaling of activated FGFR3 Homo sapiens R-HSA-5654708-Reactome_2016
Synthesis of substrates in N-glycan biosythesis Homo sapiens R-HSA-446219-Reactome_..
Integrin cell surface interactions Homo sapiens R-HSA-216083-Reactome_2016
Signalling by NGF Homo sapiens R-HSA-166520-Reactome_2016
Signaling by EGFR Homo sapiens R-HSA-177929-Reactome_2016
Signaling by FGFR2 Homo sapiens R-HSA-5654738-Reactome_2016
Signaling by PDGF Homo sapiens R-HSA-186797-Reactome_2016
Signaling by FGFR Homo sapiens R-HSA-190236-Reactome_2016
DAG and IP3 signaling Homo sapiens R-HSA-1489509-Reactome_2016
Sialic acid metabolism Homo sapiens R-HSA-4085001-Reactome_2016
Biosynthesis of the N-glycan precursor (dolichol lipid-linked oligosaccharide, LLO)..
EGFR interacts with phospholipase C-gamma Homo sapiens R-HSA-212718-Reactome_2016
PLC-gamma1 signalling Homo sapiens R-HSA-167021-Reactome_2016
3 4 5 6 7 8
Displayed content
http://compbio2.mit.edu/scheart/
E
NPR3
spatial2
spatial1
0 1 2 3 4 5 6 7
F
Ctrl
IHD
IHF
NIHF
Ctrl
Stable CAD
Acute MI
Remote MI
All cells
0 20 40 60 80 100
Cell-type proportions
VEN CAP PER CA SMC ART VCAP TIP ACAP INF DIV
G
Adjusted pval
regulation of blood vessel endothelial cell migration
negative regulation of endothelial cell migration
regulation of angiogenesis
extracellular matrix organization
glycosaminoglycan biosynthetic process
positive regulation of vasculature development
transmembrane receptor protein tyrosine kinase signaling pathway
negative regulation of blood vessel endothelial cell migration
regulation of endothelial cell proliferation
positive regulation of vasculogenesis
cytokine-mediated signaling pathway
interferon-gamma-mediated signaling pathway
cellular response to interferon-gamma
response to cytokine
cellular response to type I interferon
type I interferon signaling pathway
cellular response to cytokine stimulus
regulation of I-kappaB kinase/NF-kappaB signaling
response to lipopolysaccharide
response to molecule of bacterial origin
DNA metabolic process
microtubule cytoskeleton organization involved in mitosis
mitotic sister chromatid segregation
mitotic spindle organization
DNA repair
DNA replication
centromere complex assembly
mitotic cell cycle phase transition
regulation of cell cycle G2/M phase transition
DNA biosynthetic process
TIP INF DIV
5 10 15 20 25 30 35

Fig. S1. Heart cell-types and subtypes distribution and enrichments. Related to Figures 1 and 2.

A. Top 3 enriched gene sets (GO Biological Process) for top 1000 genes with FDR < 0.05 for each cell type. Dot color and size is -log10(adjusted p-value). Only significant (adjusted p-value < 0.05) enrichments are shown. **B.** Hematoxylin-eosin-stained histology slide of a right atrial appendage sample from a patient with IHD (included in the dataset). **C.** Proportion of the main cell types, per condition. **D.** Interactive website overview. An interactive website is accessible at http://compbio.mit.edu/scheart. It provides access to the analyzed snRNA-seq and spatial transcriptomics data (both Visium and Resolve Molecular Cartography) presented in this study. The user can select the ensemble of cells to focus on (all heart cells, vascular heart cells, immune heart cells, pericardial fluid data) and explore different information types (1. Gene set enrichment of subtype/cell-type marker genes 2. Subtype/cell-type marker gene overview. 3. Subtype/cell-type marker genes table. 4. IPA enrichment table from differential expression result of a selected condition in the subtype/cell-type. 5. Differential expression result table of a selected condition in the subtype/cell-type. 6. Gene expression module analysis in the selected subtype/cell-type). **E.** Spatial expression of *NPR3* on a Resolve molecular cartography section of the right atrium in IHD sample. **F.** Proportion of vascular cells, per condition. **G**. Top 10 enriched gene sets (GO Biological Process) for top 200 genes with FDR < 0.05 in each subtype, for tip cells (TIP), inflammatory endothelial cells (INF) and dividing endothelial cells (DIV). Dot color and size is -log10(adjusted enrichment p-value). Only significant (adjusted p-value < 0.05) enrichments are shown.

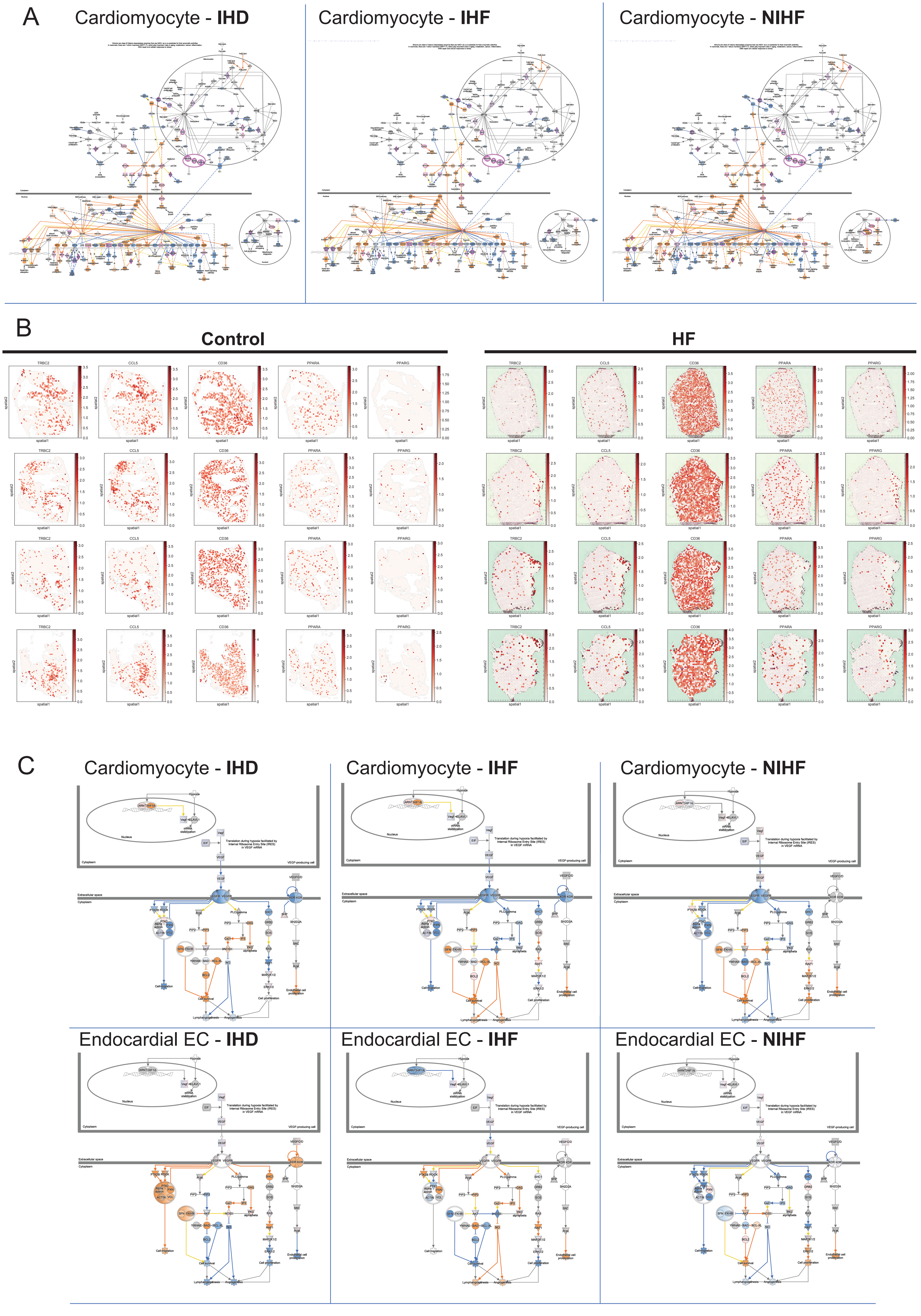
A
Cardiomyocyte - IHD
Cardiomyocyte - IHF
Cardiomyocyte - NIHF
B
Control
HF
TRBC2
CCL5
CD36
PPARA
PPARG
spatial1
spatial2
C
Cardiomyocyte - IHD
Cardiomyocyte - IHF
Cardiomyocyte - NIHF
Endocardial EC - IHD
Endocardial EC - IHF
Endocardial EC - NIHF

Fig. S2. Signaling pathway activity enrichment across condition and spatial transcriptomics gene expression changes. Related to Figure 3.

A. Sirtuin signaling pathway from IPA in cardiomyocytes in IHD, IHF, and NIHF. Colors indicate measured and inferred expression/activity changes. Blue stands for downregulation or inhibition, orange/red for upregulation. Yellow line indicates inconsistent changes and purple highlights measured significant changes in the dataset. **B.** Expression of *TRBC2*, *CCL5*, *CD36*, *PPARA*, *PPARG* on Visium slides, from a patient with heart failure and a control sample (n=4). **C.** VEGF signaling pathway from IPA in cardiomyocytes and endocardial endothelial cells in IHD, IHF, and NIHF. Blue stands for downregulation or inhibition of the molecule, orange/red for upregulation. Yellow line indicates inconsistent changes.

Coronary artery EC - **IHD**

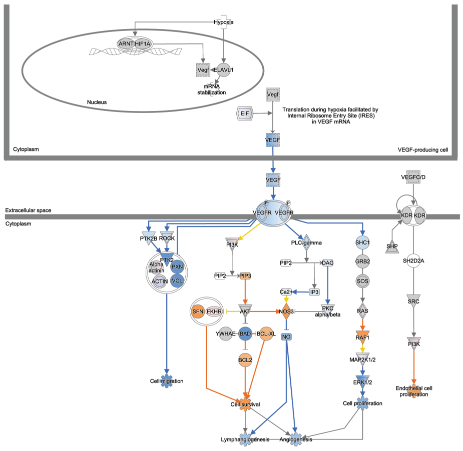

Coronary artery EC - **IHF**

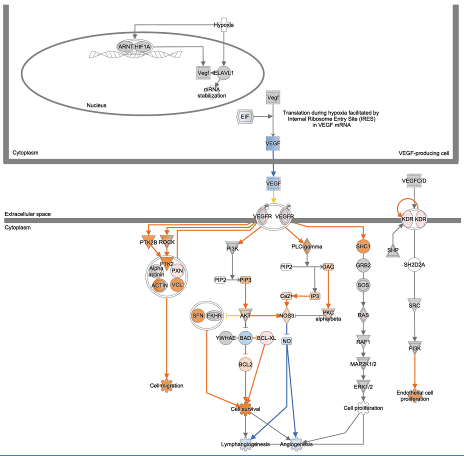

Coronary artery EC - **NIHF**

No enrichement

Arterial capillary EC - **IHD**

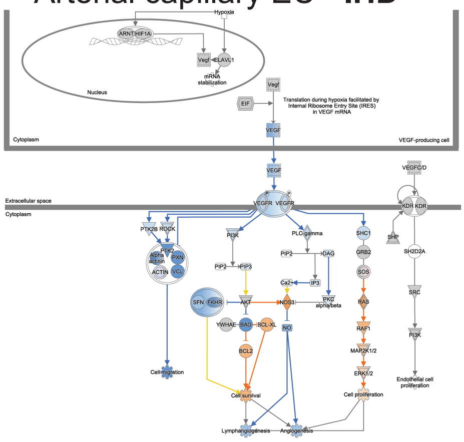

Arterial capillary EC - **IHF**

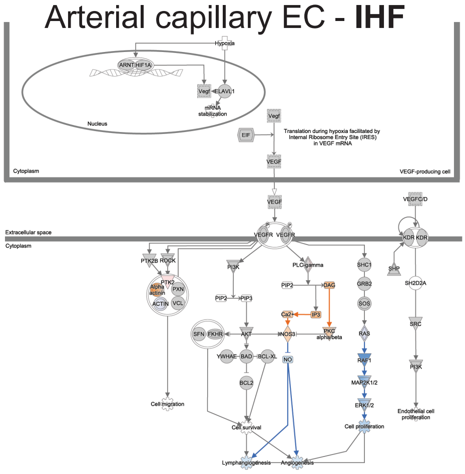

Arterial capillary EC - **NIHF**

No enrichement

Capillary EC - **IHD**

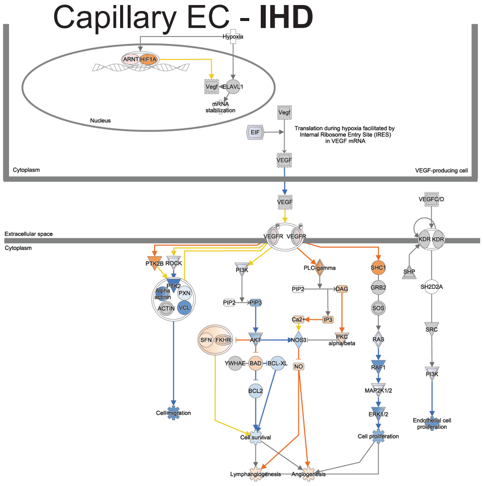

Capillary EC - **IHF**

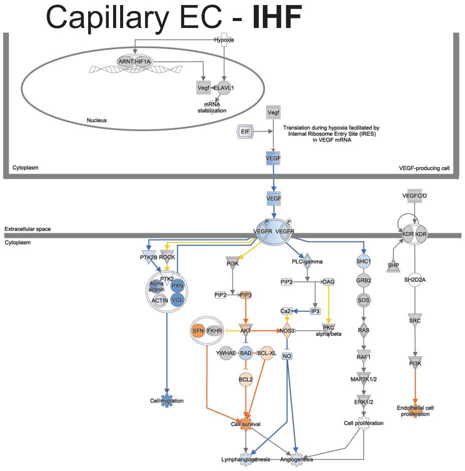

Capillary EC - **NIHF**

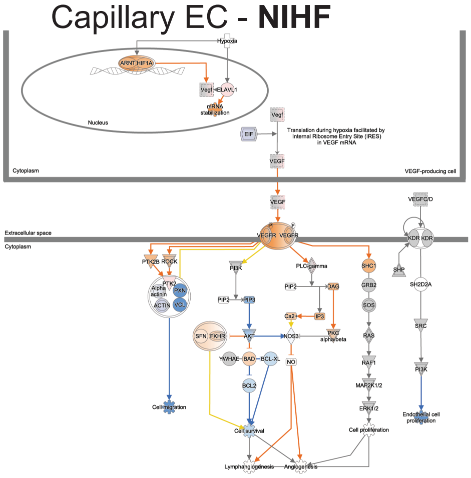

Tip cells (EC) - **IHD**

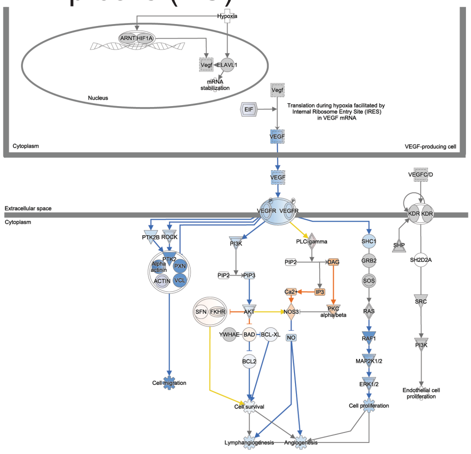

Tip cells (EC) - **IHF**

No enrichement

Tip cells (EC) - **NIHF**

No enrichement

Fig. S3. VEGF signaling pathway from IPA in endothelial subtypes. Related to Figure 3.

Blue stands for downregulation or inhibition, orange/red for upregulation. Yellow line indicates inconsistent changes.

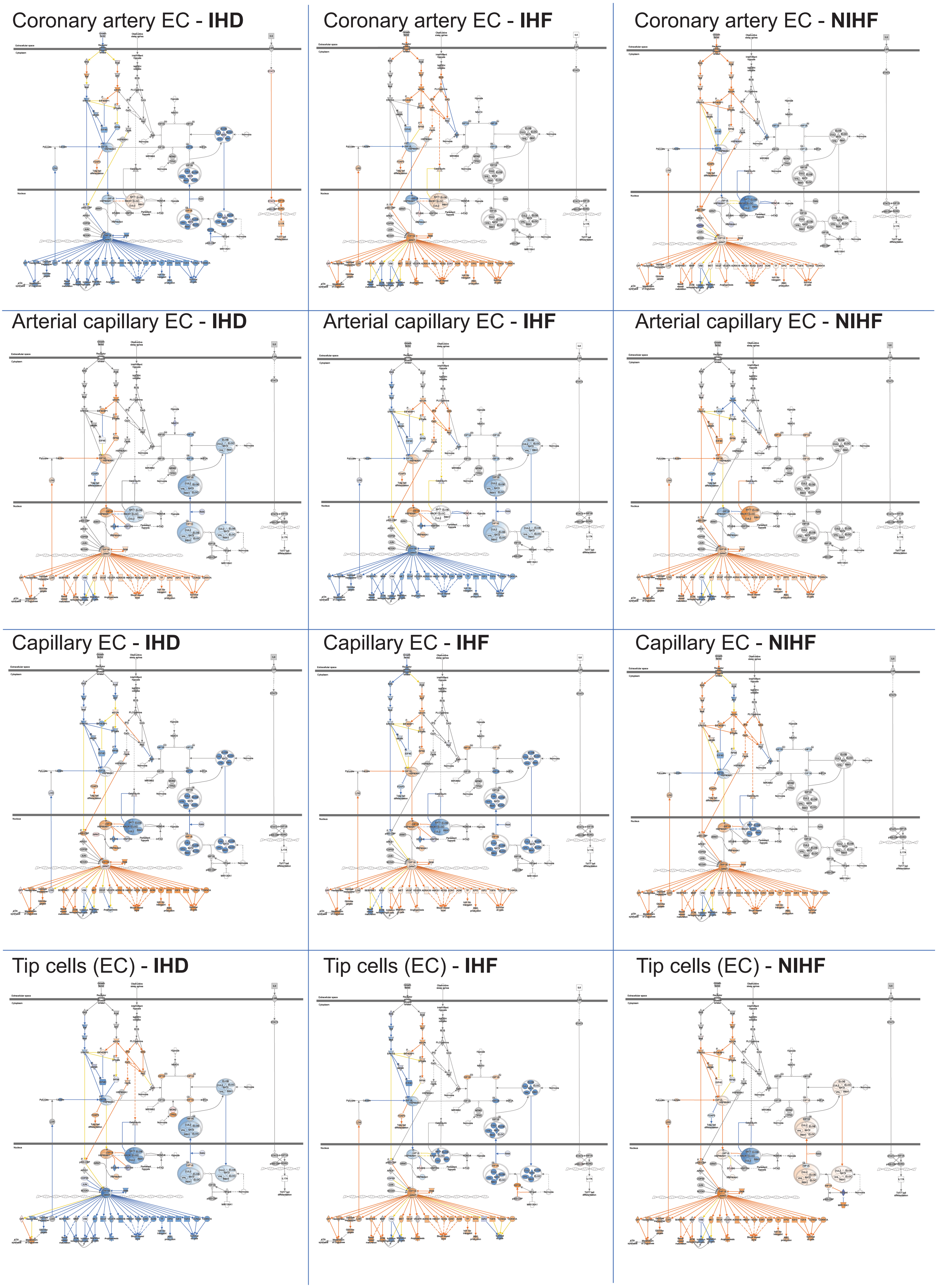
Coronary artery EC - IHD
Coronary artery EC - IHF
Coronary artery EC - NIHF
Arterial capillary EC - IHD
Arterial capillary EC - IHF
Arterial capillary EC - NIHF
Capillary EC - IHD
Capillary EC - IHF
Capillary EC - NIHF
Tip cells (EC) - IHD
Tip cells (EC) - IHF
Tip cells (EC) - NIHF

Fig. S4. HIF signaling pathway from IPA in endothelial subtypes in IHD, IHF, and NIHF. Related to Figure 3.

Blue stands for downregulation or inhibition, orange/red for upregulation. Yellow line indicates inconsistent changes.

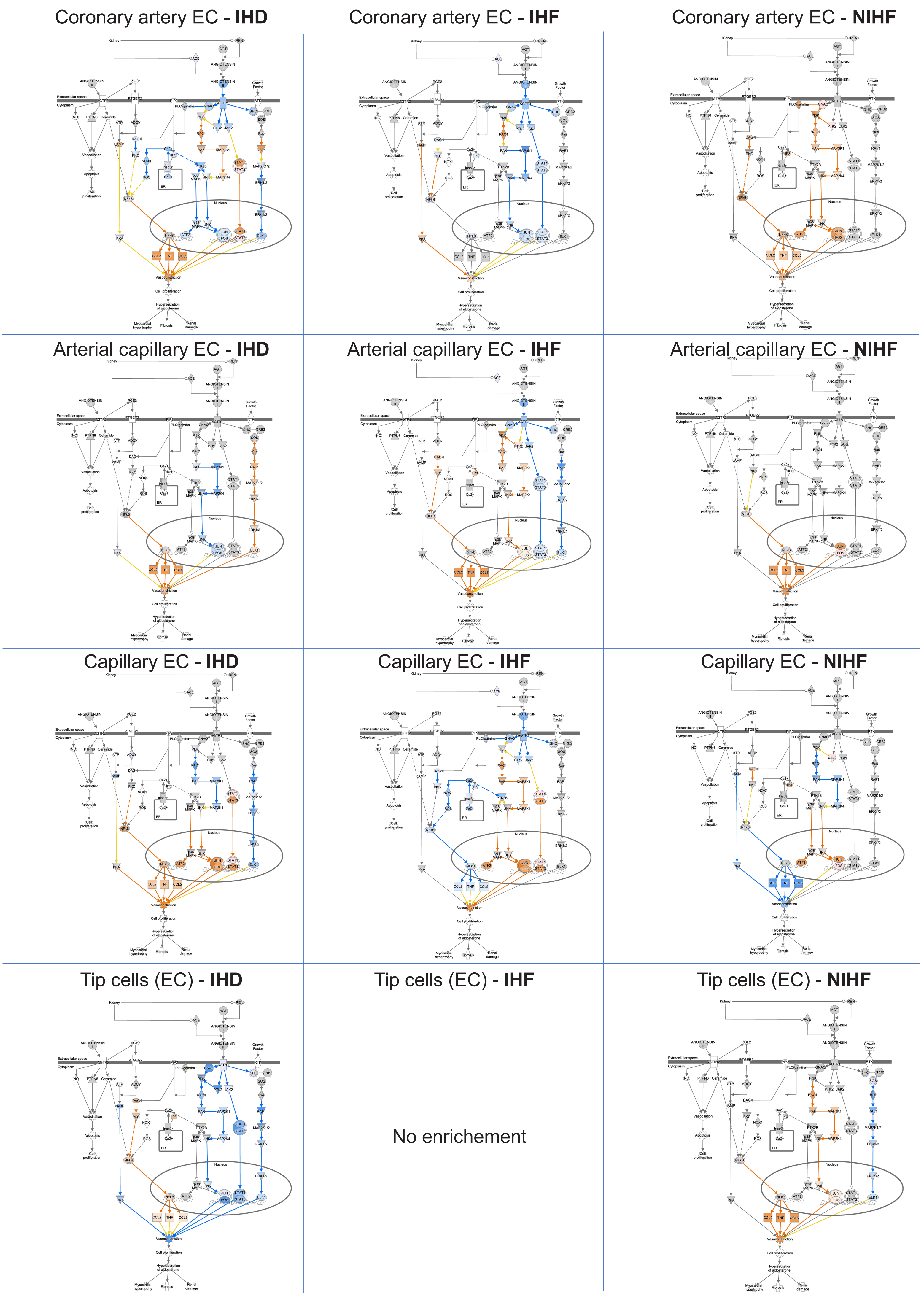
Coronary artery EC - **IHD**
Coronary artery EC - **IHF**
Coronary artery EC - **NIHF**
Arterial capillary EC - **IHD**
Arterial capillary EC - **IHF**
Arterial capillary EC - **NIHF**
Capillary EC - **IHD**
Capillary EC - **IHF**
Capillary EC - **NIHF**
Tip cells (EC) - **IHD**
Tip cells (EC) - **IHF**
No enrichement
Tip cells (EC) - **NIHF**

Fig. S5. Renin signaling pathway from IPA in endothelial subtypes in IHD, IHF, and NIHF. Related to Figure 3.

Blue stands for downregulation or inhibition, orange/red for upregulation. Yellow line indicates inconsistent changes.

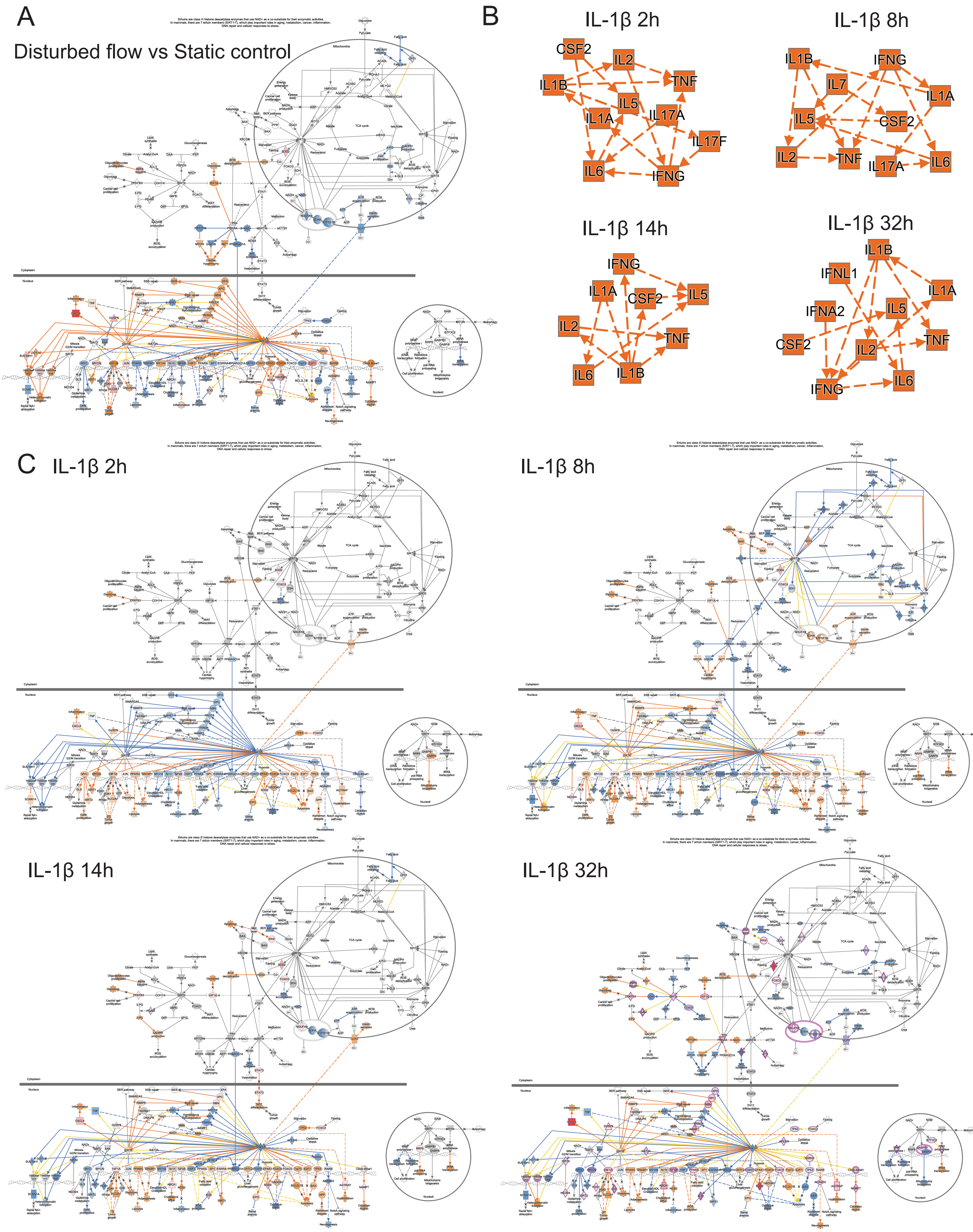
A
Disturbed flow vs Static control
B
IL-1β 2h
CSF2
IL2
TNF
IL1B
IL5
IL1A
IL17A
IL17F
IL6
IFNG
IL-1β 8h
IL1B
IFNG
IL7
IL1A
IL5
CSF2
IL2
TNF
IL17A
IL6
IL-1β 14h
IFNG
IL1A
CSF2
IL5
IL2
TNF
IL6
IL1B
IL-1β 32h
IL1B
IFNL1
IL1A
IFNA2
IL5
CSF2
TNF
IL2
IFNG
IL6
C
IL-1β 2h
IL-1β 8h
IL-1β 14h
IL-1β 32h

Fig. S6. Endothelial cell response to disturbed flow and IL-1b stimulation. Related to Figure 3.

A. Sirtuin signaling pathway from IPA in HUVEC cells under atheroprone (disturbed) flow versus static cells (*N*=3). Blue stands for downregulation or inhibition, orange/red for upregulation. Yellow line indicates inconsistent changes. **B.** IL-1b network from IPA in stimulated HAEC cells. The cells were treated for 2, 8, 14, or 32h with IL-1b. The resulting RNA-seq data were analyzed with IPA (Qiagen) to construct the upstream regulator networks from the graphical summaries. **C.** Sirtuin signaling pathway from IPA in IL-1b-treated HAECs after 2, 8, 14, and 32h of treatment. Blue stands for downregulation or inhibition, orange/red for upregulation. Yellow line indicates inconsistent changes and purple highlights measured significant changes in the dataset. All signaling pathways were constructed with IPA (Qiagen) from either core or comparison analysis.

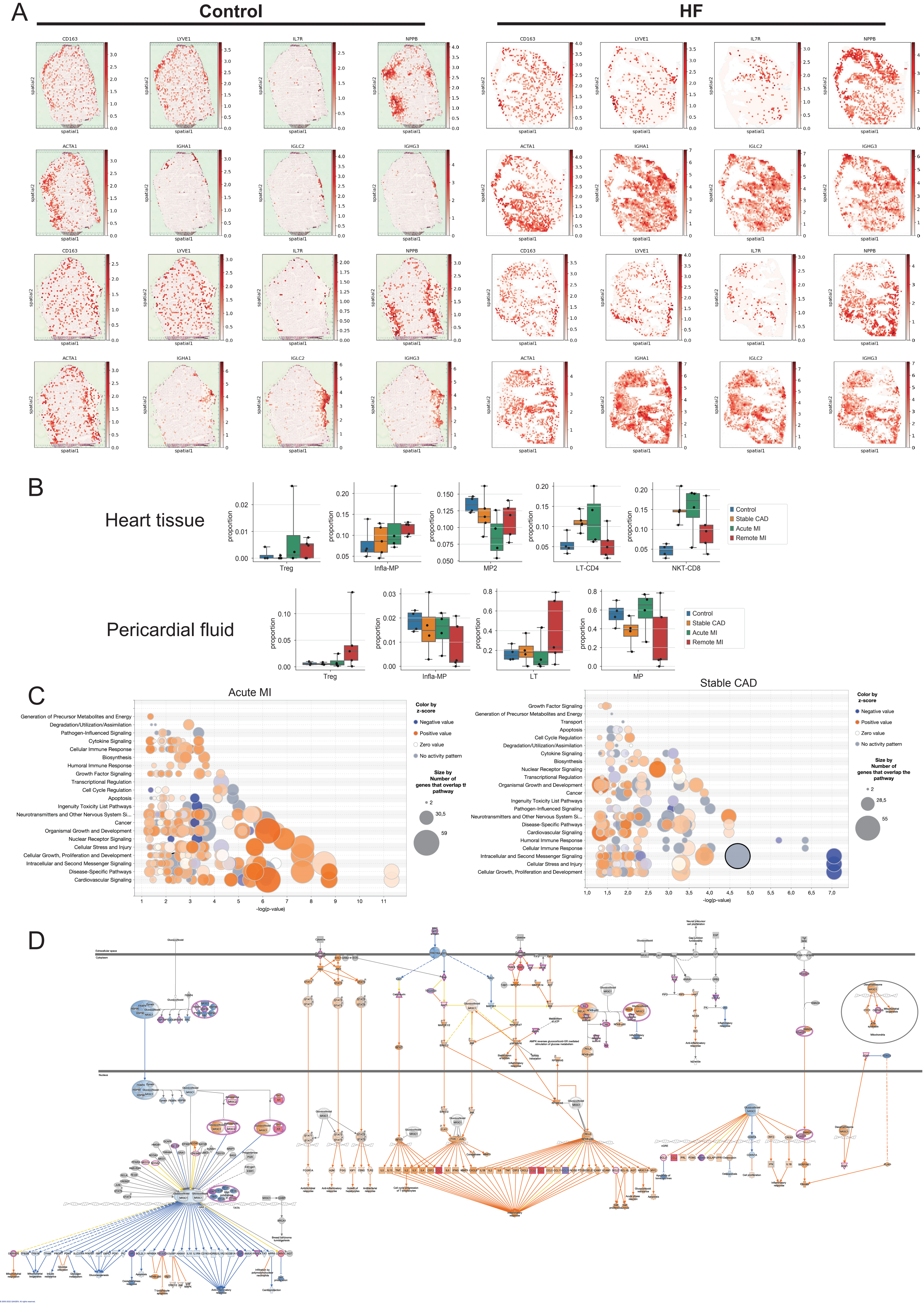

A
Control
HF
CD163
LYVE1
IL7R
NPPB
ACTA1
IGHA1
IGLC2
IGHG3
spatial1
spatial2
B
Heart tissue
Pericardial fluid
proportion
Treg
Infla-MP
MP2
LT-CD4
NKT-CD8
LT
MP
Control
Stable CAD
Acute MI
Remote MI
C
Acute MI
Stable CAD
Generation of Precursor Metabolites and Energy
Degradation/Utilization/Assimilation
Pathogen-Influenced Signaling
Cytokine Signaling
Cellular Immune Response
Biosynthesis
Humoral Immune Response
Growth Factor Signaling
Transcriptional Regulation
Cell Cycle Regulation
Apoptosis
Ingenuity Toxicity List Pathways
Neurotransmitters and Other Nervous System Si...
Cancer
Organismal Growth and Development
Nuclear Receptor Signaling
Cellular Stress and Injury
Cellular Growth, Proliferation and Development
Intracellular and Second Messenger Signaling
Disease-Specific Pathways
Cardiovascular Signaling
Transport
-log(p-value)
Color by z-score
Negative value
Positive value
Zero value
No activity pattern
Size by Number of genes that overlap th pathway
Size by Number of genes that overlap the pathway
D

Fig. S7. Immune cell proportion changes and transcriptional response. Related to Figures 4 and 5.

A. Expression of *CD163*, *LYVE1*, *IL7R* on Visium slides, from a patient with heart failure and a control sample (*n*=4). **B.** Proportion of selected immune cells across conditions in heart tissue and pericardial fluid. Whiskers show the maximum and minimum values, except for outliers (more than 1.5 times the interquartile). **C.** Enrichment of general terms and pathway sharing between terms in macrophages (MP) in stable CAD and acute MI from IPA. A pathway shared between the terms is shown as a circle of the same size and color on the same vertical line between the terms. **D.** Glucocorticoid signaling pathway from IPA in MPs in stable CAD compared to control. Blue stands for downregulation or inhibition, orange/red for upregulation. Yellow line indicates inconsistent changes and purple highlights measured significant changes in the dataset.

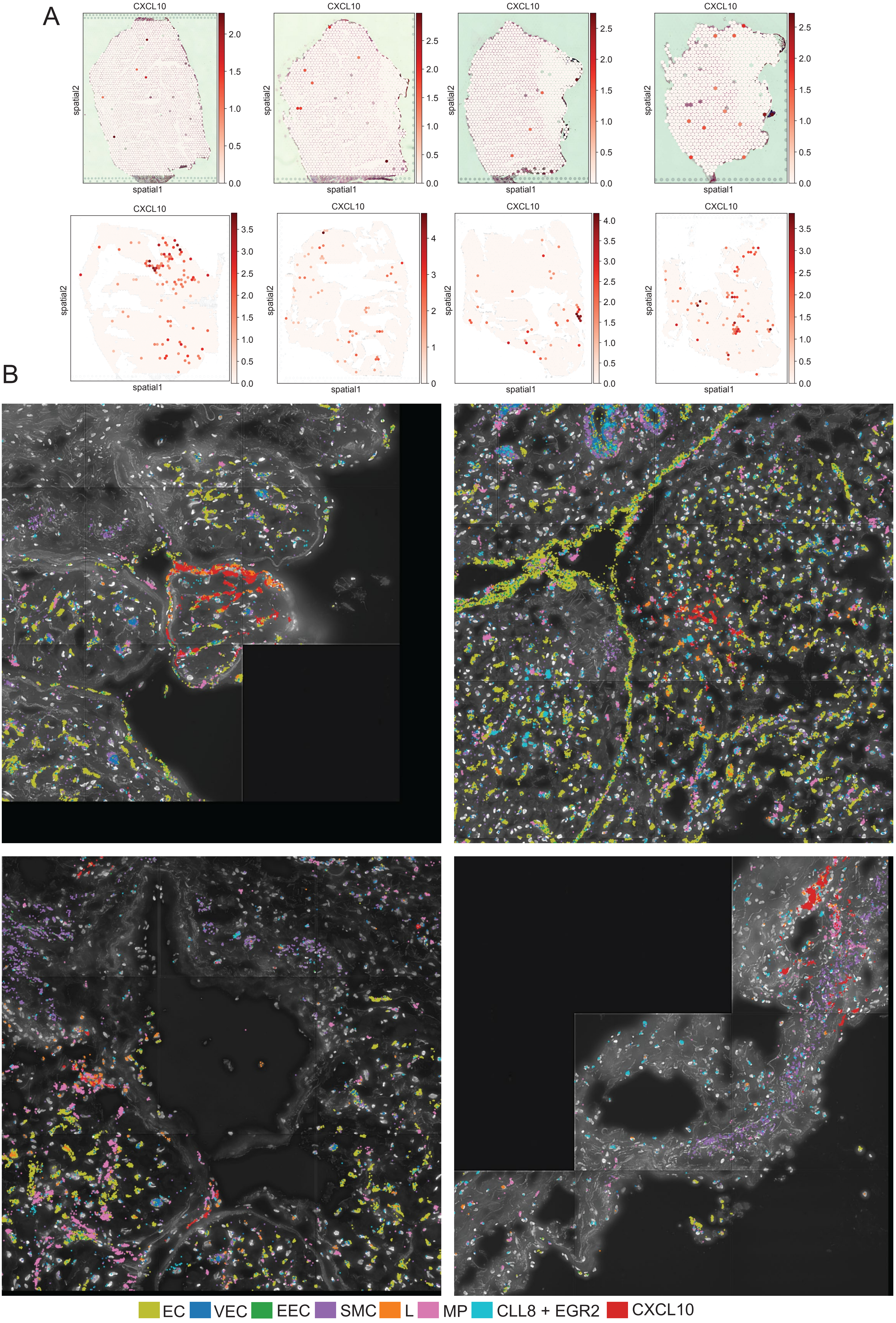
A
B
CXCL10
spatial1
spatial2
EC
VEC
EEC
SMC
L
MP
CLL8 + EGR2
CXCL10

Fig. S8. Spatial distribution of CXCL10 expression. Related to Figure 6.

A. Expression of *CXCL10* on Visium slides, from a patient with heart failure and a control sample (*n*=4). **B.** Spatial images (Resolve Molecular Cartography) for EC (*EMCN, ERG, PECAM1, CDH5, VWF*), VEC (*DKK2, ENPP2, PCSK5, CYYR1*), EEC (*PCDH7*), SMC (*NTRK3, MRVII*), L (*BCL11B, CD247, SKAP1, THEMIS*), MP (*CD163, MRC1, F13A1, MS4A6A*), inflammatory (*CCL8* and *EGR2*), and *CXCL10* gene expression in IHD (upper panels and down left) and control (down right) samples.

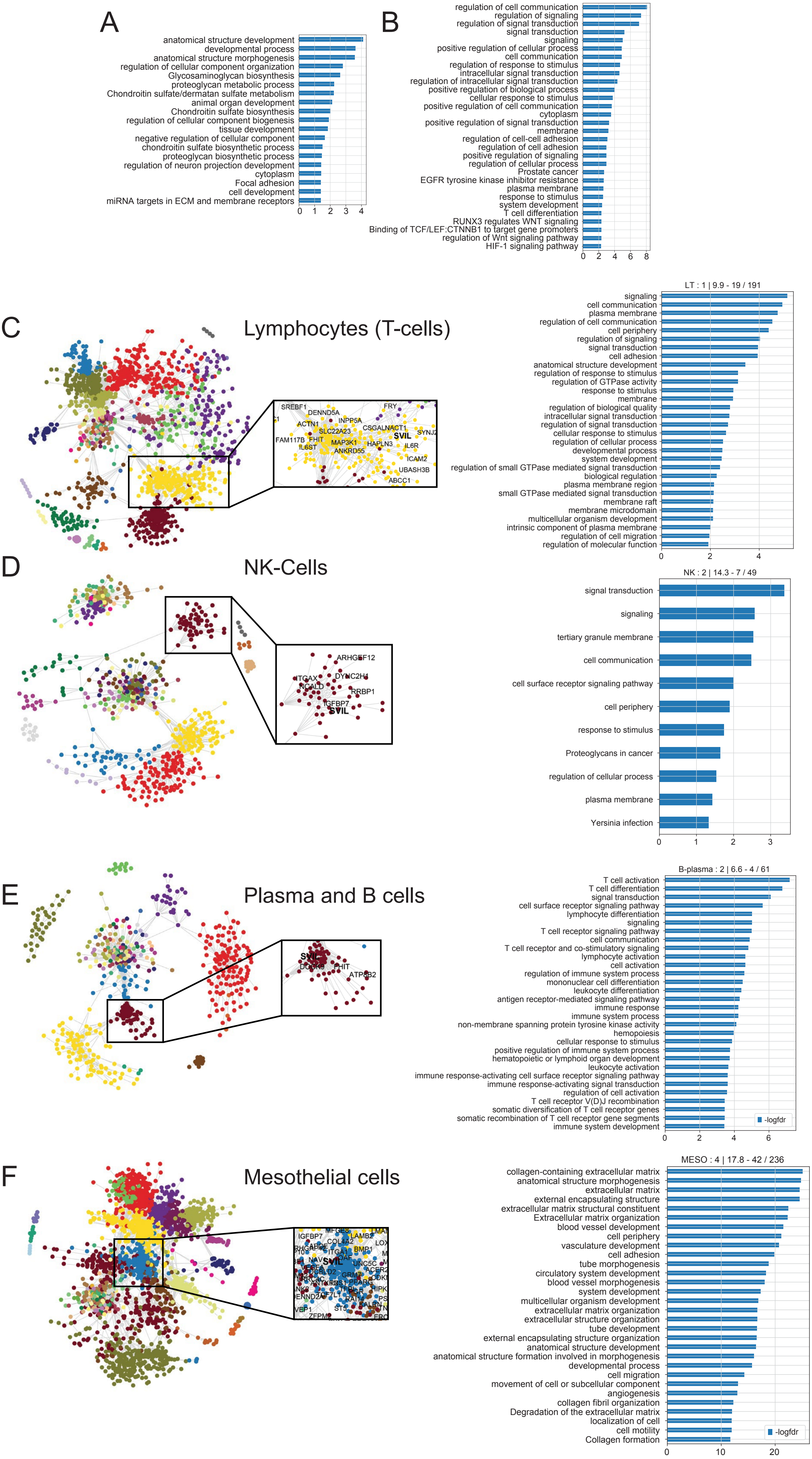
A
anatomical structure development
developmental process
anatomical structure morphogenesis
regulation of cellular component organization
Glycosaminoglycan biosynthesis
proteoglycan metabolic process
Chondroitin sulfate/dermatan sulfate metabolism
animal organ development
Chondroitin sulfate biosynthesis
regulation of cellular component biogenesis
tissue development
negative regulation of cellular component
chondroitin sulfate biosynthetic process
proteoglycan biosynthetic process
regulation of neuron projection development
cytoplasm
Focal adhesion
cell development
miRNA targets in ECM and membrane receptors
0 1 2 3 4
B
regulation of cell communication
regulation of signaling
regulation of signal transduction
signal transduction
signaling
positive regulation of cellular process
cell communication
regulation of response to stimulus
intracellular signal transduction
regulation of intracellular signal transduction
positive regulation of biological process
cellular response to stimulus
positive regulation of cell communication
cytoplasm
positive regulation of signal transduction
membrane
regulation of cell-cell adhesion
regulation of cell adhesion
positive regulation of signaling
regulation of cellular process
Prostate cancer
EGFR tyrosine kinase inhibitor resistance
plasma membrane
response to stimulus
system development
T cell differentiation
RUNX3 regulates WNT signaling
Binding of TCF/LEF:CTNNB1 to target gene promoters
regulation of Wnt signaling pathway
HIF-1 signaling pathway
0 2 4 6 8
C
Lymphocytes (T-cells)
SREBF1
DENND5A
FRY
INPP5A
ACTN1
SLC22A23
CSGALNACT1
SYNJ2
FAM117B
FHIT
SVIL
IL6ST
MAP3K1
HAPLN3
IL6R
ANKRD55
ICAM2
UBASH3B
ABCC1
LT : 1 | 9.9 - 19 / 191
signaling
cell communication
plasma membrane
regulation of cell communication
cell periphery
regulation of signaling
signal transduction
cell adhesion
anatomical structure development
regulation of response to stimulus
regulation of GTPase activity
response to stimulus
membrane
regulation of biological quality
intracellular signal transduction
regulation of signal transduction
cellular response to stimulus
regulation of cellular process
developmental process
system development
regulation of small GTPase mediated signal transduction
biological regulation
plasma membrane region
small GTPase mediated signal transduction
membrane raft
membrane microdomain
multicellular organism development
intrinsic component of plasma membrane
regulation of cell migration
regulation of molecular function
0 2 4
D
NK-Cells
ARHGEF12
ITGAX
DYNC2H1
NCALD
RRBP1
IGFBP7
SVIL
NK : 2 | 14.3 - 7 / 49
signal transduction
signaling
tertiary granule membrane
cell communication
cell surface receptor signaling pathway
cell periphery
response to stimulus
Proteoglycans in cancer
regulation of cellular process
plasma membrane
Yersinia infection
0 1 2 3
E
Plasma and B cells
SVIL
DOCK9
FHIT
ATP8B2
B-plasma : 2 | 6.6 - 4 / 61
T cell activation
T cell differentiation
signal transduction
cell surface receptor signaling pathway
lymphocyte differentiation
signaling
T cell receptor signaling pathway
cell communication
T cell receptor and co-stimulatory signaling
lymphocyte activation
cell activation
regulation of immune system process
mononuclear cell differentiation
leukocyte differentiation
antigen receptor-mediated signaling pathway
immune response
immune system process
non-membrane spanning protein tyrosine kinase activity
hemopoiesis
cellular response to stimulus
positive regulation of immune system process
hematopoietic or lymphoid organ development
leukocyte activation
immune response-activating cell surface receptor signaling pathway
immune response-activating signal transduction
regulation of cell activation
T cell receptor V(D)J recombination
somatic diversification of T cell receptor genes
somatic recombination of T cell receptor gene segments
immune system development
-logfdr
0 2 4 6
F
Mesothelial cells
IGFBP7
LAMB2
BMP1
SVIL
MESO : 4 | 17.8 - 42 / 236
collagen-containing extracellular matrix
anatomical structure morphogenesis
extracellular matrix
external encapsulating structure
extracellular matrix structural constituent
Extracellular matrix organization
blood vessel development
cell periphery
vasculature development
cell adhesion
tube morphogenesis
circulatory system development
blood vessel morphogenesis
system development
multicellular organism development
extracellular matrix organization
extracellular structure organization
tube development
external encapsulating structure organization
anatomical structure development
anatomical structure formation involved in morphogenesis
developmental process
cell migration
movement of cell or subcellular component
angiogenesis
collagen fibril organization
Degradation of the extracellular matrix
localization of cell
cell motility
Collagen formation
-logfdr
0 10 20

Fig. S9. Co-expression modules of interest and associated gene-set enrichments. Related to Figure 7.

A. Gene set enrichment with Enrichr for the gene-gene co-expression module containing *SVIL* in SMC. **B.** Gene set enrichment with Enrichr of the gene-gene co-expression module containing *SVIL* in pericardial fluid cells. **C-F.** Gene-gene co-expression modules within Pericardial fluid T lymphocytes, NK cells, B and plasma cells and Mesothelial cells, in addition to a zoom in view of *SVIL*-containing modules, with their associated gene set enrichments from Enrichr.

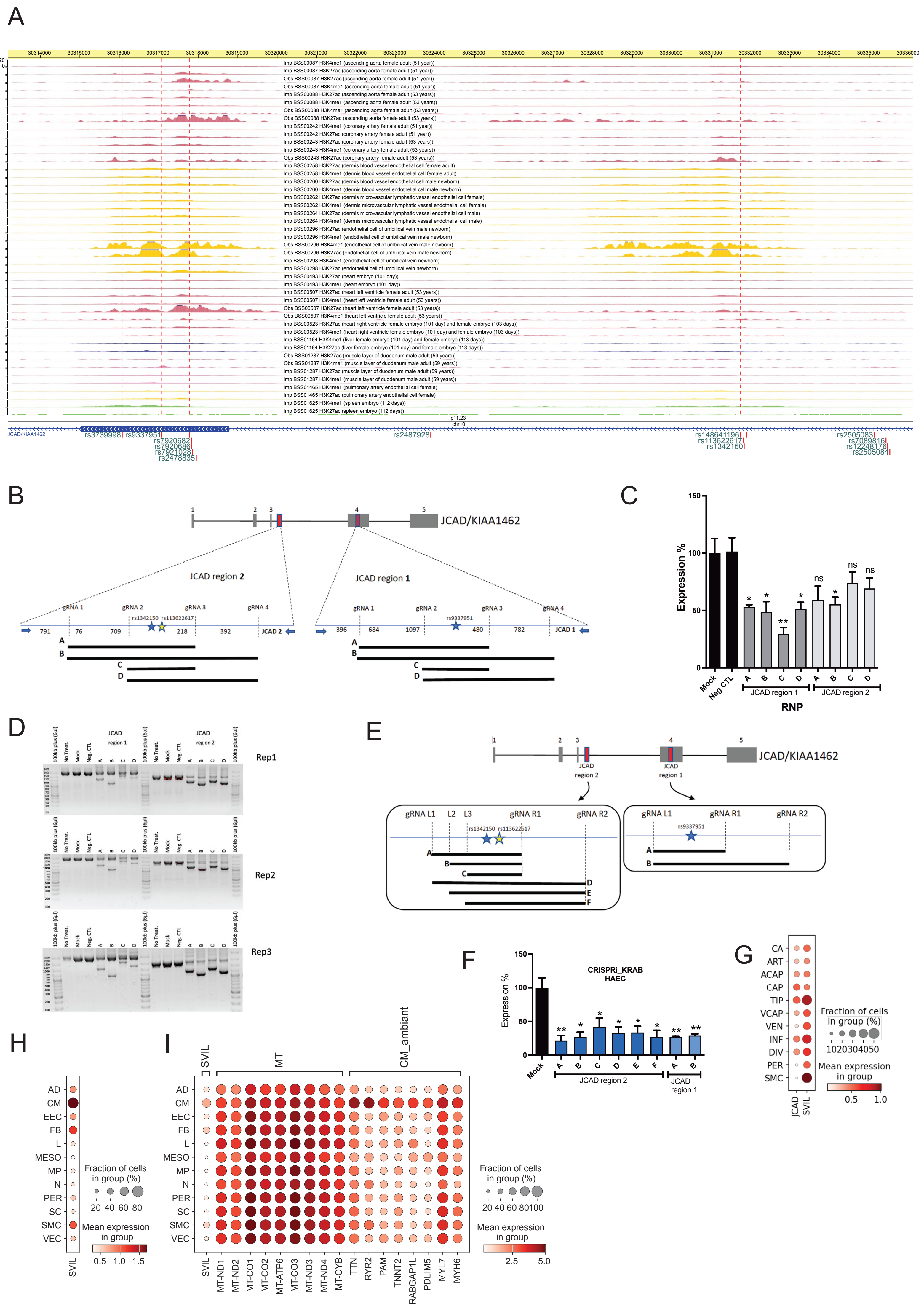
A
B
JCAD/KIAA1462
JCAD region 2
JCAD region 1
C
Expression %
JCAD region 1
JCAD region 2
RNP
D
Rep1
Rep2
Rep3
E
JCAD/KIAA1462
F
CRISPRi_KRAB
HAEC
Expression %
JCAD region 2
JCAD region 1
G
Fraction of cells in group (%)
Mean expression in group
H
I
SVIL
MT
CM_ambiant

Fig. S10. Dissection of JCAD / SVIL locus genetic variants, and JCAD/SVIL expression distribution in heart tissue. Related to Figure 7.

A. Browser shot of the rs9337951 locus on chromosome 10 with histone marker signals for aorta, endothelial cells, heart, liver, muscle, and spleen. **B**. Schematic describing the regions targeted for CRISPR-mediated deletion using different combinations of two guide RNAs (gRNAs within the *JCAD*/*SVIL* locus. **C.** The effect of CRISPR deletion on *JCAD* gene expression. **D.** Confirmation of the success and efficiency of deletion using PCR. **E.** Schematic describing the regions targeted for CRISPR-mediated inhibition using different combinations of two gRNAs **F.** The effect of CRISPR inhibition on *JCAD* gene expression. **G.** *JCAD* and *SVIL* expression in tissue cells by snRNA-seq. **H**. *SVIL* expression in main cell types in snRNA-seq data. **I**. *SVIL* expression together with mitochondrial (MT) and typical ambient RNA genes in the snRNA-seq data.

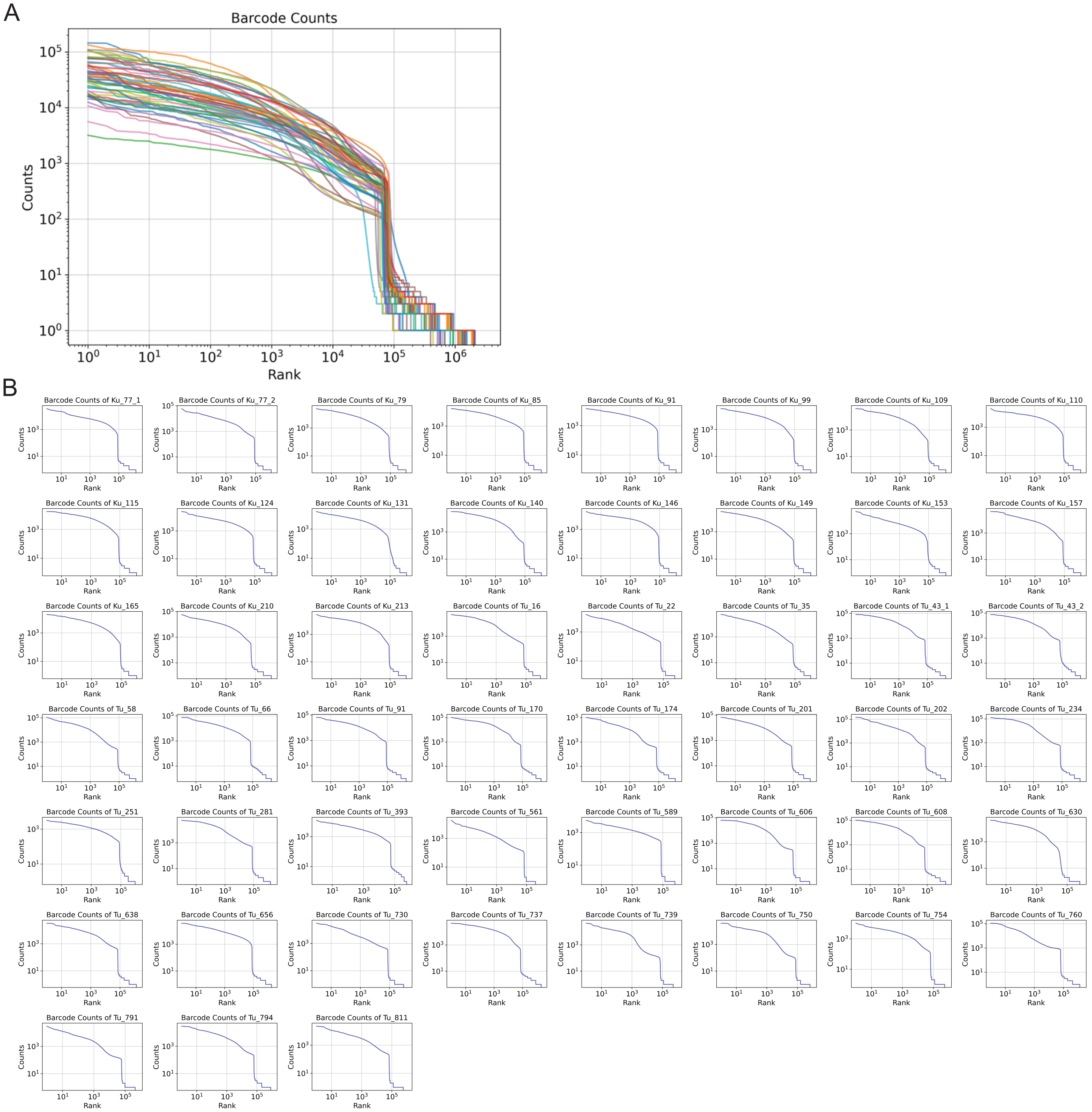
A
Barcode Counts
Counts
Rank
B
Barcode Counts of Ku_77_1
Barcode Counts of Ku_77_2
Barcode Counts of Ku_79
Barcode Counts of Ku_85
Barcode Counts of Ku_91
Barcode Counts of Ku_99
Barcode Counts of Ku_109
Barcode Counts of Ku_110
Barcode Counts of Ku_115
Barcode Counts of Ku_124
Barcode Counts of Ku_131
Barcode Counts of Ku_140
Barcode Counts of Ku_146
Barcode Counts of Ku_149
Barcode Counts of Ku_153
Barcode Counts of Ku_157
Barcode Counts of Ku_165
Barcode Counts of Ku_210
Barcode Counts of Ku_213
Barcode Counts of Tu_16
Barcode Counts of Tu_22
Barcode Counts of Tu_35
Barcode Counts of Tu_43_1
Barcode Counts of Tu_43_2
Barcode Counts of Tu_58
Barcode Counts of Tu_66
Barcode Counts of Tu_91
Barcode Counts of Tu_170
Barcode Counts of Tu_174
Barcode Counts of Tu_201
Barcode Counts of Tu_202
Barcode Counts of Tu_234
Barcode Counts of Tu_251
Barcode Counts of Tu_281
Barcode Counts of Tu_393
Barcode Counts of Tu_561
Barcode Counts of Tu_589
Barcode Counts of Tu_606
Barcode Counts of Tu_608
Barcode Counts of Tu_630
Barcode Counts of Tu_638
Barcode Counts of Tu_656
Barcode Counts of Tu_730
Barcode Counts of Tu_737
Barcode Counts of Tu_739
Barcode Counts of Tu_750
Barcode Counts of Tu_754
Barcode Counts of Tu_760
Barcode Counts of Tu_791
Barcode Counts of Tu_794
Barcode Counts of Tu_811

Fig. S11. Heart snRNA-seq barcode rank plots. Related to STAR Methods.

A-B. Heart snRNA-seq barcode rank Plots. **A**: Combined plot for all samples. **B**: Separate plot for each sample.

A

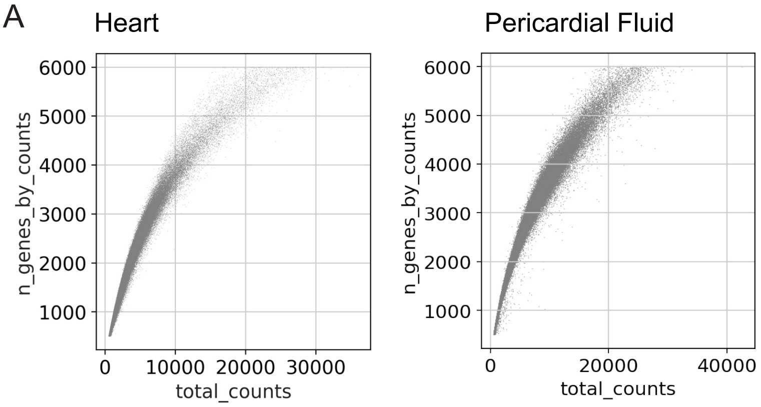
Heart
Pericardial Fluid
n_genes_by_counts
total_counts
6000
5000
4000
3000
2000
1000
0
10000
20000
30000
40000

B

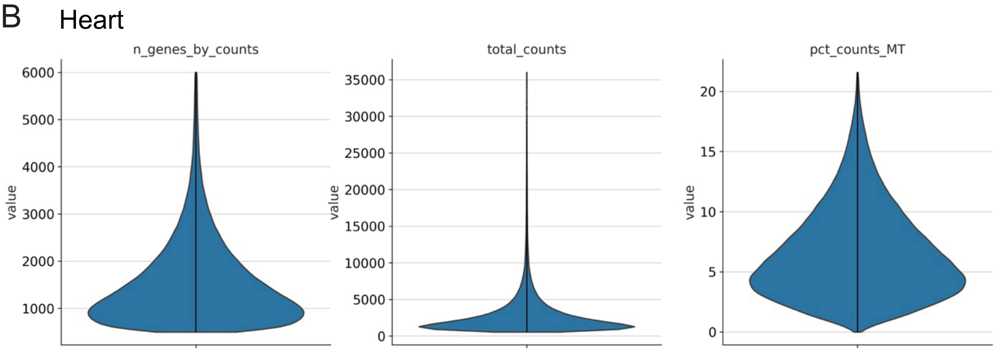
Heart
n_genes_by_counts
total_counts
pct_counts_MT
value
6000
5000
4000
3000
2000
1000
35000
30000
25000
20000
15000
10000
5000
0
20
15
10
5

Fig. S12. snRNA-seq QC metrics distribution. Related to STAR Methods.

A. Scatter plot representing the number of detected genes and the total number of counts, for each nucleus, in the heart snRNA-seq samples (left), and pericardial fluid snRNA-seq samples (right). **B.** QC metric distribution across nuclei for heart snRNA-seq samples, after filtering. From left to right: number of detected genes, total number of counts, mitochondrial expression percentage.

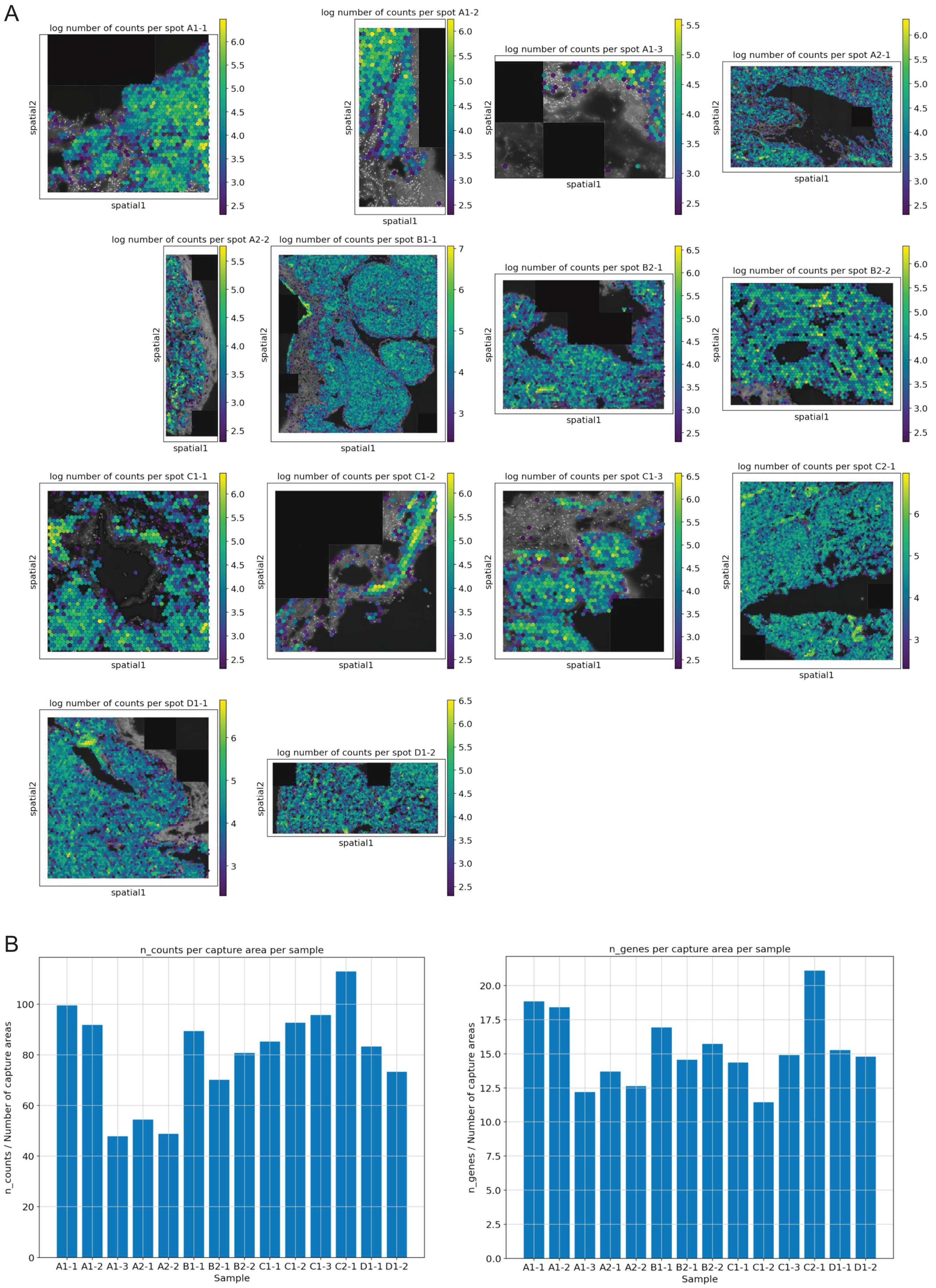
A
log number of counts per spot A1-1
log number of counts per spot A1-2
log number of counts per spot A1-3
log number of counts per spot A2-1
log number of counts per spot A2-2
log number of counts per spot B1-1
log number of counts per spot B2-1
log number of counts per spot B2-2
log number of counts per spot C1-1
log number of counts per spot C1-2
log number of counts per spot C1-3
log number of counts per spot C2-1
log number of counts per spot D1-1
log number of counts per spot D1-2
spatial1
spatial2
B
n_counts per capture area per sample
n_counts / Number of capture areas
n_genes per capture area per sample
n_genes / Number of capture areas
Sample
A1-1 A1-2 A1-3 A2-1 A2-2 B1-1 B2-1 B2-2 C1-1 C1-2 C1-3 C2-1 D1-1 D1-2

Fig. S13. Resolve spatial transcriptomics QC metrics distribution. Related to STAR Methods.

A. Distribution of number per transcript, for each Resolve spatial transcriptomics slide, in log scale. **B.** Average number of counts (left) and detected genes (right) per spot, for each Resolve spatial transcriptomics.

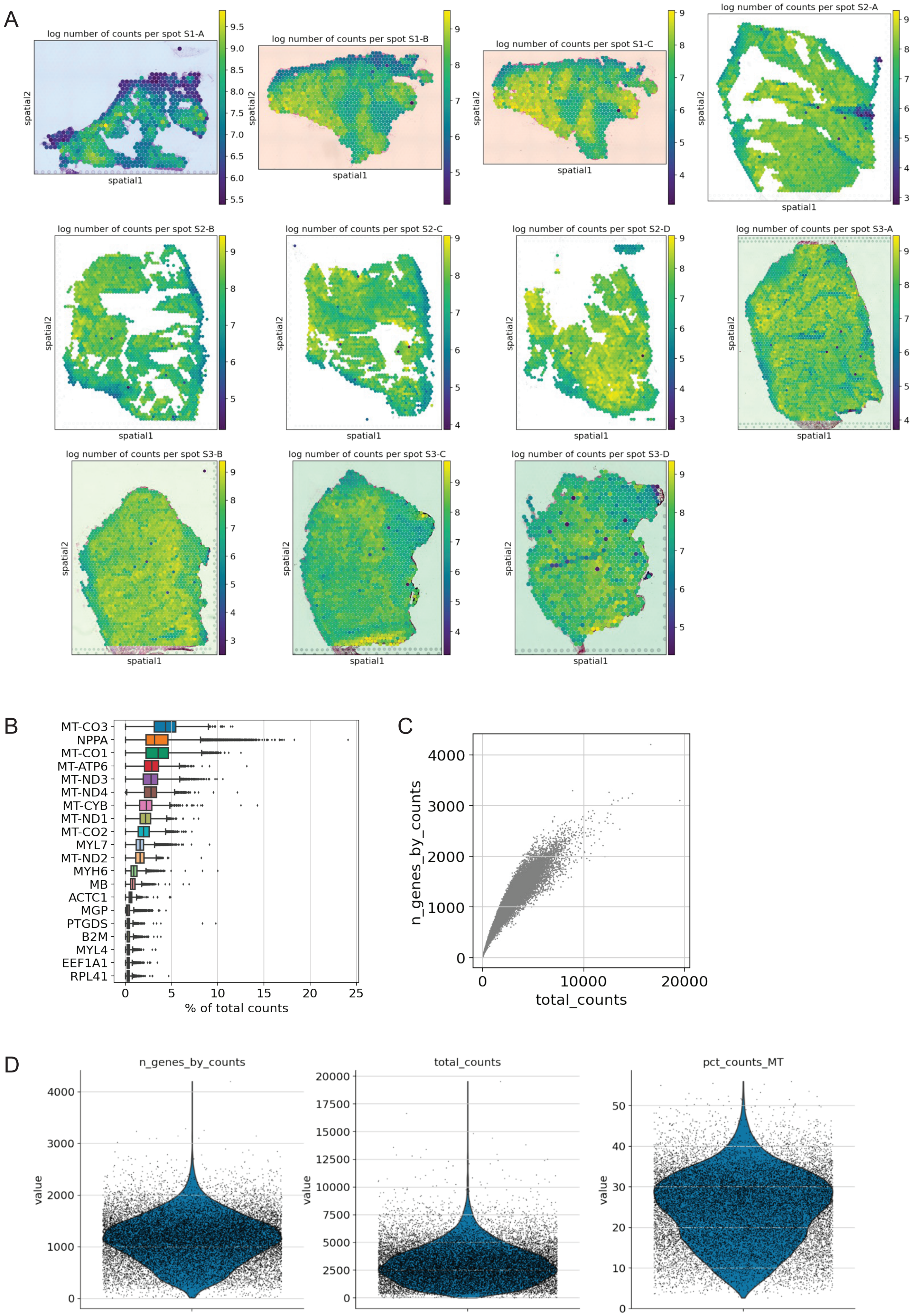
A
log number of counts per spot S1-A
log number of counts per spot S1-B
log number of counts per spot S1-C
log number of counts per spot S2-A
log number of counts per spot S2-B
log number of counts per spot S2-C
log number of counts per spot S2-D
log number of counts per spot S3-A
log number of counts per spot S3-B
log number of counts per spot S3-C
log number of counts per spot S3-D
spatial1
spatial2
B
MT-CO3
NPPA
MT-CO1
MT-ATP6
MT-ND3
MT-ND4
MT-CYB
MT-ND1
MT-CO2
MYL7
MT-ND2
MYH6
MB
ACTC1
MGP
PTGDS
B2M
MYL4
EEF1A1
RPL41
% of total counts
C
n_genes_by_counts
total_counts
D
n_genes_by_counts
total_counts
pct_counts_MT
value

Fig. S14. Visium spatial transcriptomics QC distribution. Related to STAR Methods.

A. Distribution of number per transcript, for each Visium spatial transcriptomics slide, in log scale. **B.** Expression distribution (per capture area) of the top 20 highest expressed genes, in all Visium spatial transcriptomics samples. **C.** Scatter plot representing the number of detected genes and the total number of counts, for each spot, in all Visium spatial transcriptomics samples. **D.** QC metric distribution across all spots for all Visium spatial transcriptomics samples, after filtering. From left to right: number of detected genes, total number of counts, mitochondrial expression percentage.

A

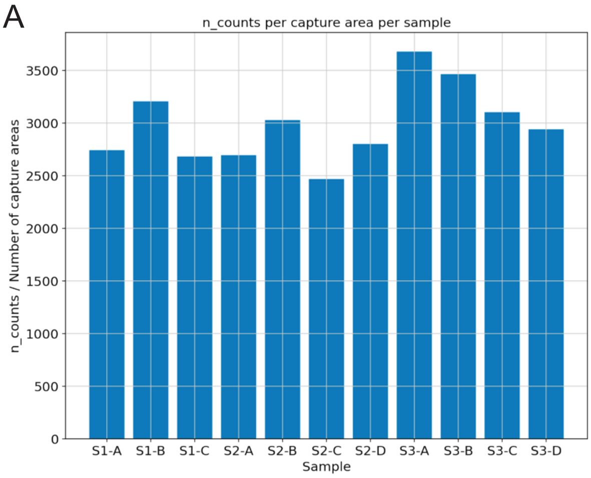

n_counts per capture area per sample
n_counts / Number of capture areas
3500
3000
2500
2000
1500
1000
500
0
S1-A
S1-B
S1-C
S2-A
S2-B
S2-C
S2-D
S3-A
S3-B
S3-C
S3-D
Sample

B

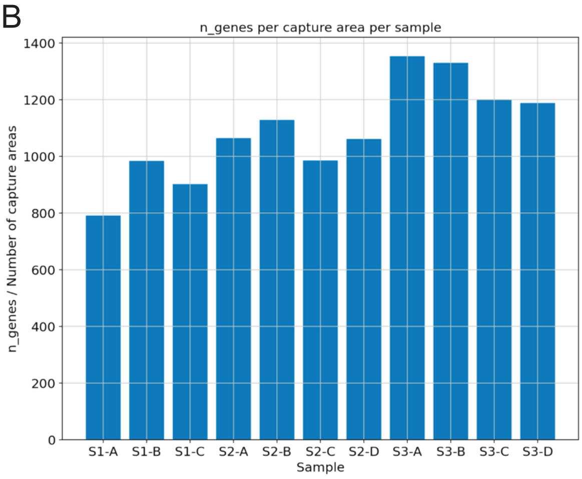

n_genes per capture area per sample
n_genes / Number of capture areas
1400
1200
1000
800
600
400
200
0
S1-A
S1-B
S1-C
S2-A
S2-B
S2-C
S2-D
S3-A
S3-B
S3-C
S3-D
Sample

Fig. S15. Distribution of the number of counts and genes for Visium spatial transcriptomics. Related to STAR Methods.

A-B. Average number of counts (**A**) and detected genes (**B**) per spot, for each Visium spatial transcriptomics sample.

Table S1. Results for allelic activity reporter assay. Related to Figure 7.

		teloHAEC 0h		teloHAEC 6h Il1b		teloHAEC 24h Il1b		HASMC chol		
RS Number	Position (GRCh37)	logFC	FDR	logFC	FDR	logFC	FDR	logFC	FDR	variant typ
rs2478839	chr10:30306804	0,029497	0,89317	-0,22841	0,594769	0,068412	0,895356	0,326823	0,203501	common
rs12762440	chr10:30306879	0,503904	0,12795	0,584236	0,479108	-0,21375	0,566648	-0,50645	0,101235	rare
rs9337951	chr10:30317073	-0,70934	0,0661	-0,22455	0,555735	-0,08465	0,895356	0,471101	0,201433	common
rs531337994	chr10:30317781	0,721114	0,0661	0,307885	0,488862	0,871545	0,16561	1,106221	0,004756	rare
rs7920682	chr10:30317826	-0,44092	0,17706	0,519276	0,479108	-0,27119	0,56637	-0,58955	0,115174	common
rs7920686	chr10:30317838	-0,44092	0,17706	0,519276	0,479108	-0,27119	0,56637	-0,58955	0,115174	common
rs7921028	chr10:30317853	-0,50678	0,33249	1,147921	0,479108	-0,40207	0,56637	-0,1748	0,75763	rare
rs2478835	chr10:30317949	0,256671	0,36864	-0,24893	0,549716	0,61482	0,264878	0,504799	0,101235	common
rs2487928	chr10:30323892	-0,16943	0,42384	0,312584	0,479108	-0,02049	0,912102	-0,26084	0,2621	common
rs148641196	chr10:30331736	-0,92915	0,00225	-1,3914	0,001085	0,108742	0,895356	-1,05572	0,004181	rare
rs113622617	chr10:30331813	0,383709	0,12795	0,696887	0,042102	0,774711	0,49638	0,681045	0,091074	rare
rs1342150	chr10:30331829	-0,4658	0,0661	-1,0913	0,003272	-0,68255	0,49638	-0,83159	0,004756	common
rs193042870	chr10:30331895	0,353719	0,18304	0,954916	0,041686	1,121558	0,332584	-0,4443	0,116393	rare
rs7089816	chr10:30335422	-0,16507	0,43332	-0,25323	0,488862	-0,15209	0,56637	-0,24679	0,2621	common
rs12248176	chr10:30335464	0,115829	0,58075	-0,05112	0,914561	-0,06663	0,895356	0,1731	0,423611	rare
rs2505084	chr10:30335520	0,396745	0,17706	0,02093	0,931401	0,307866	0,332584	0,114771	0,566492	common

Table S2A. CAREBANK samples characteristics. Related to STAR Methods.

	Control	IHD	IHF	NIHF
N	6	11	11	3
Gender	4M 2F	9M 2F	10M 1F	2M 1F
Age (years) *	67± 4	68 ± 8	66 ± 13	72 ± 6
BMI (kg/m2) *	29 ± 4	30 ± 4	28 ± 5	29 ± 4
Smoker	0	0	4	0
Atrial fibrillation	0	0	2	2
Treatment for hypertension	4	9	9	3
Treatment for diabetes	2	4	6	0
Treatment for Hypercholesterolemia	3	10	11	1
Heart failure	0	0	5	3
Prior Stroke / TIA	1	0	1	0
Previous myocardial infarction	0	0	4	0
Previous PCI	0	3	2	0
Previous CABG	0	0	0	0
Number of stenosis in the coronary arteries	0	2	2	0
LVEF*	64 ± 4	64 ± 7	38 ± 7	41 ± 9
Betablocker	2	10	9	3
Calcium blocker	0	3	4	2
ACE-inhibitor	5	7	10	3
Warfarin	0	0	3	0
NOAC	0	1	1	1
ASA	4	9	7	0
ADPB	0	1	1	0
Statin	3	10	11	1
Insuline	0	3	4	0

**Mean ± Standard deviation*

Table S2B. PERIHEART samples characteristics. Related to STAR Methods.

	**Control**	**stable CAD**	**acute MI**	**remote MI**
N	4	5	4	5
Gender	all male	all male	all male	all male
Age (years) *	61±7	68 ±4	66 ±6	72 ±6
BMI (kg/m2) *	26 ±4	26 ±3	25 ±2	28 ±3
Smoker	0	0	2	2
Atrial fibrillation	0	0	0	0
Treatment for hypertension	3	4	3	3
Treatment for diabetes	0	1	1	2
Treatment for Hypercholesterolemia	1	3	4	3
Heart failure	0	0	2	1
Prior Stroke / TIA	0	0	0	0
Previous myocardial infarction	0	0	4	5
Previous PCI	0	0	1	3
Previous CABG	0	0	0	0
Number of stenosis in the coronary arteries	0	3	3	3
LVEF *	75 ±4	61 ±7	49 ±13	48 ±9
Betablocker	2	2	3	5
Calcium blocker	0	0	2	0
ACE-inhibitor	1	1	2	4
Warfarin	0	0	0	2
NOAC	0	0	0	0
ASA	0	4	3	4
ADPB	0	0	1	1
Statin	1	4	3	5
Insuline	0	0	0	0

**Mean ± Standard deviation*
